# Ambipolar Charge Transport in Perovskite CsPbBr_3_ γ‐Ray Detectors with Superior Uniformity and Spectral Resolution by Zone Refining Processing

**DOI:** 10.1002/advs.202501875

**Published:** 2025-04-25

**Authors:** Bao Xiao, Yuquan Wang, Ning Ding, Haoming Qin, Nannan Shen, Xuchang He, Qihao Sun, Yihui He

**Affiliations:** ^1^ State Key Laboratory of Radiation Medicine and Protection School of Radiation Medicine and Protection Collaborative Innovation Center of Radiological Medicine of Jiangsu Higher Education Institutions Soochow University Suzhou 215123 China

**Keywords:** γ‐ray spectroscopy, ambipolar charge transport, perovskite CsPbBr_3_ semiconductor, uniformity, zone refining

## Abstract

Perovskite semiconductor cesium lead bromide (CsPbBr_3_) has demonstrated great promise as a new‐generation gamma‐ray detector. However, substantial challenges still present in reproducibly achieving optimal spectroscopic performance. The specific strategy for producing spectroscopic‐grade CsPbBr_3_ crystals with high reproducibility and uniformity are still not clarified. Herein, efficient zone refining processing is developed for CsPbBr_3_ crystals that facilitates impurity segregation to achieve an ultrahigh purity level of ≈1.42 ppm, therefore lowers trap density and balances charge transport. In a typical 30 mm diameter zone‐refined CsPbBr_3_ ingot, all wafers exhibited remarkable energy resolutions of 6–12% and 3–8% for ^241^Am and ^57^Co γ‐rays under comparable electric fields. The crystals also exhibited an ambipolar charge transport characteristic, resemble to elemental semiconductors, with equivalent hole and electron mobility‐lifetime products averaging 5.42 × 10^−3^ and 2.27 × 10^−3^ cm^2^∙V^−1^, respectively. Consequently, over 95% of wafers achieved energy resolutions below 5% whereas 70% exceeded 3% for ^137^Cs γ‐rays, demonstrating exceptional reproducibility and uniformity. Notably, a champion energy resolution of 1.3% with an outstanding photopeak‐to‐Compton (P/C) ratio of ≈5.3 is attained in an ambipolar planar detector. It is anticipated that this work shall expedite scalable manufacturing and practical applications of CsPbBr_3_ detectors.

## Graphical abstract

Zone refining processing has been proposed to achieve ultrahigh purity level in perovskite CsPbBr_3_ crystals, enabling ambipolar charge transport by enhancing the electron transport properties, thereby resulting in better γ‐ray detector performance with exceptional uniformity and reproducibility. A record ER of 1.3% for ^137^Cs γ‐rays is achieved with an outstanding photopeak‐to‐Compton ratio of 5.3 in an ambipolar planar CsPbBr_3_ detector.

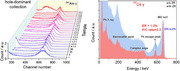

## 1 Introduction

Semiconductor γ‐ray detectors with high sensitivity and energy resolution (ER) have broad and surging applications in homeland security, medical imaging, deep space exploration, and fundamental scientific research.^[^ ^1^ ^]^ However, the development of novel semiconductors affording satisfactory overall performance is strenuous historically. High‐purity germanium (HPGe) γ‐ray detectors can deliver an unparalleled ER of 0.2–0.3% at 662 keV at liquid nitrogen temperatures.^[^ ^2^ ^]^ Halide detectors TlBr and HgI_2_ exhibit promising performances notwithstanding, materials and device studies on polarization effects and stability are still undergoing.^[^ ^3^ ^]^ Commercially available CdZnTe (CZT) detectors currently offer competitive energy resolving performance comparable to HPGe, yet progress remains constrainedly slow with limited detector size and persistently high manufacturing costs.^[^ ^4^ ^]^ Recently the resurgence of lead‐based halide perovskites has garnered tremendous research interests for nuclear radiation detection.^[^ ^5^ ^]^ Since the first report in 2013, CsPbBr_3_, with suitable bandgap, large effective atomic number, high resistivity, and favorable carrier mobility‐lifetime products, has demonstrated impressive detection performance through materials and devices advancements.^[^ ^5^, ^6^ ^]^ Planar devices fabricated from melt‐ and solution‐grown CsPbBr_3_ crystals have achieved ERs of 3.8% and 5.5% for ^137^Cs γ‐rays at 662 keV, respectively.^[^ ^5^, ^7^ ^]^ The CsPbBr_3_ detector employing a unipolar hole‐only sensing approach has demonstrated an ER of 1.4% at 662 keV, comparable to commercial CZT detectors.^[^ ^8^ ^]^

In general, the number of charge carriers generated by a single γ photon is relatively limited, enabling them susceptible to trapping or recombination by electrically active defects and impurities. It is worth mentioning that, the purity level of state‐of‐the‐art CsPbBr_3_ crystal is typically ≈5 N, which is at least two orders of magnitude lower than that of CZT (≈7 N) and HPGe (≈12 N).^[^ ^7^, ^9^ ^]^ Chemically speaking, the soft and dynamic lattices structure of halide perovskites implicates numerous defect formation sites and incorporation probabilities from both intrinsic and extrinsic impurities. Therefore, the purity of precursor materials is critically important in practice, as even trace levels of impurities at parts per million (ppm) can substantially alter the charge transport, thereby degrade detector performance. Previous studies have demonstrated the electron trapping issue and inferior electron transport in melt‐grown single crystals.^[^ ^8^ ^]^ In solution‐grown crystals, elevated impurity levels may restrict the ER of CsPbBr_3_ γ‐ray detectors, and a recrystallization process involving PbBr_2_∙2DMSO has been employed to mitigate impurity concentrations and enhance detector performance.^[^ ^10^ ^]^ However, the efficacy of this solvent‐based purification method is constrained by the absence of ideal solvents and the comparable solubility of certain impurities to the desired product. On the other hand, higher purity in precursor materials can be more readily achieved through zone refining in melt‐grown crystals. Nevertheless, few attempts have been carried out on precursor purification in CsPbBr_3_,^[^ ^11^ ^]^ and the impact of impurity levels on detector performance remains to be thoroughly explored.

Moreover, for a γ‐ray detector to be practically viable, the crystal growth process must be scalable to sufficient dimensions while ensuring consistent charge transport properties and exceptional uniformity and reproducibility in ER. Unfortunately, current reports on the performance of CsPbBr_3_ detectors demonstrate marked variability, with ERs ranging dramatically from as low as 12.85% to 1.4% for 662 keV ^137^Cs γ‐rays, despite using precursor materials with similar nominal purity levels (≈5N).^[^ ^8^, ^12^ ^]^ The underlying mechanisms responsible for the detector performance regulation remain debatable, raising substantial concerns regarding reproducibility. Despite its defect tolerance characteristic, structural defects in CsPbBr_3_ crystals are prone to form during the crystal growth process, potentially leading to unbalanced charge transport and thus non‐uniform responses, a crucial issue that has yet to be thoroughly investigated. In consequence, though prominent progresses in γ‐ray detection have been achieved with perovskite CsPbBr_3_, great challenges related to the manipulation in ambipolar charge carriers transport and detector performance uniformity especially reproducibility must be addressed.

Herein, we report the zone refining (ZR) processing for perovskite CsPbBr_3_ crystals to achieve ultrahigh purity level enabling ambipolar charge transport, thus consistent performance in γ‐ray detectors with high reproducibility and yield. Compared to unpurified CsPbBr_3_, ZR‐treated crystals demonstrated greatly improvements in optoelectronic properties, as the ERs improved from 11.7%, 8.0%, and 8.3% to 6.4%, 3.1%, and 2.5% for ^241^Am, ^57^Co and ^22^Na γ‐rays, respectively. The uniformity was assessed for a typical ZR‐treated CsPbBr_3_ ingot (Φ30 mm × 74 mm) in both electron and hole collection modes. Electron performance depicted slight spatial variability, with ERs ranging from 7% to 37% for ^241^Am and 4% to 11% for ^57^Co γ‐rays, likely attributed to trapping by impurities and related defects. Conversely, holes demonstrated superior and consistent performance, yielding ERs of 6 to 12% for ^241^Am and 4 to 8% for ^57^Co γ‐rays, respectively. Notably, outstanding equivalent charge transport and γ‐ray performance for both holes and electrons were achieved after ZR processing, highlighting the ambipolar charge transport characteristics of CsPbBr_3_ achieved by reducing impurity concentrations, in contrast to previous studies.^[^ ^7a^ ^]^ Furthermore, 96% of the wafers achieved ERs of 1.5–5% (70% within 1.5% to 3%) at 662 keV of ^137^Cs γ‐rays under comparable electric fields, indicating remarkable reproducibility and uniformity.

## 2 Results and Discussion

**Figure 1:**
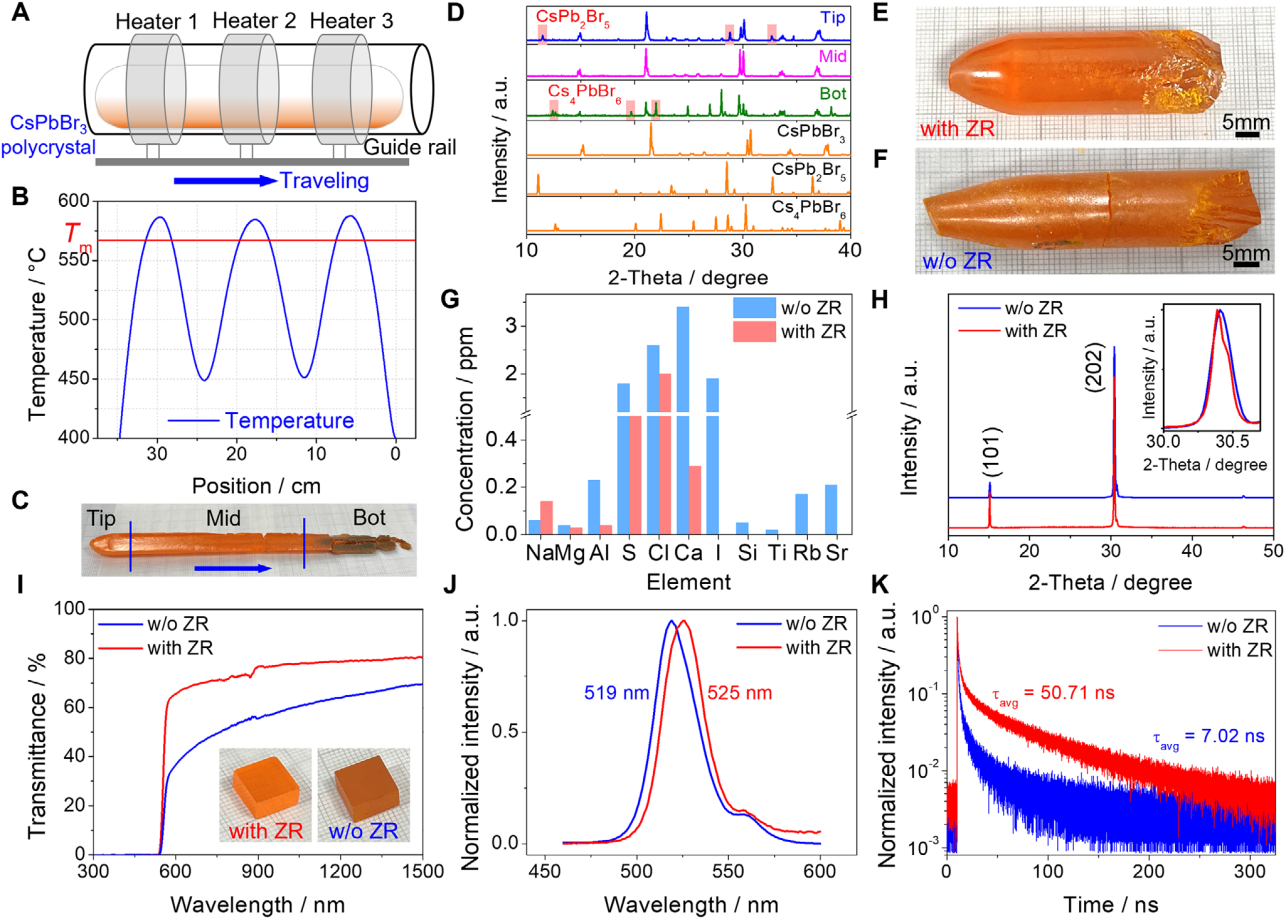
Zone refining, crystal growth, and physical properties of CsPbBr_3_. A) Schematic diagram of the three‐zone ZR furnace utilized for CsPbBr_3_ purification. B) Temperature distribution profile across the ZR furnace. C) A representative purified CsPbBr_3_ polycrystalline ingot obtained after multiple ZR cycles, the blue arrow indicating the direction of furnace travel. D) Powder XRD patterns of the Tip, Mid, and Bot regions of the purified CsPbBr_3_ ingot depicted in C). E,F) As‐grown CsPbBr_3_ ingots with and without ZR, respectively. G) Comparison of the primary impurity elements in as‐grown CsPbBr_3_ crystals with and without ZR. H) Single crystal XRD patterns of CsPbBr_3_ crystals with and without ZR. I) UV–vis–NIR transmission spectra of CsPbBr_3_ crystals with and without ZR. Insets display typical crystals with dimensions of 10 × 10 × 5 mm^3^. J,K) Steady‐state and time‐resolved PL spectra of CsPbBr_3_ crystals with and without ZR, respectively.

Zone refining purification of CsPbBr_3_ polycrystals was conducted using a custom‐built three‐zone ZR furnace (**Figure** **1**A), with the typical temperature profile depicted in Figure 1B. Following several ZR cycles, dark phases were effectively concentrated at one end of the polycrystal ingot (Figure 1C), contrasting with the uniformly distributed impurities in the synthesized pristine CsPbBr_3_ ingot (Figure S1, Supporting Information). Three distinct regions from tip (Tip), middle (Mid), and bottom (Bot) of the purified CsPbBr_3_ ingot were analyzed. The analysis revealed that the Mid region consisted of a pure orthorhombic CsPbBr_3_ phase, while Tip and Bot contained PbBr_2_‐rich CsPb_2_Br_5_ and CsBr‐rich Cs_4_PbBr_6_ phases, respectively (Figure 1D; Figure S2, Supporting Information). Note that white powder observed beneath the tip was also confirmed as CsPb_2_Br_5_ phase through EDS analysis (Figure S3, Supporting Information). According to CsBr‐PbBr_2_ pseudo‐binary phase diagram, CsPb_2_Br_5_ phase preferentially forms at the solid‐liquid interface due to constitutional supercooling during ZR process, similar to what occurs in melt‐grown CsPbBr_3_ crystals.^[^ ^12^, ^13^ ^]^ Consequently, excess CsBr accumulates toward the bottom, leading to the formation of Cs_4_PbBr_6_ phase. Elemental impurities in general segregate toward both ends of the ingot due to non‐unity segregation coefficients. The presence of amorphous carbon in the bottom segment is likely attributed to the decomposition of residual organic reagents during manufacture and processing (Figure S4, Supporting Information).^[^ ^14^ ^]^ Ultimately, the Mid regions of the purified CsPbBr_3_ polycrystalline ingots were selected, reloaded into a quartz ampoule, sealed, and re‐synthesized. The re‐synthesized zone‐refined polycrystalline material was then utilized for the growth of CsPbBr_3_ single crystals using the vertical Bridgman method.

For comparison, CsPbBr_3_ ingots with dimensions of Φ18 mm × 65 mm (with ZR) and Φ15 mm × 65 mm (without ZR) were grown using the vertical Bridgman method (Figure 1E,F). The ampoule was descended at a rate of 1 mm∙h^−1^ through a temperature gradient of ≈10 °C∙cm^−1^. After crystal growth, the ampoule was gradually cooled to room temperature at a rate of 5 °C∙h^−1^ to minimize thermal stress. The impurity concentrations of the as‐grown CsPbBr_3_ crystals without ZR are detailed in Figure 1G and Table S1 (Supporting Information), revealing a the total impurity concentration of ≈10.48 ppm, comparable to the purity level of the raw materials (5 N).^[^ ^7a^ ^]^ The predominant impurities included S (1.8 ppm), Cl (2.6 ppm), Ca (3.4 ppm), and I (1.9 ppm), collectively constituting over 90% of the total impurities. Through ZR purification, the concentrations of these major impurity elements were significantly diminished to S (0.9 ppm), Cl (2 ppm), Ca (0.29 ppm), I (<0.5 ppm, below the detection limit), yielding a dramatically lower impurity concentration of ≈3.42 ppm (Figure 1G; Table S2, Supporting Information). In principle, the consistent concentration of Cl element after ZR implies its mutual solubility in CsPbBr_3_, suggesting a segregation coefficient close to unity. The impurity concentration was effectively reduced by ≈82%, reaching ≈1.42 ppm (≈6 N) when excluding the Cl component. Furthermore, trace impurities such as Si, Ti, Rb, and Sr, initially present at relatively low levels, were all effectively eliminated to below 0.01 ppm (below the detection limit) after ZR (Figure 1G).

Powder XRD and EDS analyses confirm that all as‐grown CsPbBr_3_ exhibit an orthorhombic structure with chemical compositions close to the stoichiometric ratio (Figures S5 and S6 and Tables S3 and S4, Supporting Information). Single crystal XRD patterns demonstrate a narrower full width at half maximum (FWHM) for CsPbBr_3_ with ZR (≈0.14°) compared to that without ZR (≈0.17°), indicating enhanced crystallization quality by ZR purification (Figure 1H). As illustrated in Figure 1I, the crystal with ZR maintains a high transmittance ≈80%, whereas the transmittance decreases from 70% to 40% without ZR. Both samples exhibit absorption edges ≈540 nm, corresponding to the same bandgaps of ≈2.28 eV (Figure S7, Supporting Information). Furthermore, the emission peak for crystal without ZR occurs at a shorter wavelength of ≈519 nm, compared to ≈525 nm with ZR (Figure 1J). Time‐resolved photoluminescence (TRPL) reveals a biexponential decay feature with a rapid component (*τ*_1_) and slow component (*τ*_2_), indicative of distinct recombination mechanisms occurring at the crystal surface and within the bulk.^[^ ^15^ ^]^ Theoretically, high defect densities typically lead to shorter carrier diffusion lengths and accelerated recombination processes, resulting in shorter PL decay times.^[^ ^16^ ^]^ In comparison, the crystal with ZR exhibits longer lifetimes (*τ*_1_ = 9.14, *τ*_2_ = 72.59, and *τ*_avg_ = 50.71 ns) than the crystals without ZR (*τ*_1_ = 1.99, *τ*_2_ = 22.31, and *τ*_avg_ = 7.02 ns), as shown in Figure 1K suggesting a decrease in non‐radiative recombination centers due to enhanced purity level.

**Figure 2:**
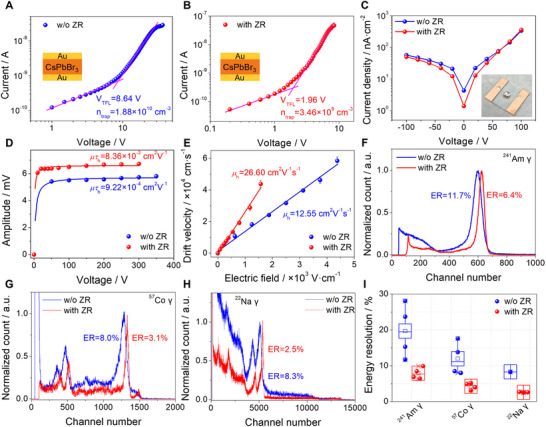
Electrical properties and γ‐ray response of CsPbBr_3_ crystals with and without ZR. A,B) Dark current–voltage curves of CsPbBr_3_ crystals without and with ZR by SCLC. C) Dark current densities of CsPbBr_3_ devices. The inset illustrates a representative EGaIn/CsPbBr_3_/Au detector. D) Hole mobility‐lifetime product (*μτ*_h_) calculated according to the Hecht equation. E) Hole mobilities (*μ*_h_) determined by linearly fitting the electric field‐dependent drift velocity. F–H) ^241^Am, ^57^Co, and ^22^Na γ‐ray spectra were obtained by the detector with and without ZR with a collecting time of 180 s. The detector dimensions are 2 × 2 × 0.8 mm^3^ (w/o ZR) and 4 × 3.5 × 1.27 mm^3^ (with ZR), respectively. I) Statistical results of the ER for different devices without and with ZR under ^241^Am, ^57^Co, and ^22^Na γ‐ray sources, respectively.

The trap density was further evaluated through space charge limited current (SCLC) measurement utilizing a standard Au/CsPbBr_3_/Au sandwich structure, as depicted in the inset of **Figure** **2**A,B. The trap densities were determined as 3.46 × 10^9^ and 1.88 × 10^10^ cm^−3^ for CsPbBr_3_ crystals with and without ZR respectively. This result also confirms that ZR purification not only reduces impurity concentration but also lowers trap density. To minimize dark current, asymmetric planar devices of EGaIn/CsPbBr_3_/Au were fabricated, as shown in Figure 2C. The rectifying characteristics observed in the dark *I–V* curves resulted in low dark current densities of ≈57.1 and ≈49.9 nA∙cm^−2^ for crystals without and with ZR, respectively (Figure 2C). The electrical resistivities were calculated to be 4.86 × 10^9^ and 8.31 × 10^9^ Ω∙cm for crystals without and with ZR (Figure S8, Supporting Information). The lower dark current density and higher electrical resistivity of CsPbBr_3_ with ZR are preferable for minimizing detector operation noise.

The charge collection efficiency is a crucial factor for radiation detectors and is accessible through the carrier mobility‐lifetime product. As illustrated in Figure 2D, the hole mobility‐lifetime product for CsPbBr_3_ with ZR is measured to be 8.36 × 10^−3^ cm^2^∙V^−1^ according to the Hecht equation using a ^241^Am γ‐ray source, which is nearly an order of magnitude greater than that without ZR (9.22 × 10^−4^ cm^2^∙V^−1^). The hole mobilities are calculated to be 12.55 and 26.60 cm^2^∙V^−1^∙s^−1^ for crystals without and with ZR, respectively (Figure 2E). This improvement in both hole mobility and the mobility‐lifetime product for CsPbBr_3_ with ZR is primarily attributed to the reduction in trap density resulting from the decreased impurity concentration. The carrier lifetime (*τ*), derived from the experimental mobility‐lifetime product and mobility, reveals the value of 73.47 and 314.29 µs for crystals without and with ZR, respectively. The significantly prolonged carrier lifetime in crystals with ZR indicates a lower density of electrically active defects.

The detectors were subsequently evaluated using various γ‐ray sources under a hole‐dominant collection mode. As shown in Figure 2F–H), the detector without ZR achieved ERs of 11.7%, 8.0%, and 8.3% for ^241^Am, ^57^Co and ^22^Na γ‐ray, respectively. In contrast, the detector with ZR exhibited dramatic improvements with ERs of 6.4%, 3.1%, and 2.5% for the same γ‐ray sources. Additionally, the photopeaks in the energy spectra with ZR also show higher channel numbers. Overall, the superior performance of CsPbBr_3_ detectors with ZR can be primarily attributed to the enhancements in charge transport and charge collection efficiency facilitated by ZR purification. The uniformity of detector performances across as‐grown CsPbBr_3_ crystals with and without ZR was also assessed by multiple detectors and various γ‐ray sources (Figures S9 and S10, Supporting Information). The consolidated results in Figure 2I and Table S5 (Supporting Information) indicate that CsPbBr_3_ detectors with ZR exhibit improved ER and better uniformity.

**Figure 3:**
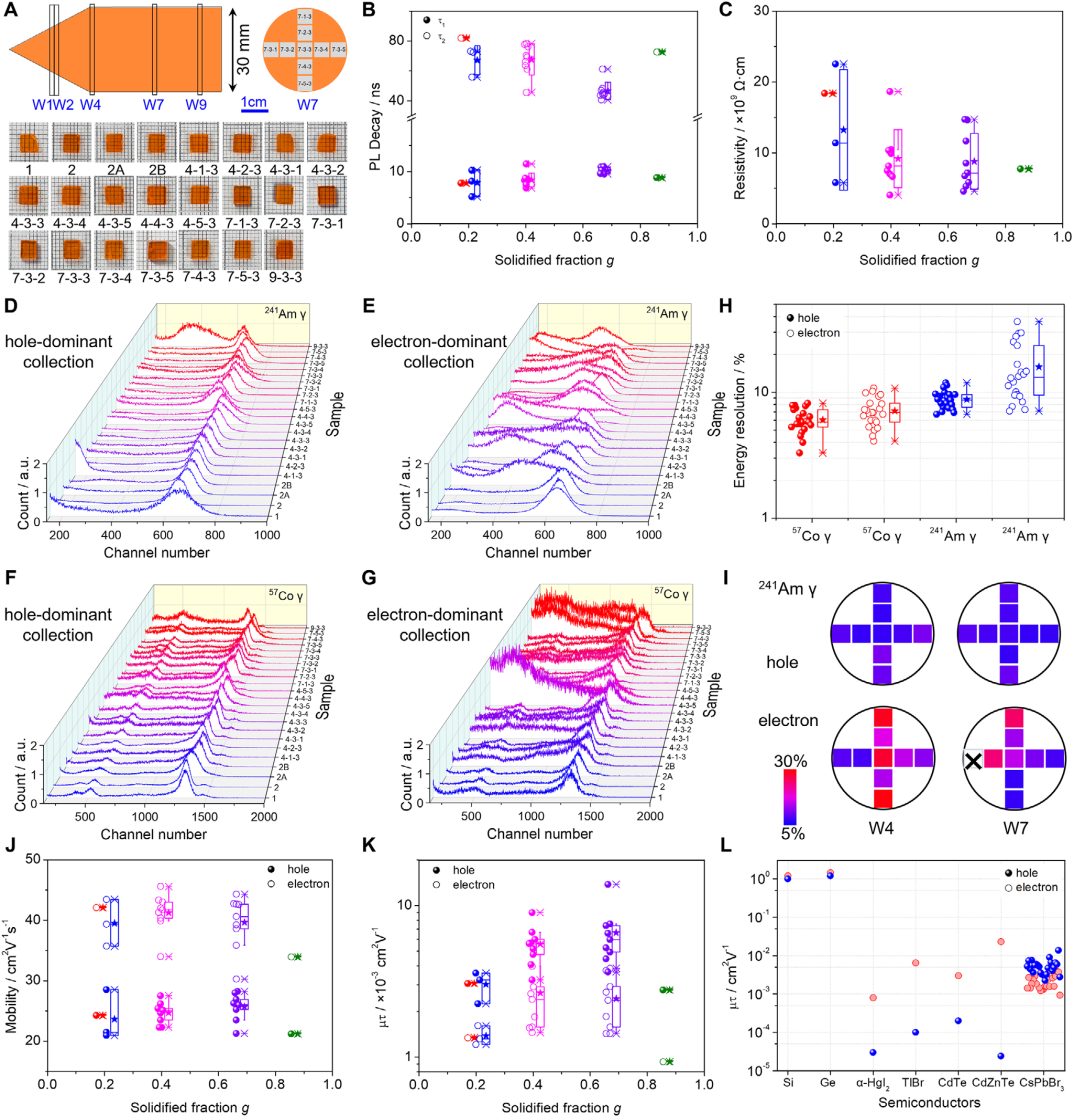
The uniformity of as‐grown CsPbBr_3_ crystals with ZR. A) Schematic diagram illustrating the cutting of various wafers in both axial and radial directions and corresponding wafer samples. B,C) Statistical results of PL decay lifetimes and resistivities versus solidified fraction for CsPbBr_3_ wafers. D–G) ^241^Am and ^57^Co γ‐ray spectra were acquired by irradiation from the anode (hole‐dominant collection) and cathode (electron‐dominant collection) with a shaping time of 10 µs and a collecting time of 180 s. H) Statistical results of hole and electron ERs for various wafers under ^241^Am and ^57^Co γ‐ray sources. I) Corresponding hole and electron ER for ^241^Am γ‐rays in W4 and W7 wafers, respectively. J) Statistical results of hole and electron mobilities versus solidified fraction. K) Statistical results of hole and electron mobility‐lifetime products versus solidified fraction, respectively. L) Comparison of hole and electron mobility‐lifetime products of elemental and compound semiconductors.^[^ ^17^ ^]^

Uniformity and reproducibility are essential prerequisites for a crystal to be used as a γ‐ray detector practically. As demonstrated in Figure S11 (Supporting Information), a large ZR‐treated CsPbBr_3_ ingot with dimensions of Φ30 mm × 74 mm was grown to systematically evaluate its uniformity. The ingot was initially sliced into circular wafers along the axial (crystal growth) direction, with specific distances from the tip detailed in Table S6 (Supporting Information). These circular wafers were subsequently sectioned in the radial direction using a 9‐point die position (Figure S11, Supporting Information), resulting in a series of 23 square wafers shown in **Figure** **3**A. The PL decay lifetimes of these wafers exhibited a similar biexponential decay profile, with a rapid component of ≈10 ns and a slow component of 50–80 ns (Figure 3B; Figure S12, Supporting Information), resemble to the ZR‐treated crystal depicted in Figure 1K. All wafers exhibited high transmittance of 70%–80% with a consistent bandgap of ≈2.28 eV (Figure S13, Supporting Information). The resistivities derived from dark current–voltage curves of planar EGaIn/CsPbBr_3_/Au devices, indicated high resistivity values within the range of (5–20) ×10^9^ Ω∙cm (Figure 3C; Figure S14, Supporting Information). Collectively, these results concerning PL decay lifetime, transmittance, bandgap, and resistivity across the wafers with various locations highlight the exceptional uniformity of the as‐grown CsPbBr_3_ ingot.

The detector performance uniformity was further conducted in both hole‐ and electron‐dominant collection modes by irradiating from the anode and cathode with ^241^Am and ^57^Co γ‐ray sources, respectively (Figure S15, Supporting Information). The attenuation length for 59.5 keV γ‐rays in CsPbBr_3_ crystal is 0.42 mm, which is considerably smaller than the crystal thickness of 1.28–1.88 mm (Figure S16, Supporting Information). This allows for the approximate consideration as operating in a single‐carrier transport configuration. However, this approximation may not be applicable for 122 keV γ‐rays, given its longer attenuation length of 1.22 mm.

Figure 3D illustrates the ^241^Am γ‐ray spectra in hole‐dominant collection for all wafers, showcasing well‐resolved saturated full‐energy peaks of 59.5 keV with similar centroid positions. The low‐energy peak observed in the spectrum of wafer 9‐3‐3 is probably associated with defect‐ or impurity‐related trapping, given this wafer is situated near the bottom of the ingot, a region prone to impurity accumulation. In contrast, the γ‐ray spectra of ^241^Am collected upon electron‐domination collection configuration exhibited inconsistent spectroscopic responses, with some wafers lacking a full‐energy peak altogether (Figure 3E). The peak channel numbers varied greatly, and a low‐energy tailing effect was commonly observed across multiple wafers, indicating that electron transport is more susceptible to fluctuations in impurities concentration or other related defects. It is important to note that the impurity concentration in present CsPbBr_3_ crystals remains relatively high (≈1.42 ppm excluding Cl, corresponding to ≈6 N purity, whereas at least 7N for CZT), and the impurities are likely distributed heterogeneously due to inherent variations in the thermal field and growth dynamics. Similar trends were evident in the hole‐ and electron‐dominant collection spectra of ⁵⁷Co γ‐rays, with the hole demonstrating a more uniform and improved response, whereas the electron exhibited comparatively poorer performance (Figure 3F,G).

Nevertheless, it is worth noting that several wafers (e.g., wafers 1,2,7‐2‐3, etc.) exhibited equally outstanding γ‐ray spectra for both hole‐ and electron‐dominant collection. This contrasts with previous studies on CsPbBr_3_ crystals, which predominantly highlighted superior hole performance.^[^ ^7^, ^18^ ^]^ The improvement is largely attributed to enhanced electron charge transport, achieved by reducing impurity concentrations through ZR purification, resulting in an ambipolar charge transport behavior.

The energy resolution and charge transport properties of various wafers were assessed in details in Figures S17–S39 (Supporting Information), with the statistical results summarized in Table S7 (Supporting Information). The wafers exhibited superior ER for hole collection, with an average ER of 8.8% (median value of 8.7%) for ^241^Am γ‐rays. Conversely, the ER for electron collection was slightly worse, averaging 15.9% (median value of 13.4%), primarily due to the reduced electron charge collection efficiency from trapping effects related to the impurities and defects. For ^57^Co γ‐rays, the ER values were comparable for both charge carriers, averaging 6.0% (median value of 5.8%) for hole collection and 7.1% (median value of 6.9%) for electron collection, respectively (Figure 3H). This similarity is attributed to the large penetration depth of ^57^Co γ‐ray comparable to the thickness of CsPbBr_3_ crystals (1.28–1.88 mm), which allows for non‐negligible hole transport contributions even when irradiated from the cathode (electron‐dominant collection).

As depicted in Figure 3H, all wafers exhibited an ER below 12% for hole‐dominant collection resolving ^241^Am γ‐rays, with 87% (20 out of 23) of the wafers performing better than 10%. In addition, ≈30% (7 wafers) achieved ER better than 10% for electron collection, indicating excellent uniformity for hole collection across the CsPbBr_3_ ingot. For ^57^Co γ‐rays, this disparity was diminished, with 96% of the wafers achieving ER better than 8% for hole collection, compared to 70% for electron collection. Further, the ER for electron collection demonstrated notable variation in both axial and radial directions, whereas the variation for hole collection was minimal, as depicted in Figure 3I and Figures S40 and S41 (Supporting Information). It is anticipated that the uniformity of electron performance could be enhanced further by reducing defect density in the crystals and purifying raw materials.

To verify the uniformity differences of holes and electrons in terms of charge collection efficiency, their mobility, and mobility‐lifetime products were subsequently analyzed. The typical distributions of rise time and amplitude under various voltages are illustrated in Figure S42 (Supporting Information). The average hole mobility of the wafers was determined to be 24.80 cm^2^∙V^−1^∙s^−1^ by linearly fitting the electric field‐dependent carrier drift velocity (Figures S17–S39, Supporting Information), which is marginally lower than the electron mobility of 40.12 cm^2^∙V^−1^∙s^−1^. Statistical data in Figure 3J also indicates notable uniformity in mobility relative to the solidified fraction. Furthermore, the hole mobility‐lifetime product reached a maximum of 1.38 × 10^−2^ cm^2^∙V^−1^, with an average value of 5.42 × 10^−3^ cm^2^∙V^−1^, which is slightly higher than the electron averaging 2.27 × 10^−3^ cm^2^∙V^−1^ (Figure 3K). Nonetheless, the electron transport properties in some wafers from the Tip and Mid regions are in fact comparable to those of hole carriers (Figure S43, Supporting Information), implicating the ambipolar charge transport characteristics, typically seen in elemental Si and Ge semiconductors (Figure 3L). Importantly, the mobilities and mobility‐lifetime products for the W4 and W7 wafers (middle of ingot) exceeded those of the W1, W2 (tip of ingot), and W9 (bottom of ingot), corresponding with the better ER for ^241^Am γ‐rays (Figure S40, Supporting Information). A comprehensive summary of the detector performance for the wafers is presented in Table S7 (Supporting Information), underscoring the exceptional uniformity of hole in as‐grown CsPbBr_3_ ingot.

**Figure 4:**
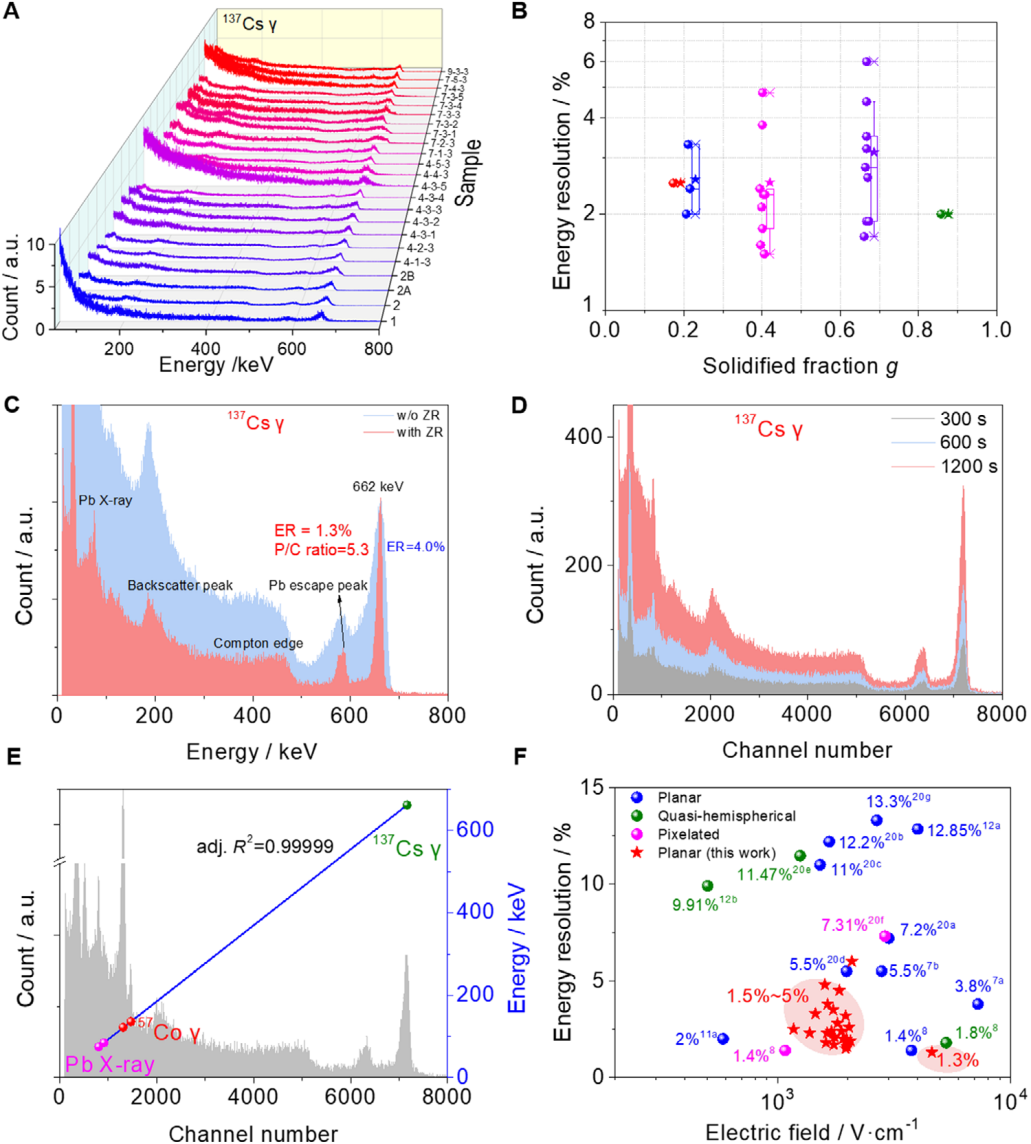
^137^Cs γ‐ray response of as‐grown CsPbBr_3_ crystals. A) ^137^Cs γ‐ray spectra of various CsPbBr_3_ wafers collected over a period of 10 min. B) Statistical results of ^137^Cs γ‐ray ER versus solidified fraction. C) Representative ^137^Cs γ‐ray spectra of CsPbBr_3_ crystals with and without ZR with a collecting time of 600 s and a shaping time of 6 µs, the applied voltage of −800 V. The detector dimensions are 2 × 2 × 0.8 mm^3^ (w/o ZR) and 5 × 5.3 × 1.74 mm^3^ (with ZR), respectively. D) Stability over a period of 20 min under ^137^Cs γ‐rays. E) The linear response to dual γ‐ray sources of ^57^Co and ^137^Cs. F) Statistical results of ^137^Cs γ‐ray ER for CsPbBr_3_ crystals with different detector structures.^[^ ^7^, ^8^, ^11^, ^12^, ^20^ ^]^

The detector performance and uniformity were further evaluated using a ^137^Cs γ‐ray source. As shown in **Figure** **4**A, all 23 wafers demonstrated superior spectroscopic responses with outstanding ER. Thereinto, 96% (22 out of 23) of the wafers achieve an ER below 5%, while 70% (16 out of 23) are better than 3% under similar electric fields (1100–2100 V∙cm^−1^), as presented in Figure 4B and Figure S44 (Supporting Information), revealing the remarkable reproducibility and uniformity of as‐grown CsPbBr_3_ ingot with ZR. Notably, the detector response remained impressively stable as the voltage increased from −400 to −800 V, with a substantial improvement in peak shape (Figure S45, Supporting Information). A best ER of ≈1.3% was attained as shown in Figure 4C, comparable to that of commercial CZT detectors.^[^ ^19^ ^]^ The spectrum also showed a high P/C ratio of ≈5.3, indicating higher photopeak detection efficiency, leveraging from the revealed ambipolar transport performance. In contrast, the performance of CsPbBr_3_ crystals without ZR was inferior, achieving a best ER of only ≈4.0% and P/C ratio of ≈2.0 (Figure 4C). Furthermore, the planar detector exhibited excellent temporal stability under −800 V (Figure 4D; Figure S46, Supporting Information). Energy linearity was also ascertained using dual γ‐ray sources, ^57^Co and ^137^Cs (Figure 4E). A summary of recent advancements in CsPbBr_3_ detectors for resolving 662 keV ^137^Cs γ‐rays is presented in Figure 4F and Table S8 (Supporting Information), highlighting the superior and uniform ERs achieved by the planar detectors after ZR purification in this work, which outperform the previous quasi‐hemispherical and pixelated CsPbBr_3_ detectors.

## 3 Conclusion

We demonstrated enhanced ambipolar charge transport and improved spectral resolution in CsPbBr_3_ crystals with remarkable reproducibility and uniformity through zone refining processing. The total impurity concentration was effectively reduced to ≈1.42 ppm (excluding mutually soluble Cl element), enabling nearly an order of magnitude enhancement in purity level, approaching ≈6 N. Hole mobility and mobility‐lifetime product greatly increased from 12.55 cm^2^∙V^−1^∙s^−1^ and 9.22 × 10^−4^ cm^2^∙V^−1^ (without ZR) to 26.60 cm^2^∙V^−1^∙s^−1^ and 8.36 × 10^−3^ cm^2^∙V^−1^ (with ZR), yielding excellent ERs of 6.4%, 3.1% and 2.5% for ^241^Am, ^57^Co and ^22^Na γ‐rays, respectively. Furthermore, a total of 23 wafers were sliced from a typical 30 mm ZR‐treated CsPbBr_3_ ingot to evaluate performance uniformity in both axial and radial directions. These wafers displayed superior and consistent performance for hole collection, with 100% and 96% of the wafers achieving ERs better than 12% and 8% for ^241^Am and ^57^Co γ‐rays, respectively. However, electron performance was slightly inferior and more variable, likely due to trapping by impurities and related defects. Impressively, the ambipolar charge transport was achieved in zone‐refined CsPbBr_3_ crystals. Besides, 96% wafers attained an ER of 1.5–5% (70% below 3%) resolving ^137^Cs γ‐rays under comparable electric fields of 1100–2100 V∙cm^−1^. An unprecedented ER of 1.3% with a P/C ratio of ≈5.3 was achieved in ambipolar planar detector. Collectively, these results reveal that CsPbBr_3_ crystals produced with ZR exhibit superior detector performance along with exceptional reproducibility and uniformity, paving the way for significant advancements in the practical applications of perovskite CsPbBr_3_.

## 4 Experimental Section

### Synthesis of CsPbBr_3_

Polycrystalline CsPbBr_3_ was directly synthesized using high‐purity CsBr (5N, Aladdin) and PbBr_2_ (5N, Aladdin). ≈50 g of the precursor materials, in a 1:1 molar ratio, were weighed and loaded into a quartz ampoule (outer diameter: 19 mm; inner diameter: 15 mm), which was then sealed under a vacuum of 5 × 10^−4^ Pa. The ampoule underwent synthesis in a tube furnace at a temperature of 600 °C for 24 h, followed by a gradual cooling period to room temperature over 12 h. Multiple ampoules of CsPbBr_3_ polycrystals were synthesized, one was allocated for direct crystal growth, while the others were reserved for ZR.

### Zone Refining

The synthesized CsPbBr_3_ polycrystals were subjected to zone refining in a custom‐built ZR furnace featuring three adjustable heaters, each ≈10 cm wide with variable spacing between adjacent heaters ranging from 0 to 5 cm. Prior to purification process, the temperature profile within the furnace was meticulously optimized by adjusting both the heater temperatures and the spacing. Subsequently, the heaters were gradually heated to the target temperature and then translated horizontally along the length of the ampoule at a controlled speed, followed by a rapid return to the starting position upon completion of the pass. During purification, the translation rate was closely correlated with the heater temperature,^[^ ^21^ ^]^ which was decreased from 100 to 10 mm h^−1^ as the temperature decreased from 630 to 580 °C. Each ampoule of CsPbBr_3_ polycrystals underwent 1–3 cycles, resulting in a total of 3–9 ZR passes.

### Crystal Growth

≈80 g of purified CsPbBr_3_ polycrystals was reloaded into a quartz ampoule (outer diameter: 23 mm; inner diameter: 18 mm) and sealed under 5 × 10^−4^ Pa. Additionally, a separate ampoule (outer diameter: 36 mm; inner diameter: 30 mm) containing 150–200 g purified polycrystals was also sealed. Both ampoules were subsequently heated to 600 °C for re‐synthesis. The synthesized CsPbBr_3_ polycrystals, both with and without ZR, were placed in a four‐zone Bridgman furnace to grow single crystal with a temperature gradient of ≈10 °C∙cm^−1^. Initially, the ampoule was held at an overheating temperature of 600 °C for 12 h to achieve homogenization prior to crystal growth. Following this, the ampoule was slowly descended along the furnace axis at a rate of 1 mm∙h^−1^, and was then cooled to room temperature at a rate of 5 °C∙h^−1^.

### Sample Processing and Device Fabrication

As‐grown CsPbBr_3_ ingots were sliced into desired wafers with a thickness of 1–2 mm using a diamond wire saw. The wafers were subsequently subjected to mechanical polishing by 1000, 2000, 5000, and 12000 mesh SiC grinding papers with mineral oil to obtain a flat and smooth surface, followed by cleaning with toluene (Figure S47, Supporting Information). Au and EGaIn electrodes were fabricated on the parallel surfaces of CsPbBr_3_ single crystals by brushing Au paint and liquid EGaIn liquid, respectively. EGaIn is a liquid eutectic Gallium‐Indium alloy comprising 75% Ga and 25% In by weight, with a melting point of ≈15.5 °C. The electrodes were then connected to the external circuitry using Cu wire.

### Structure and Electrical Characterization

Powder and single‐crystal X‐ray diffraction (XRD) patterns were obtained using a Bruker D8 Advance X‐ray diffractometer with Cu K*_α_* radiation at 40 kV and 40 mA, scanning from 10 to 40° with a step size of 0.02°. Impurity analysis was conducted via Glow Discharge Mass Spectrometry (GDMS, Nu Instrument AstruM) on as‐grown CsPbBr_3_ single crystals, both with and without ZR, leveraging the highly sensitive of this technique for detecting trace elemental impurities. Elemental composition was analyzed using a Hitachi Regulus 8230 Field Emission Scanning Electron Microscope equipped with an Oxford X‐ray energy dispersive spectrometer (EDS). UV–vis–NIR transmittance measurements were recorded over the 300–1500 nm spectral range at room temperature using a Shimadzu UV‐3150 spectrometer. Steady‐state and transient‐state photoluminescence (PL) spectra were acquired with an Edinburgh FLS1000 spectrometer. Current–voltage (*I─V*) characteristics were measured under dark conditions at room temperature using a Keithley 6517B electrometer. Space charge limited current (SCLC) measurement were employed to assessed trap density in as‐grown CsPbBr_3_ crystals, with trap‐state densities (*n*_trap_) calculated using the formula, *n*_trap_ = 2*V*_TFL_ *εε*_0_/(*ed* ^2^), where *V*_TFL_ is the trap‐filled limit voltage, *ε* is the relative dielectric constant of CsPbBr_3_ (≈23),^[^ ^22^ ^]^ and *ε*_0_, *e*, *d* are the vacuum permittivity, elementary charge, and sample thickness, respectively.

### Detector Performance Measurement

The spectroscopic performance of the detector was assessed using various γ‐ray sources, 1 µCi ^241^Am (59.5 keV), 20 µCi ^57^Co (122 keV), 20 µCi ^22^Na (511 keV), and 5 µCi ^137^Cs (662 keV) γ‐rays. For γ‐ray response, detectors were placed in an aluminum shielding box connected to an eV‐550 charge‐sensitive amplifier. A high bias, supplied by an Ortec 710 Bias Supply, was applied to the Au electrode via the eV‐550 preamplifier, while γ‐rays were irradiated from both EGaIn and Au electrodes. The preamplifier output was shaped using an ORTEC 572A amplifier, digitized by a dual 16 K input multichannel analyzer (ORTEC‐927), and processed with MAESTRO‐32 software to generate energy spectra. The mobility *μ* and mobility‐lifetime product *μ*τ could be obtained from the preamplifier pulse signals under various voltages using ^241^Am γ‐ray source. The mobility‐lifetime product was calculated from the preamplifier amplitude using the single‐carrier Hecht equation,^[^ ^23^ ^]^ *η* = *μτV*/*d* ^2^∙{1‐exp(‐*d* ^2^/*μτV*)}, where *η* is charge collection efficiency (CCE), *V* is the applied bias voltage, and *d* represents crystal thickness. Then, the average rise time based on the distribution of rise times under various voltages was used to estimate the mobility (*μ*) according to the equation, *μ* = *d* ^2^/(*t*_r_∙*V*), where *d*, *V*, and *t*_r_ are the crystal thickness, applied bias voltage, and rise time, respectively.

## Conflict of Interest

The authors declare no conflict of interest.

## Author Contributions

B.X., Y.W., and N.D. contributed equally to this work. Y.H. and B.X. conceived the experiments. B.X. and H.Q. synthesized, purified, and grew the CsPbBr_3_ single crystals. Y.W., N.D., and X.H. fabricated the detectors and characterized the detector performances. B.X., Q.S., N.S., and Y.H. wrote the manuscript. All authors discussed the results and commented on the manuscript.

## Supporting information



Supporting Information

## Data Availability

The data that support the findings of this study are available from the corresponding author upon reasonable request.

## References

[1] a) K. Iniewski , J. Instrum. 2014, 9, C11001;

[2] S. Pitale , M. Ghosh , S. Singh , G. Patra , A. Singh , M. Sonawane , S. Sen , R. Shastrakar , T. Kesarkar , K. Sudheer , BARC Newsl. 2023, 25.

[3] a) K. Hitomi , Y. Kikuchi , T. Shoji , K. Ishii , IEEE Trans. Nucl. Sci. 2009, 56, 1859;

[4] a) P. Rudolph , A. Engel , I. Schentke , A. Grochocki , J. Cryst. Growth. 1995, 147, 297;

[5] a) Y. C. Kim , K. H. Kim , D. Y. Son , D. N. Jeong , J. Y. Seo , Y. S. Choi , I. T. Han , S. Y. Lee , N. G. Park , Nature 2017, 550, 87; 28980632 10.1038/nature24032

[6] a) Y. He , Z. Liu , K. M. McCall , W. Lin , D. Y. Chung , B. W. Wessels , M. G. Kanatzidis , Nucl. Instrum. Methods Phys. Res., Sect. A. 2019, 922, 217;

[7] a) Y. He , L. Matei , H. J. Jung , K. M. McCall , M. Chen , C. C. Stoumpos , Z. Liu , J. A. Peters , D. Y. Chung , B. W. Wessels , M. R. Wasielewski , V. P. Dravid , A. Burger , M. G. Kanatzidis , Nat. Commun. 2018, 9, 1609; 29686385 10.1038/s41467-018-04073-3 PMC5913317

[8] Y. He , M. Petryk , Z. Liu , D. G. Chica , I. Hadar , C. Leak , W. Ke , I. Spanopoulos , W. Lin , D. Y. Chung , B. W. Wessels , Z. He , M. G. Kanatzidis , Nat. Photonics. 2021, 15, 36.

[9] a) J. J. McCoy , S. Kakkireni , Z. H. Gilvey , S. K. Swain , A. E. Bolotnikov , K. G. Lynn , J. Electron. Mater. 2019, 48, 4226;

[10] a) F. Wang , R. Bai , Q. Sun , X. Liu , Y. Cheng , S. Xi , B. Zhang , M. Zhu , S. Jiang , W. Jie , Y. Xu , Chem. Mater. 2022, 34, 3993;

[11] a) R. Toufanian , S. Swain , P. Becla , S. Motakef , A. Datta , J. Mater. Chem. C 2022, 10, 12708;

[12] a) M. Zhang , C. Huang , G. Xia , J. Liu , F. Tian , J. Zou , B. Tang , Acta Crystallogr., Sect. B: Struct. Sci., Cryst. Eng. Mater. 2024, 80, 64; 10.1107/S2052520624000398 38335029

[13] Y. Cheng , Q. Sun , P. Zhang , F. Wang , B. Zhang , G. Zhang , W. Jie , Y. Xu , J. Phys. Chem. Lett. 2020, 11, 5625. 32584580 10.1021/acs.jpclett.0c01561

[14] a) N. B. Singh , M. Gottlieb , T. Henningsen , R. H. Hopkins , R. Mazelsky , M. E. Glicksman , S. R. Coriell , W. M. B. Duval , G. J. Santoro , J. Cryst. Growth. 1992, 123, 227;

[15] a) A. A. Zhumekenov , M. I. Saidaminov , M. A. Haque , E. Alarousu , S. P. Sarmah , B. Murali , I. Dursun , X.‐H. Miao , A. L. Abdelhady , T. Wu , O. F. Mohammed , O. M. Bakr , ACS Energy Lett. 2016, 1, 32;

[16] a) T. Kirchartz , J. A. Márquez , M. Stolterfoht , T. Unold , Adv. Energy Mater. 2020, 10, 1904134;

[17] A. Mirzaei , J. S. Huh , S. S. Kim , H. W. Kim , Electron. Mater. Lett. 2018, 14, 261;

[18] L. Pan , Y. He , V. V. Klepov , M. C. D. Siena , M. G. Kanatzidis , IEEE Trans. Med. Imaging. 2022, 41, 3053. 35594210 10.1109/TMI.2022.3176801

[19] J. Xia , Y. Zhu , Z. He , Nucl. Instrum. Methods Phys. Res., Sect. A. 2020, 954, 161340. 10.1016/j.nima.2019.04.001 PMC7405694 32773914

[20] a) R. Bai , B. Ge , X. Liu , X. Peng , X. Zhang , S. Liu , M. Zhu , C. Zhou , A. Dubois , W. Jie , Y. Xu , J. Mater. Chem. A. 2024, 12, 13925;

[21] a) C.‐D. Ho , H.‐M. Yeh , T.‐L. Yeh , Sep. Technol. 1996, 6, 227;

[22] P. Zhang , G. Zhang , L. Liu , D. Ju , L. Zhang , K. Cheng , X. Tao , J. Phys. Chem. Lett. 2018, 9, 5040. 30102540 10.1021/acs.jpclett.8b01945

[23] P. J. Sellin , A. W. Davies , S. Gkoumas , A. Lohstroh , M. E. Özsan , J. Parkin , V. Perumal , G. Prekas , M. Veale , Nucl. Instrum. Methods Phys. Res., Sect. B. 2008, 266, 1300.

