# Supplementary material for: Ambipolar Charge Transport in Perovskite CsPbBr3 γ‐Ray Detectors with Superior Uniformity and Spectral Resolution by Zone Refining Processing
Source: Adv Sci (Weinh). 2025 Apr 25;12(26):2501875. doi: 10.1002/advs.202501875 (PMC12245115; doi:10.1002/advs.202501875)
Supplement: Supplementary file 1 — Supporting Information [file ADVS-12-2501875-s001.docx]

Supporting Information

**Ambipolar Charge Transport in Perovskite CsPbBr_3_ γ-Ray Detectors with Superior Uniformity and Spectral Resolution by Zone Refining Processing**

*Bao Xiao, Yuquan Wang, Ning Ding, Haoming Qin, Nannan Shen, Xuchang He, Qihao Sun, and Yihui He**


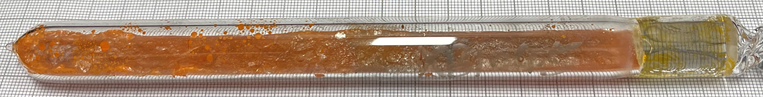


**Figure S1.** Typical ampoule of synthesized CsPbBr_3_ polycrystals.


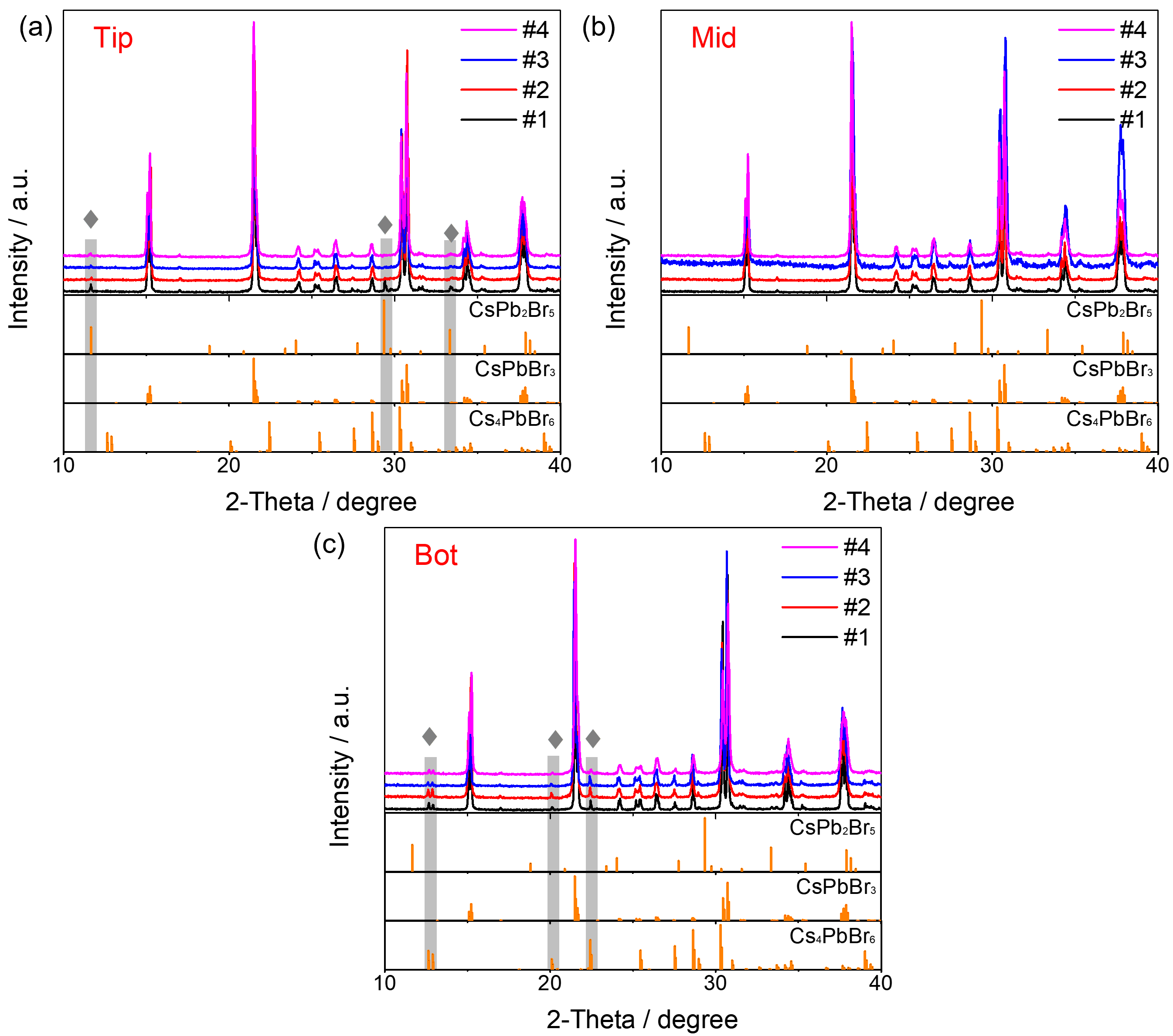


**Figure S2.** The powder XRD patterns obtained from different parts of several purified CsPbBr_3_ polycrystal ingots: (a) Tip. (b) Mid. (c) Bot.


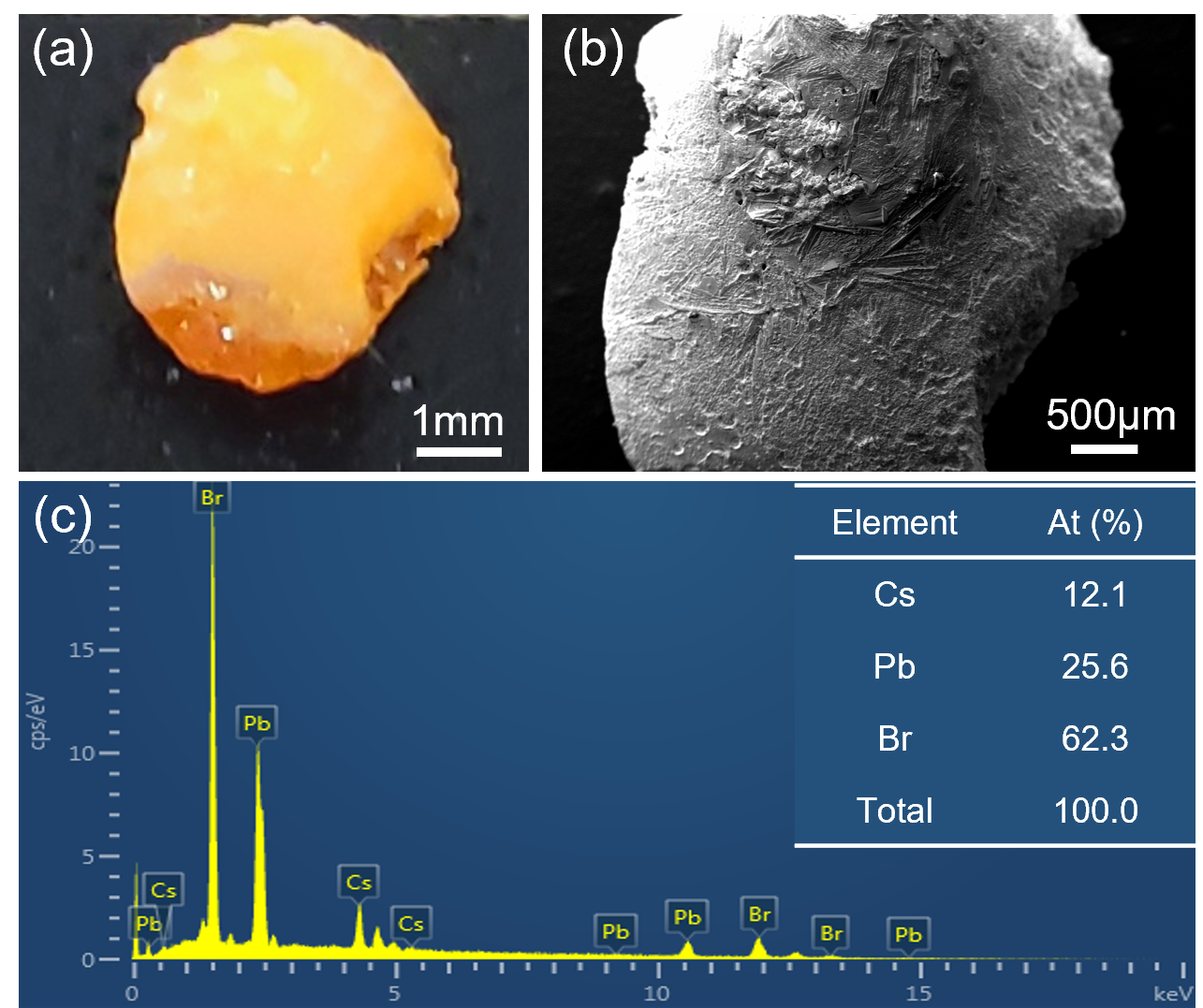


**Figure S3.** (a) A picture of the white powder obtained from Tip part of the purified CsPbBr_3_ polycrystal ingot. (b), (c) The corresponding SEM and EDS results, which indicates the white powder is CsPb_2_Br_5_ phase.


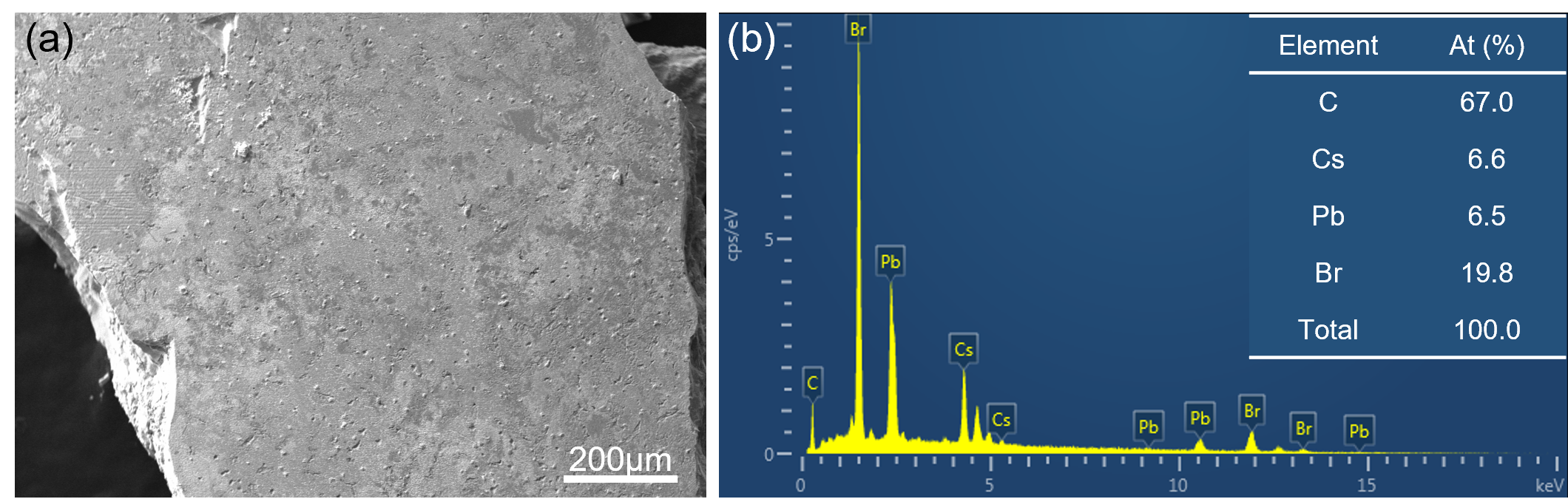


**Figure S4.** (a) Typical SEM image of the black impurity obtained from Bot part of the purified CsPbBr_3_ polycrystal ingot. (b) The corresponding EDS results, which indicates the black impurity is carbon.


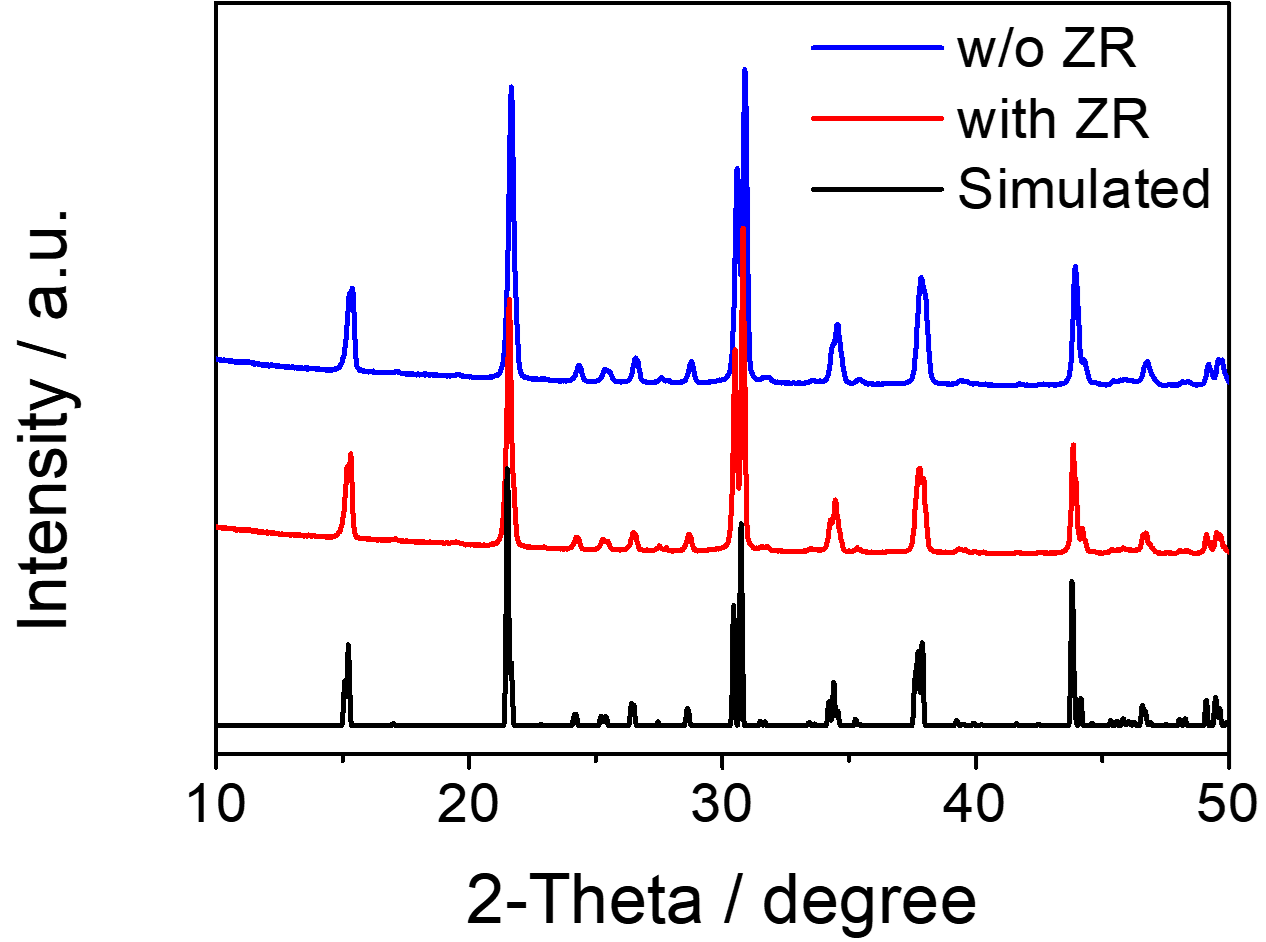


**Figure S5.** Powder XRD patterns of CsPbBr_3_ with and without ZR.


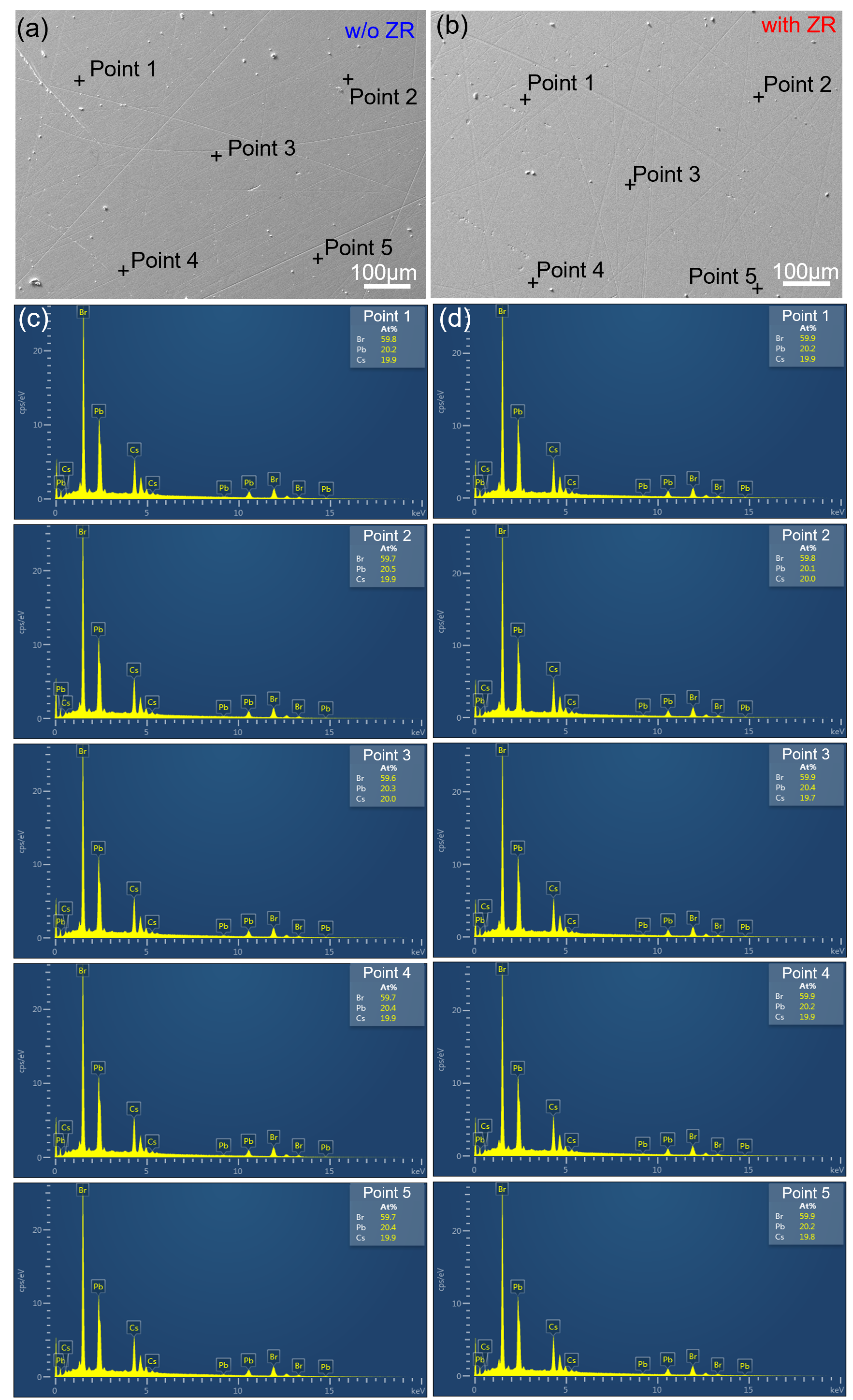


**Figure S6.** (a), (b) Typical SEM images of as-grown CsPbBr_3_ single crystals without and with ZR, respectively. (c), (d) The corresponding EDS results of CsPbBr_3_ crystals without and with ZR, respectively.


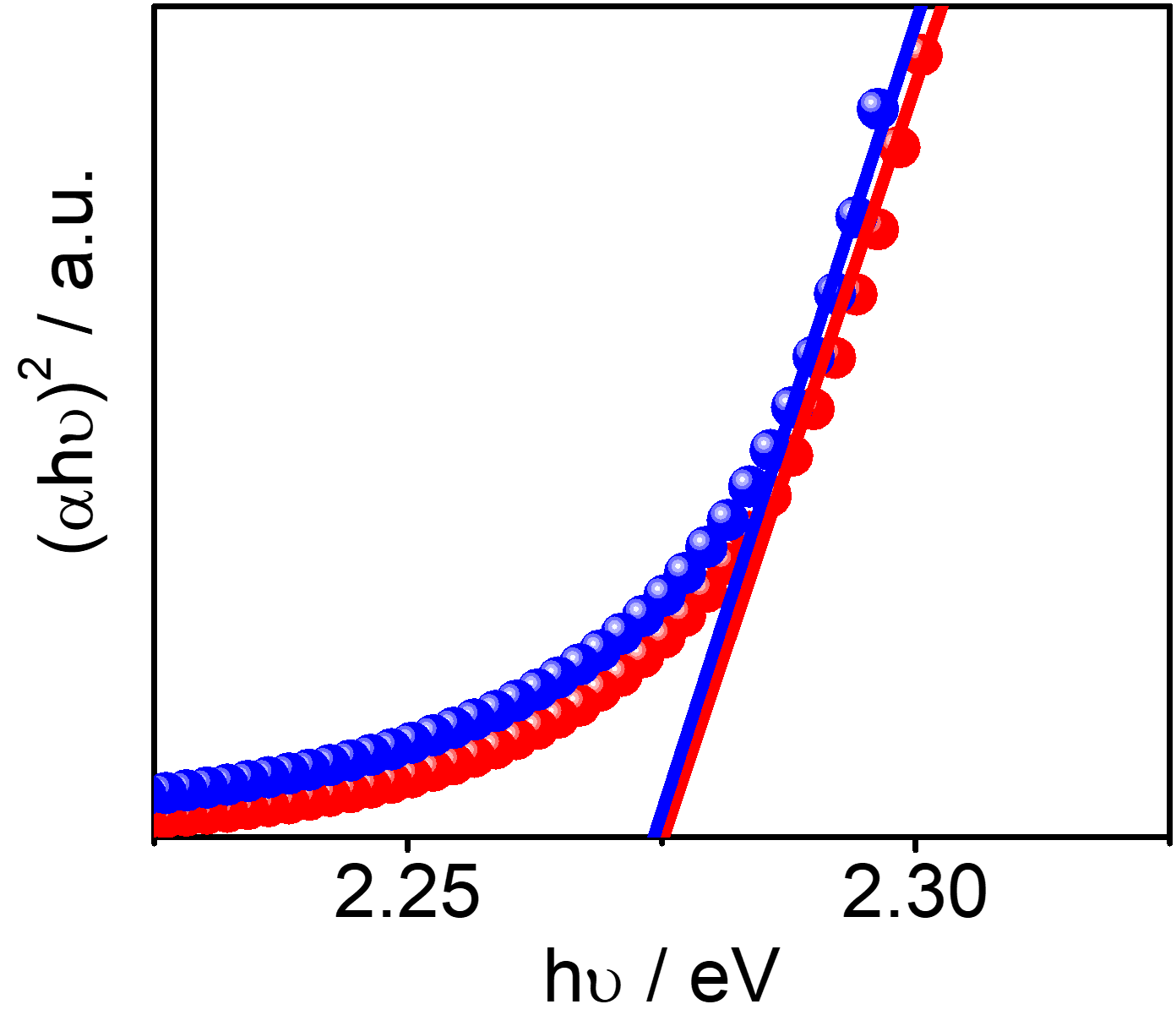


**Figure S7.** The fitted bandgaps of as-grown CsPbBr_3_ crystals without ZR and with ZR.


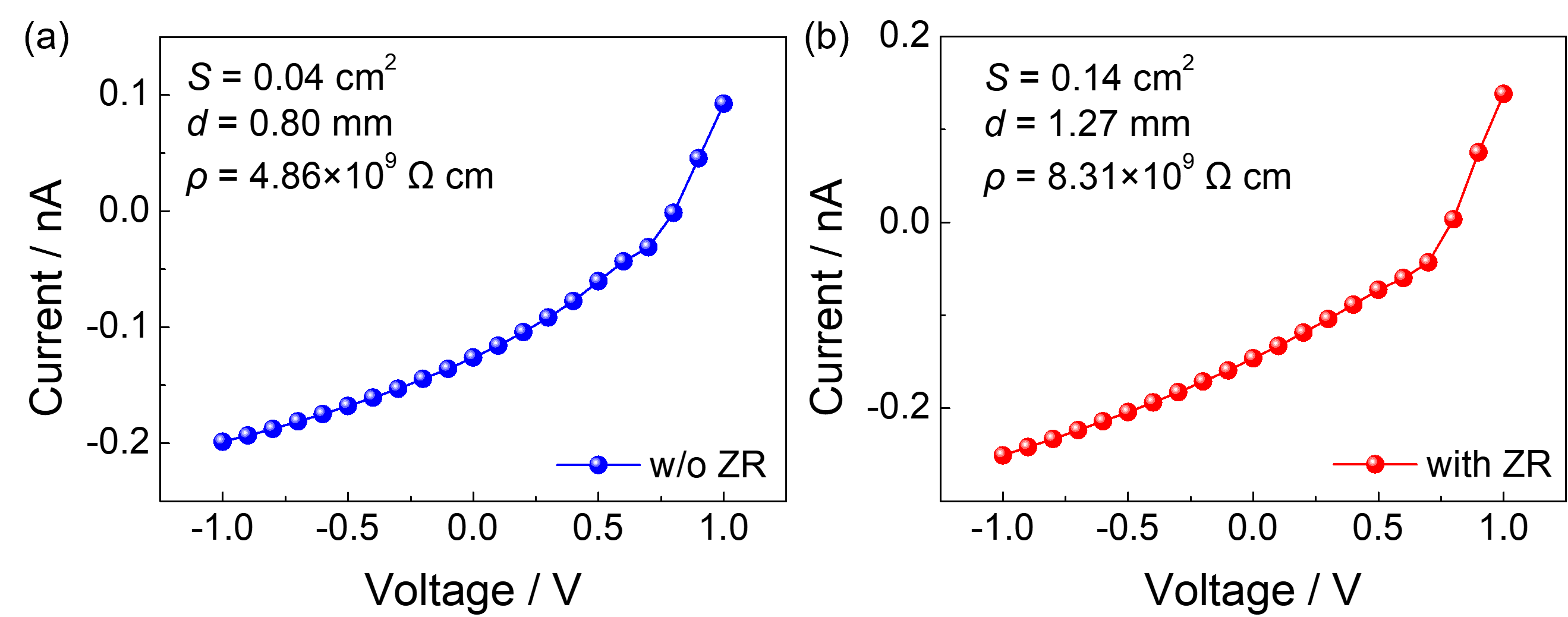


**Figure S8.** Typical dark I-V curves of CsPbBr_3_ devices: (a) without ZR, (b) with ZR.


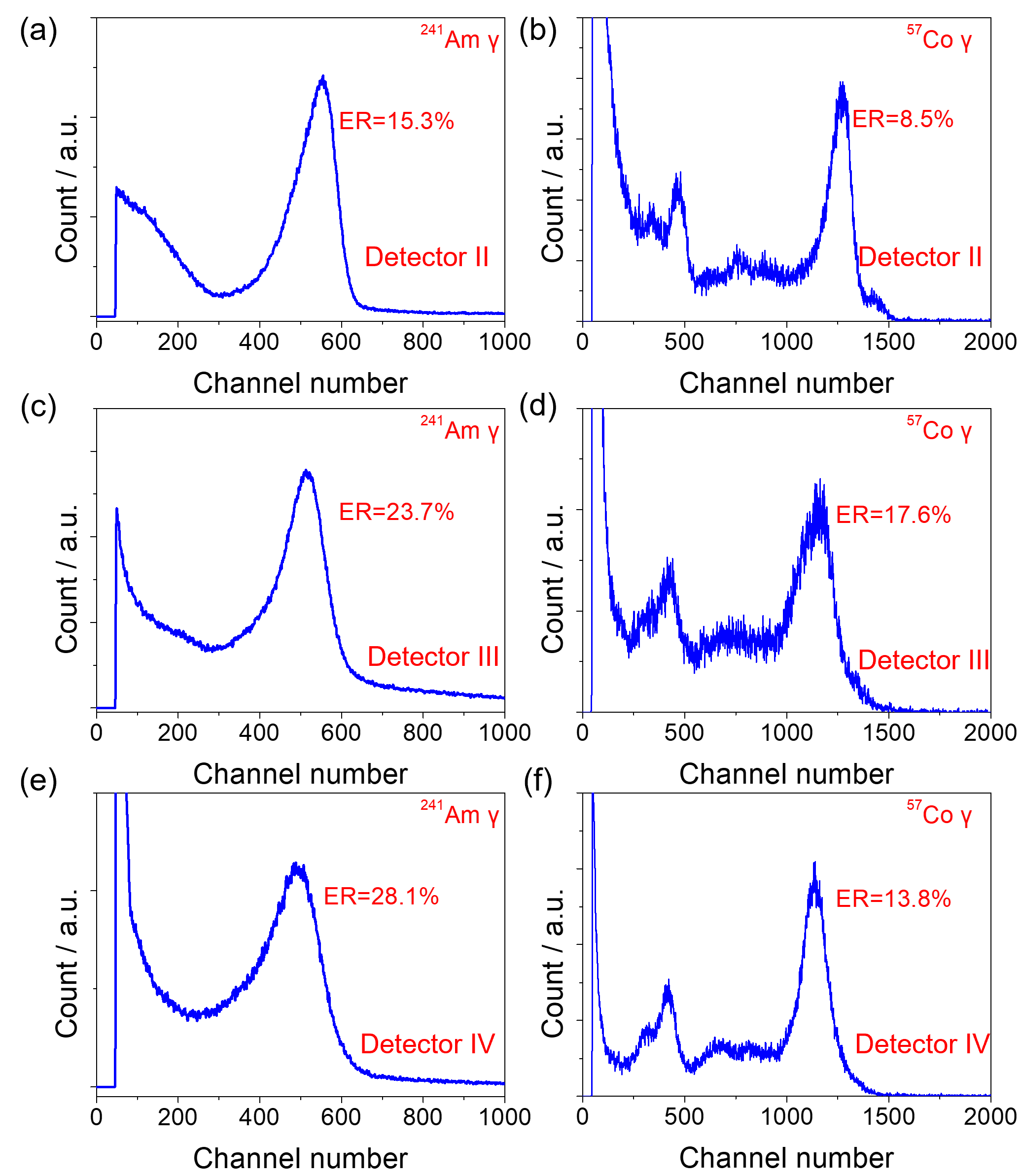


**Figure S9.** ^241^Am and ^57^Co γ-ray spectra of different CsPbBr_3_ devices without ZR with a collecting time of 180 s. The detector dimensions are 2.4×2.0×1.2 mm^3^, 3.4×2.5×1.1 mm^3^ and 3.9×3.3×1.1 mm^3^, respectively.


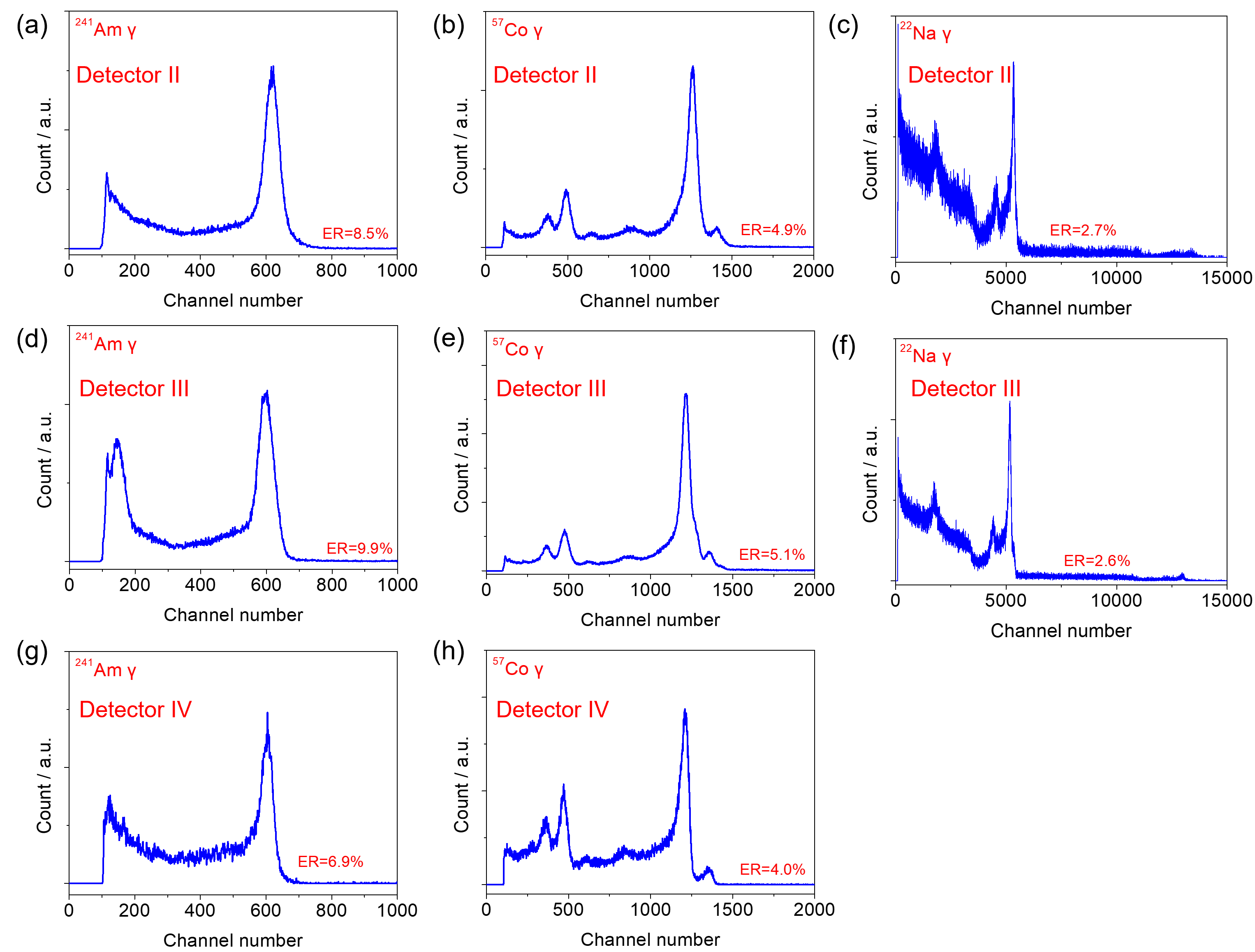


**Figure S10.** ^241^Am, ^57^Co and ^22^Na γ-ray spectra of different CsPbBr_3_ devices with ZR with a collecting time of 180 s. The detector dimensions are 4.3×4.1×1.32 mm^3^, 5.1×4.6×2.20 mm^3^ and 2.7×2.0×1.05 mm^3^, respectively.


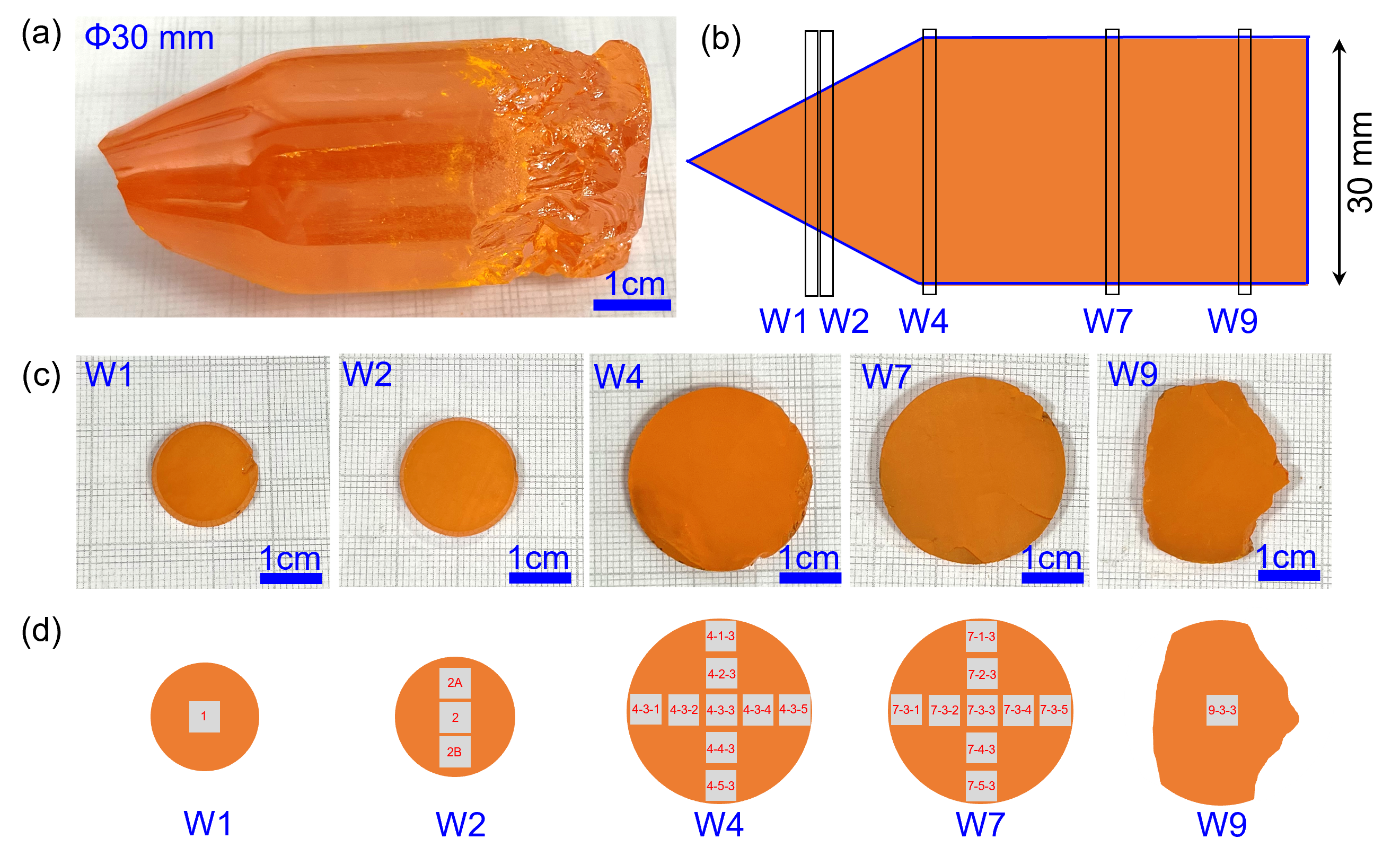


**Figure S11.** (a) As-grown CsPbBr_3_ ingot with ZR with the dimensions of Φ30 mm × 74 mm. (b) The corresponding schematic diagram of different cutting wafers along axial (crystal growth) direction. (c) The corresponding cutting wafers W1, W2, W4, W7 and W9, respectively. (d) The corresponding schematic diagram of different cutting wafers in radial direction.


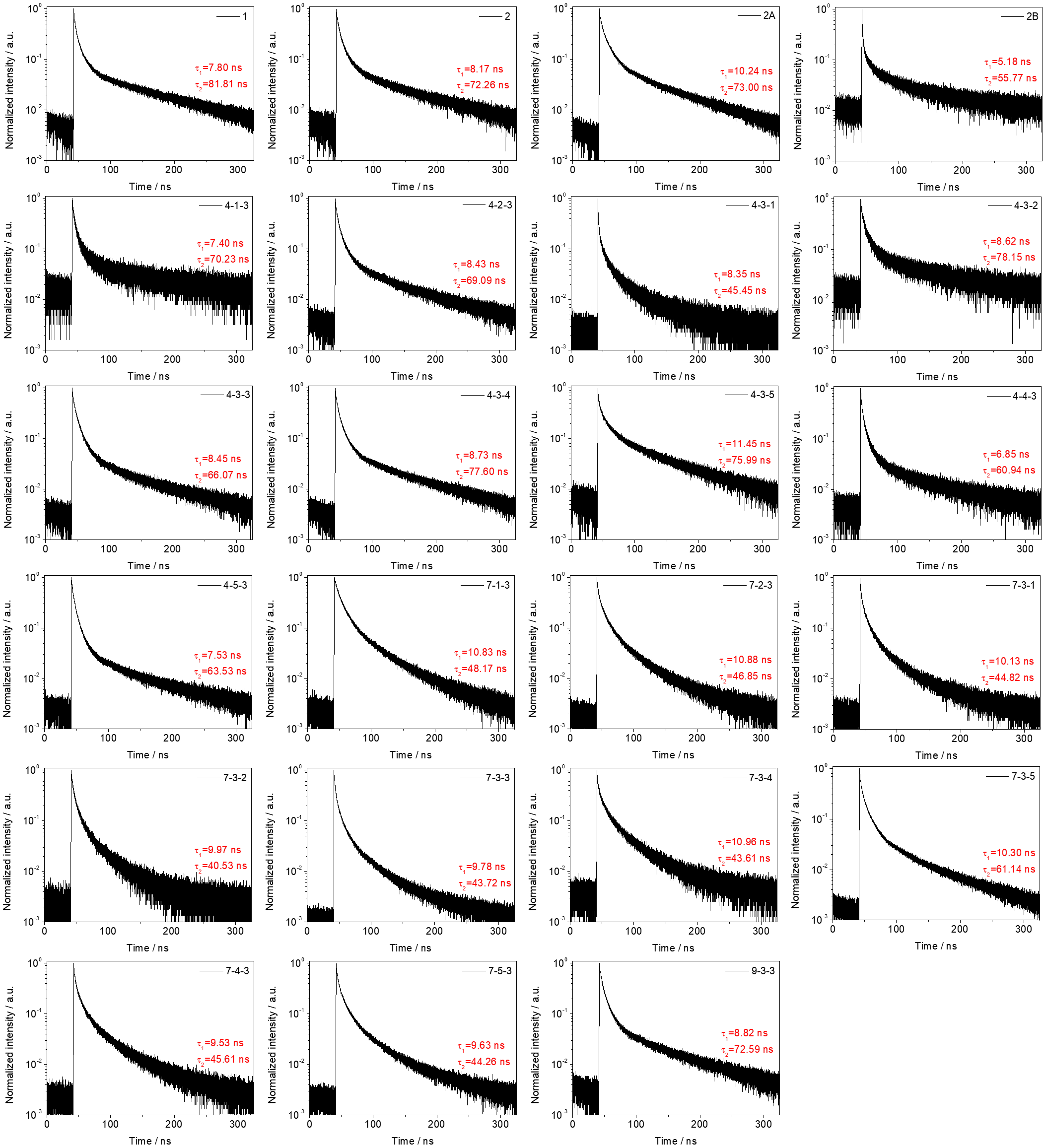


**Figure S12.** Time-resolved photoluminescence spectra for different wafers of CsPbBr_3_ ingot with ZR.


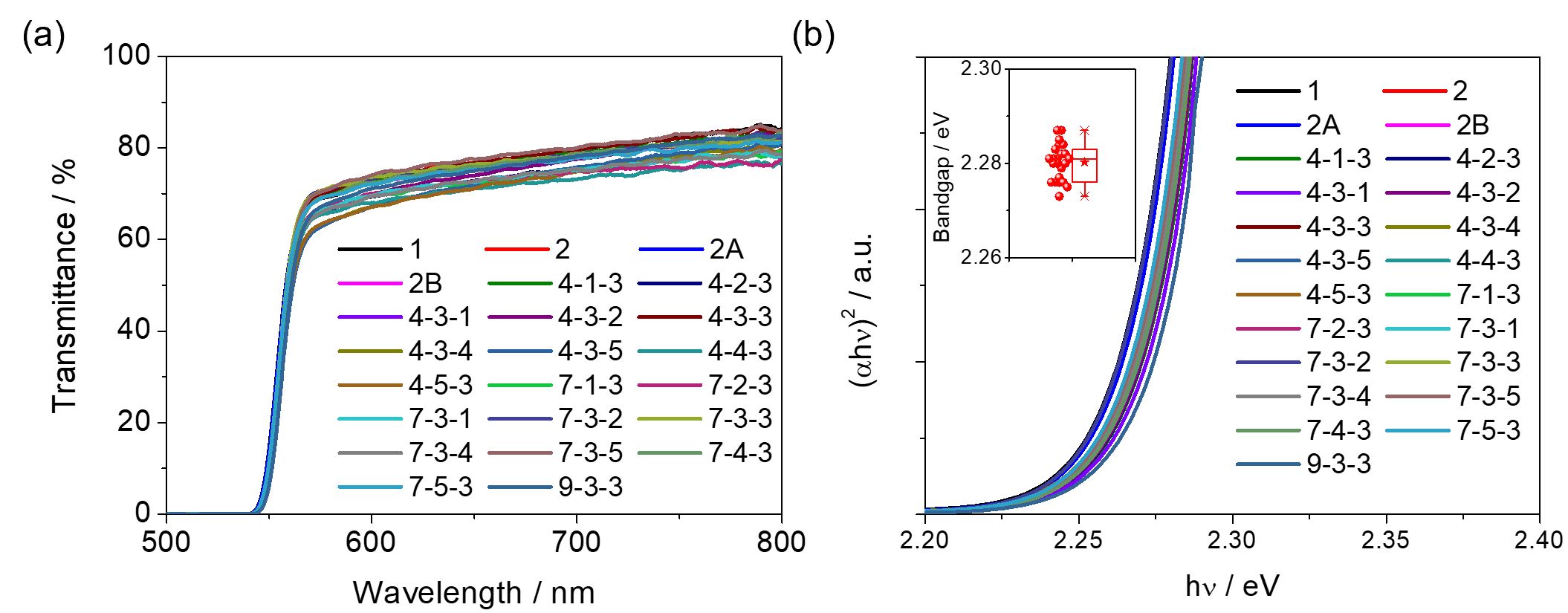


**Figure S13.** (a) UV-Vis-NIR transmission spectra for different wafers of CsPbBr_3_ ingot with ZR. (b) The corresponding bandgaps for different wafers, respectively, the bandgaps are all between 2.27 eV and 2.29 eV.


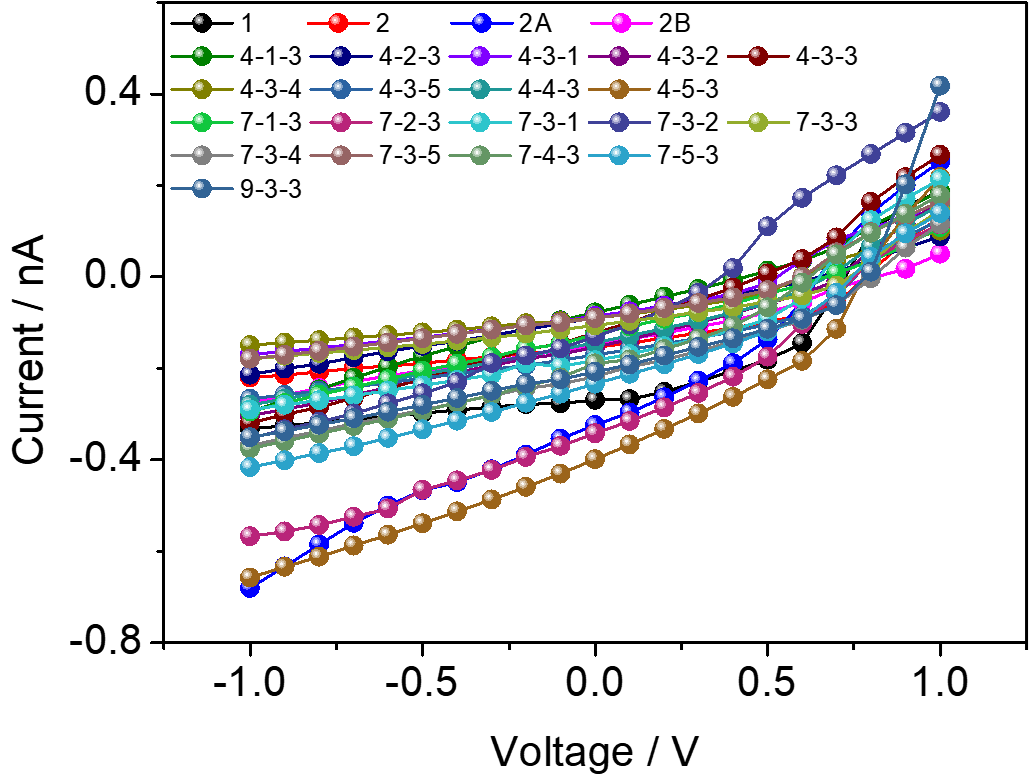


**Figure S14.** Typical dark I-V curves from -1 V to 1 V for different wafers of CsPbBr_3_ ingot with ZR.


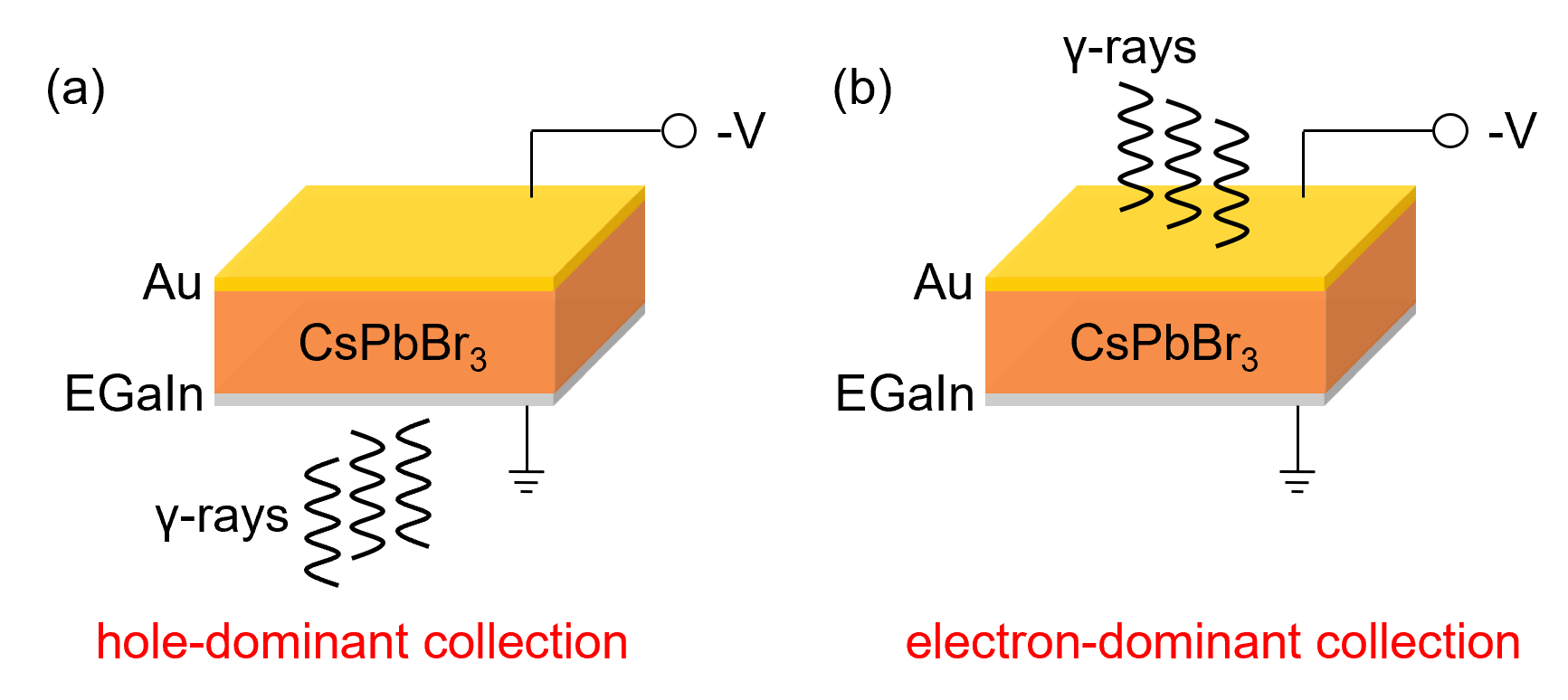


**Figure S15.** Hole-dominant and electron-dominant collection mode by irradiated from anode and cathode using ^241^Am and ^57^Co γ-ray sources, respectively.


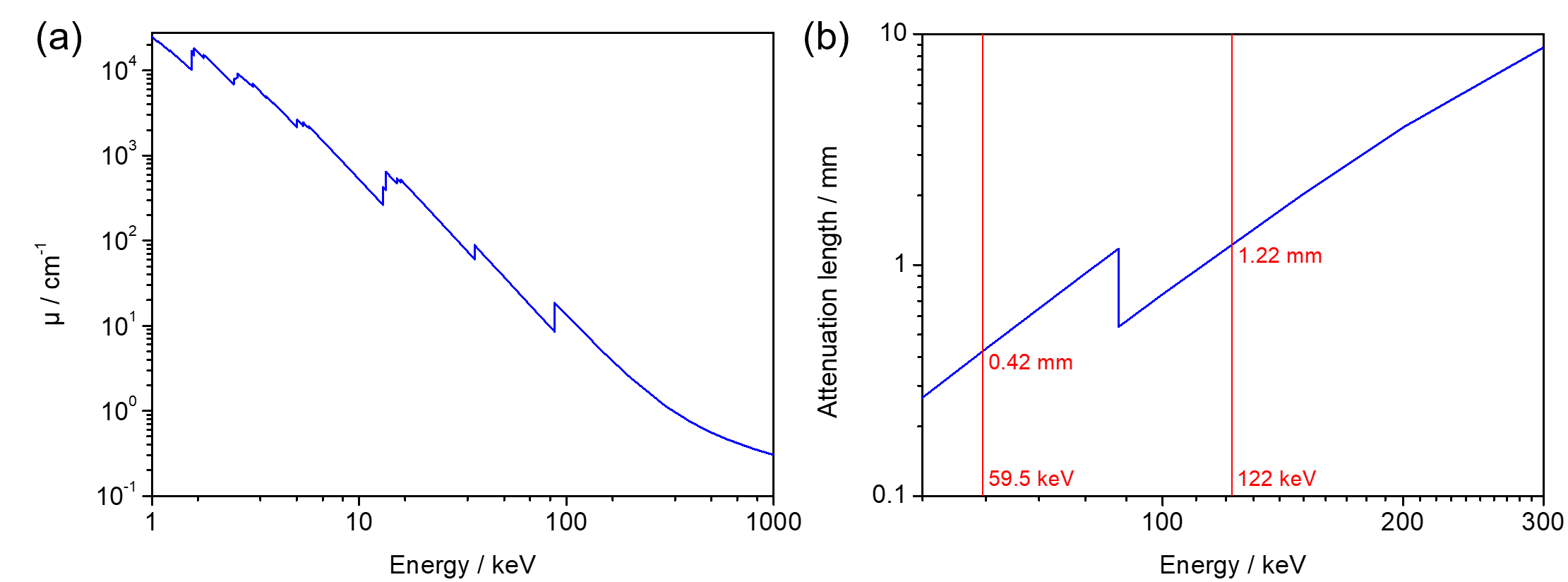


**Figure S16.** The attenuation coefficient and attenuation length of CsPbBr_3_ crystal. For 59.5 keV ^241^Am and 122 keV ^57^Co γ-ray, the total attenuation lengths are 0.42 mm and 1.22 mm, respectively.


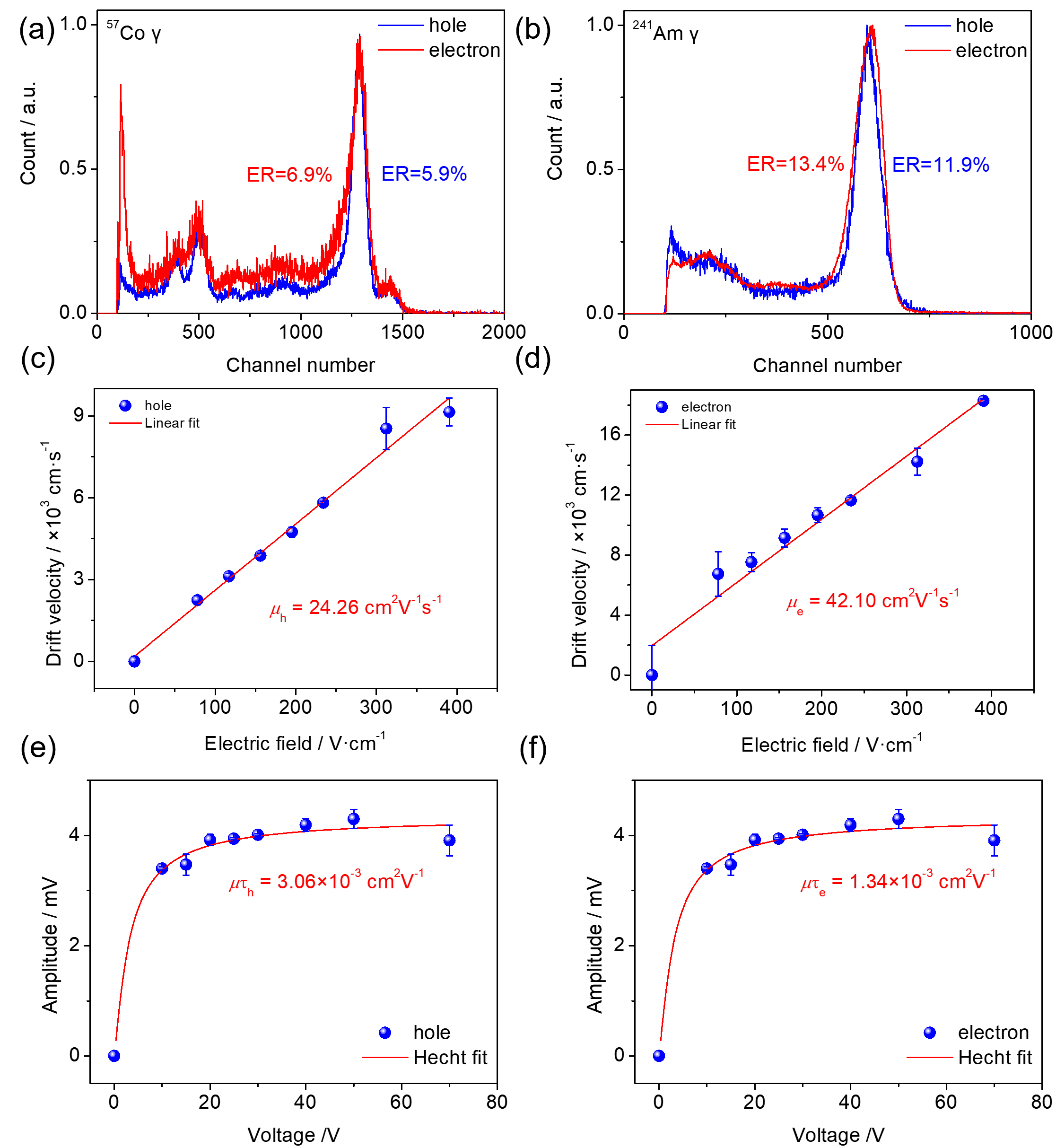


**Figure S17.** The detector performance of wafer 1: (a), (b) ^57^Co and ^241^Am γ-ray spectra obtained by irradiated from anode (hole collection) and cathode (electron collection), respectively. (c), (d) Hole and electron mobilities by linearly fitting the electric field-dependent drift velocity. (e), (f) Hole and electron mobility-lifetime product (*μτ*_h_) according to the Hecht equation.


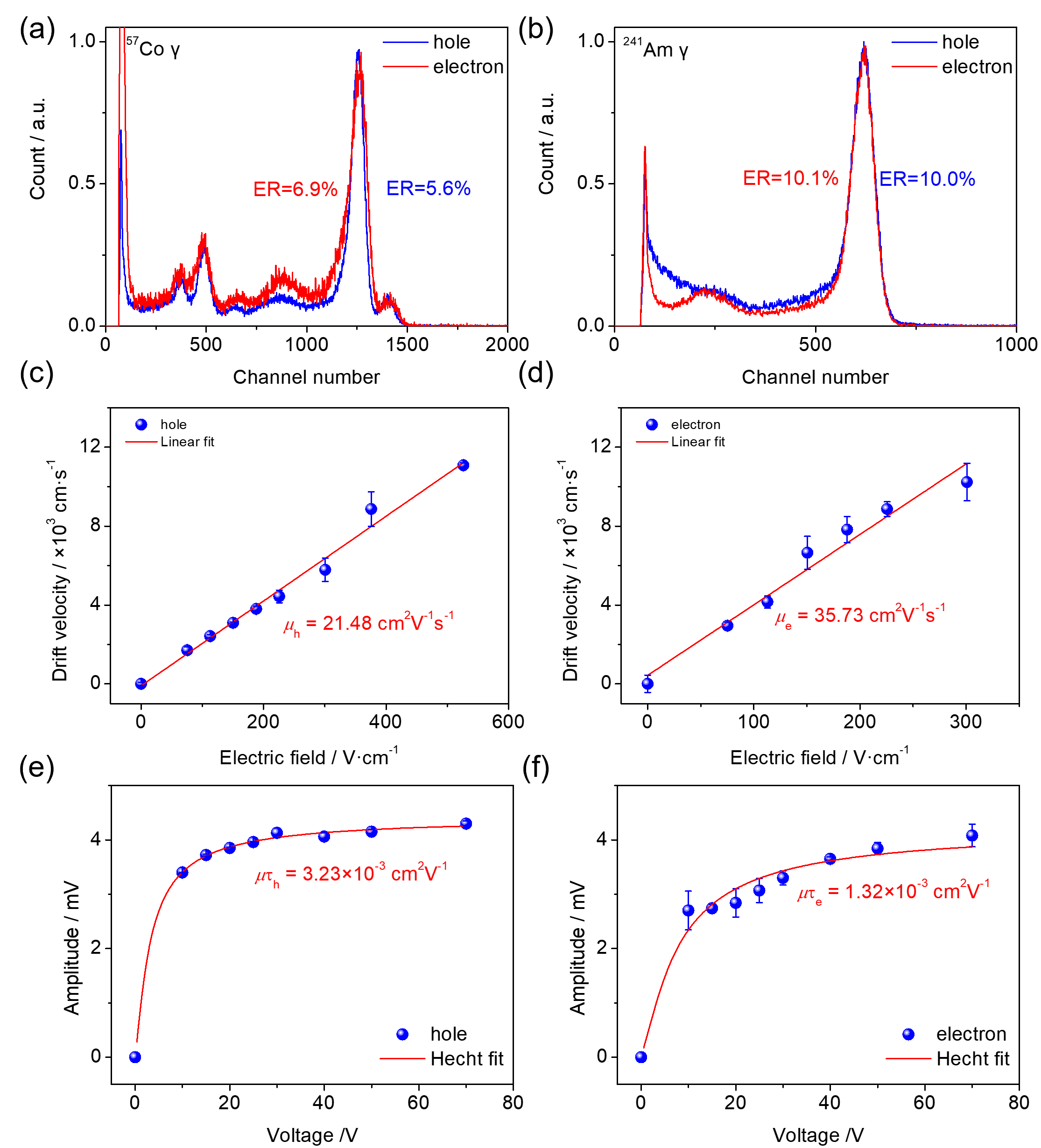


**Figure S18.** The detector performance of wafer 2: (a), (b) ^57^Co and ^241^Am γ-ray spectra obtained by irradiated from anode (hole collection) and cathode (electron collection), respectively. (c), (d) Hole and electron mobilities by linearly fitting the electric field-dependent drift velocity. (e), (f) Hole and electron mobility-lifetime product (*μτ*_h_) according to the Hecht equation.


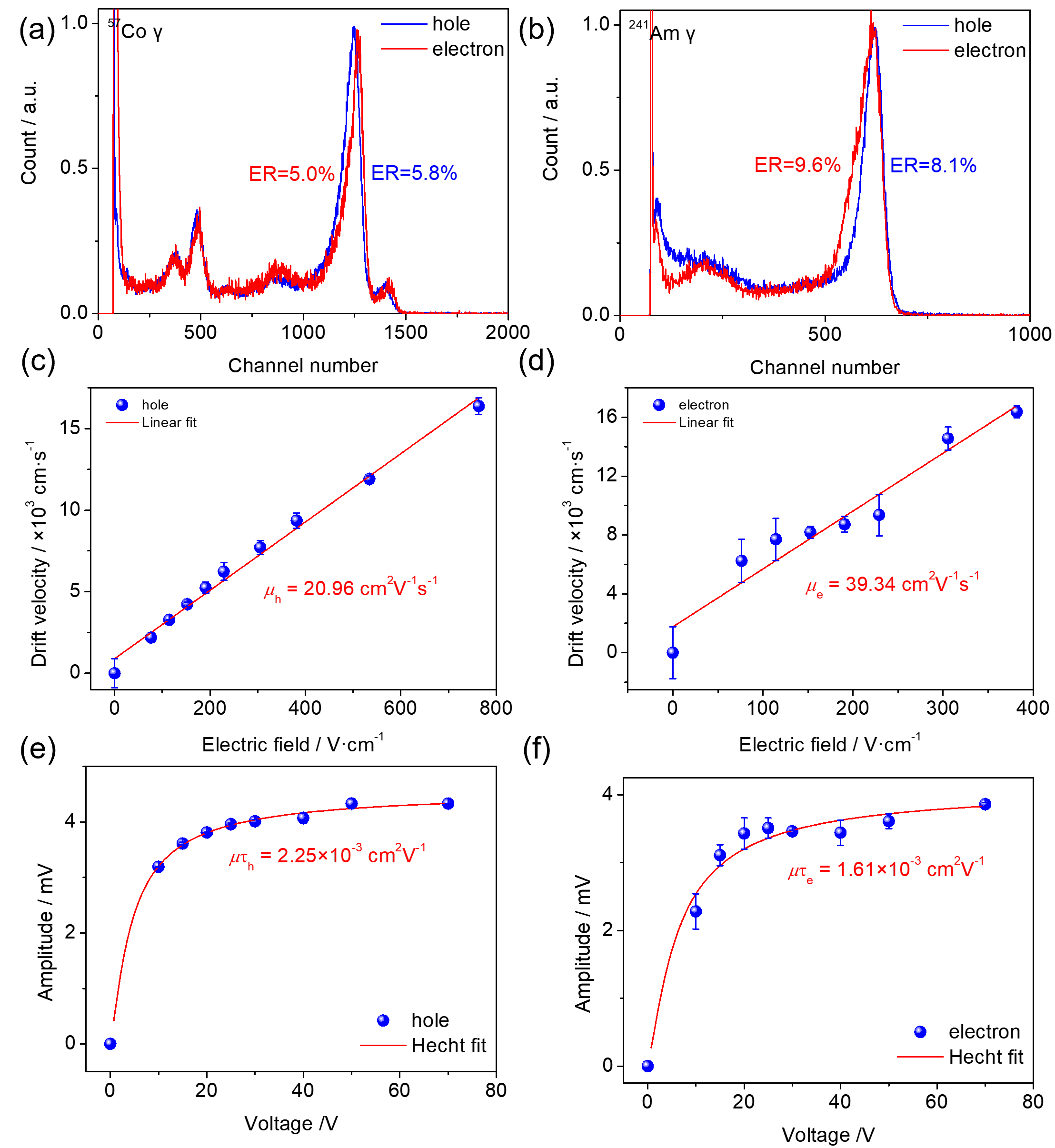


**Figure S19.** The detector performance of wafer 2A: (a), (b) ^57^Co and ^241^Am γ-ray spectra obtained by irradiated from anode (hole collection) and cathode (electron collection), respectively. (c), (d) Hole and electron mobilities by linearly fitting the electric field-dependent drift velocity. (e), (f) Hole and electron mobility-lifetime product (*μτ*_h_) according to the Hecht equation.


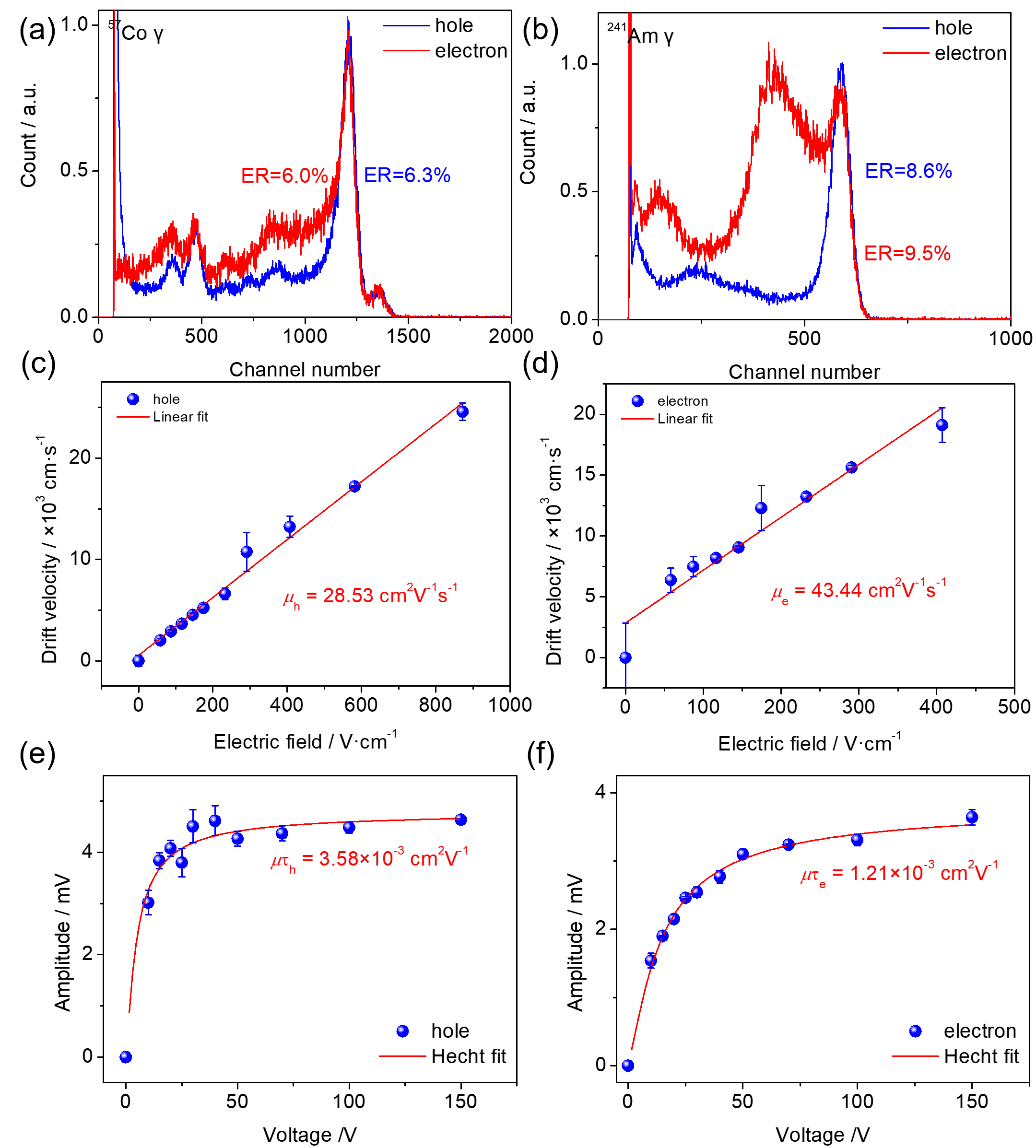


**Figure S20.** The detector performance of wafer 2B: (a), (b) ^57^Co and ^241^Am γ-ray spectra obtained by irradiated from anode (hole collection) and cathode (electron collection), respectively. (c), (d) Hole and electron mobilities by linearly fitting the electric field-dependent drift velocity. (e), (f) Hole and electron mobility-lifetime product (*μτ*_h_) according to the Hecht equation.


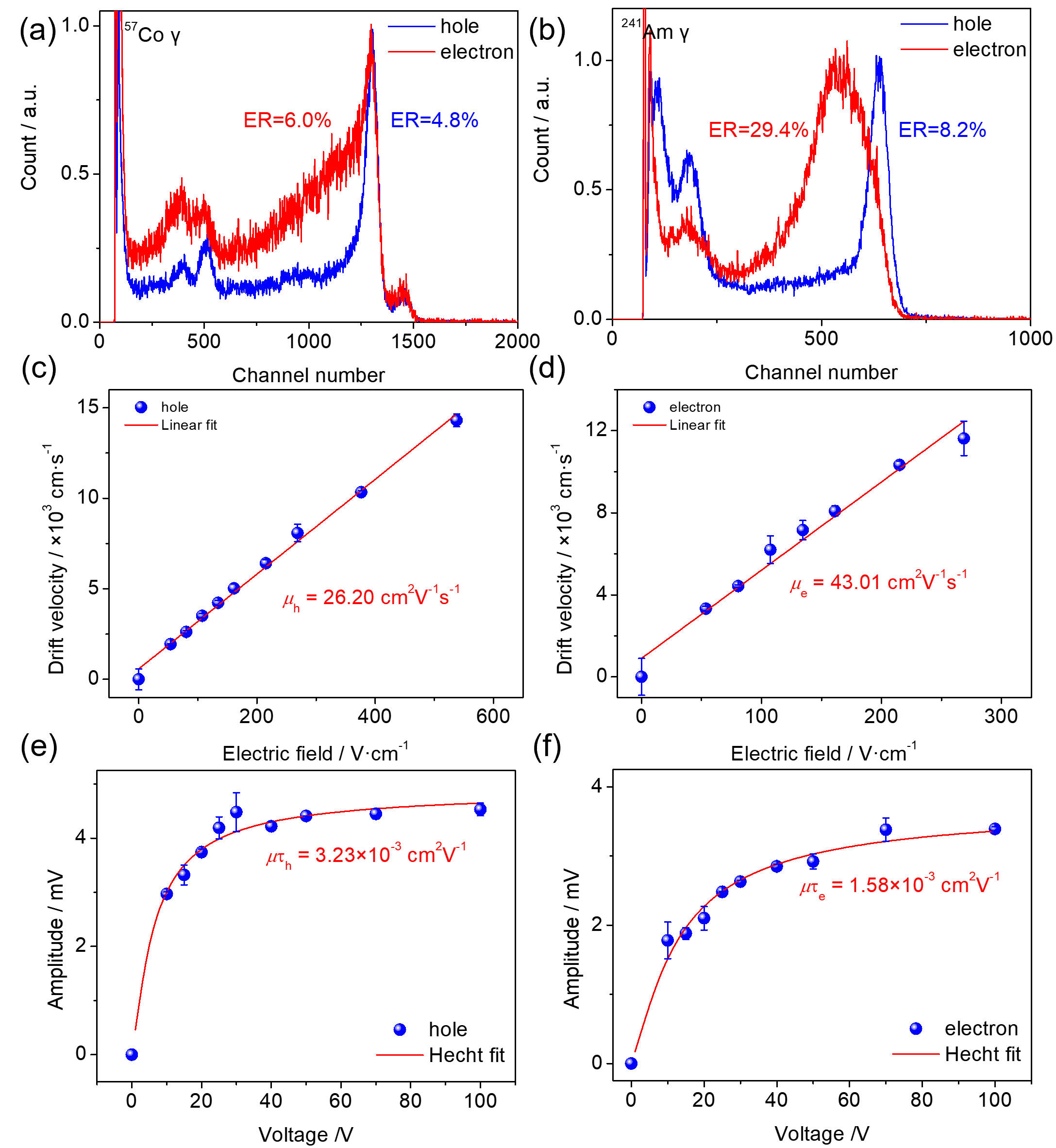


**Figure S21.** The detector performance of wafer 4-1-3: (a), (b) ^57^Co and ^241^Am γ-ray spectra obtained by irradiated from anode (hole collection) and cathode (electron collection), respectively. (c), (d) Hole and electron mobilities by linearly fitting the electric field-dependent drift velocity. (e), (f) Hole and electron mobility-lifetime product (*μτ*_h_) according to the Hecht equation.


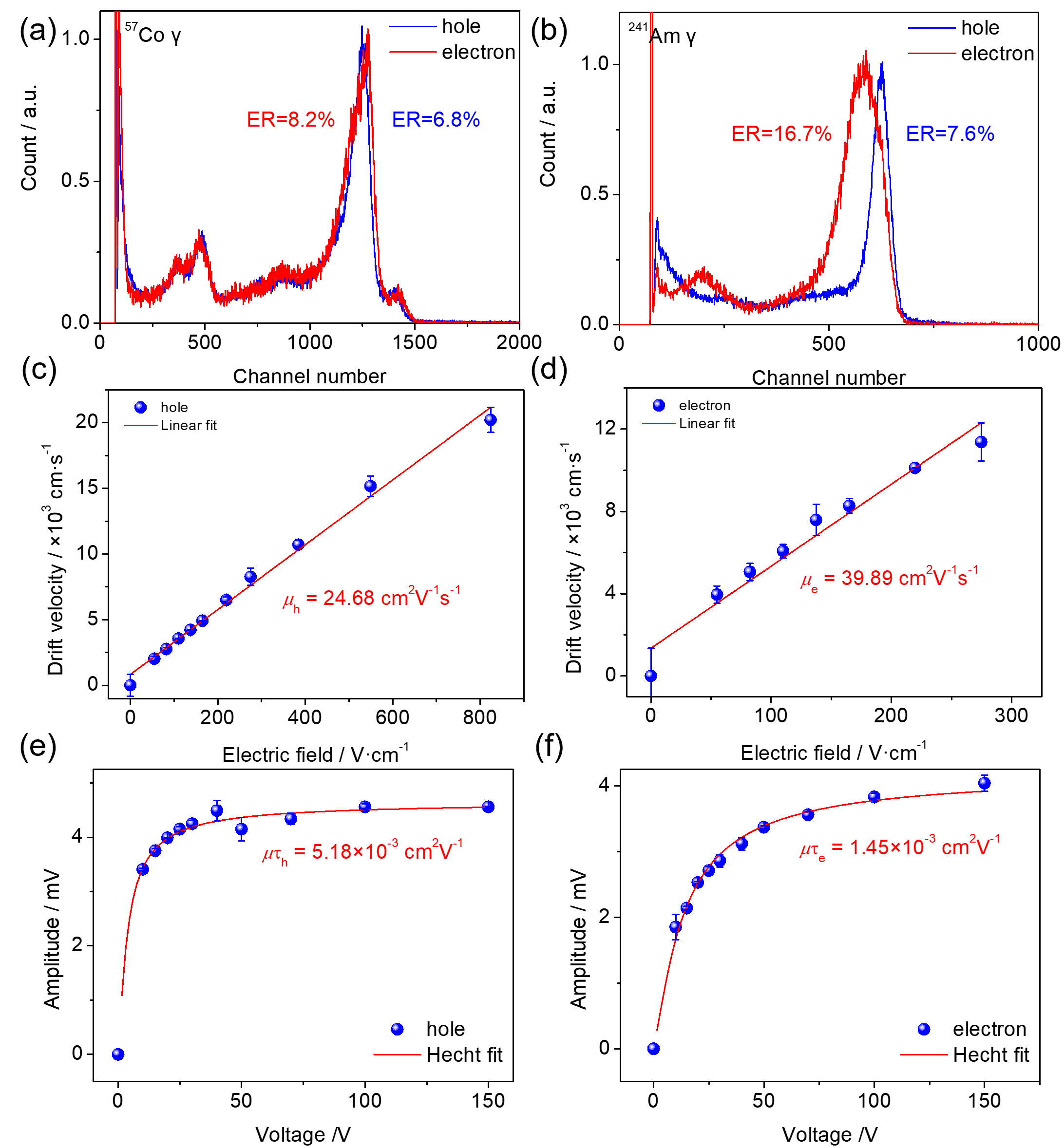


**Figure S22.** The detector performance of wafer 4-2-3: (a), (b) ^57^Co and ^241^Am γ-ray spectra obtained by irradiated from anode (hole collection) and cathode (electron collection), respectively. (c), (d) Hole and electron mobilities by linearly fitting the electric field-dependent drift velocity. (e), (f) Hole and electron mobility-lifetime product (*μτ*_h_) according to the Hecht equation.

**
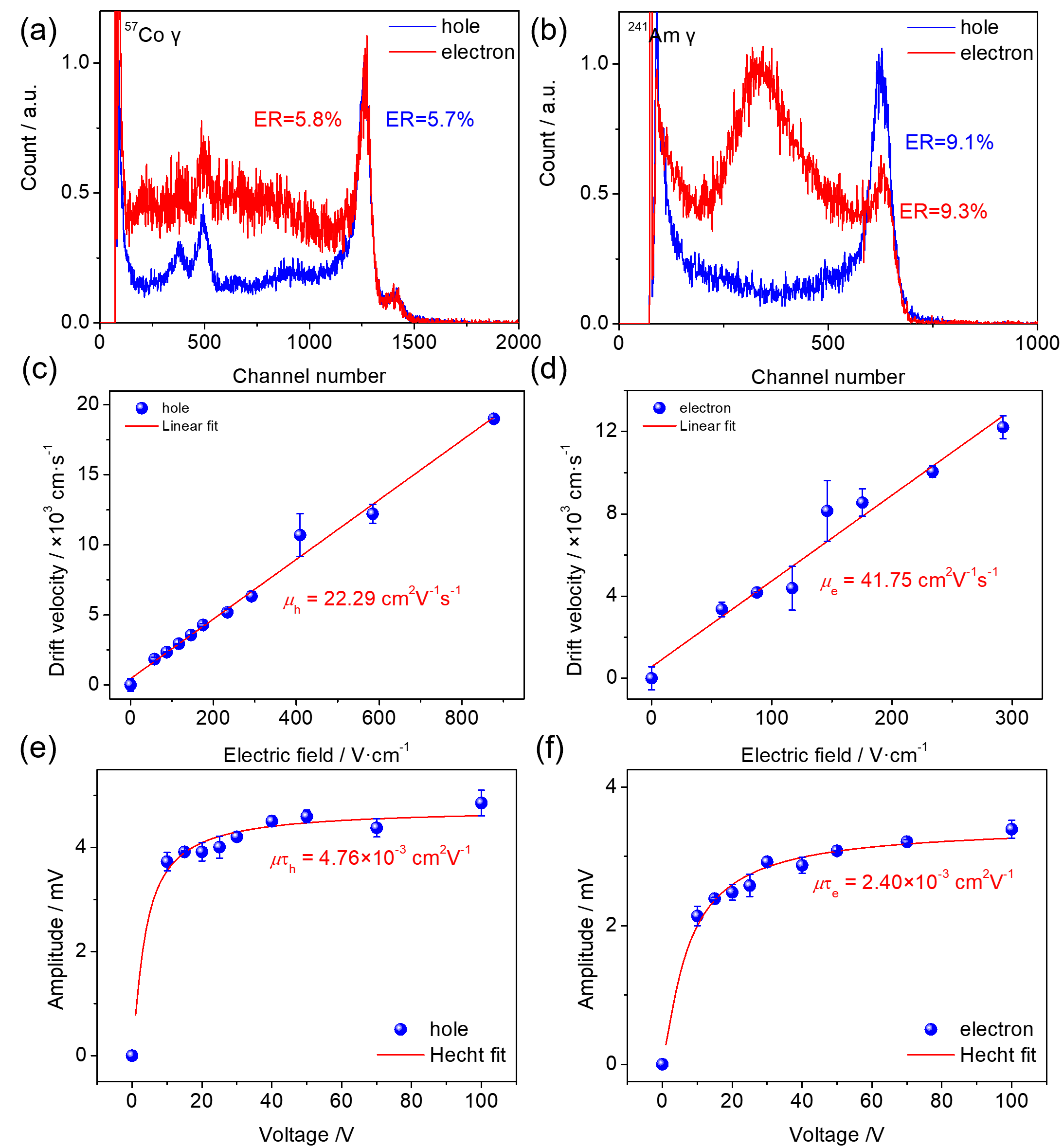
**

**Figure S23.** The detector performance of wafer 4-3-1: (a), (b) ^57^Co and ^241^Am γ-ray spectra obtained by irradiated from anode (hole collection) and cathode (electron collection), respectively. (c), (d) Hole and electron mobilities by linearly fitting the electric field-dependent drift velocity. (e), (f) Hole and electron mobility-lifetime product (*μτ*_h_) according to the Hecht equation.


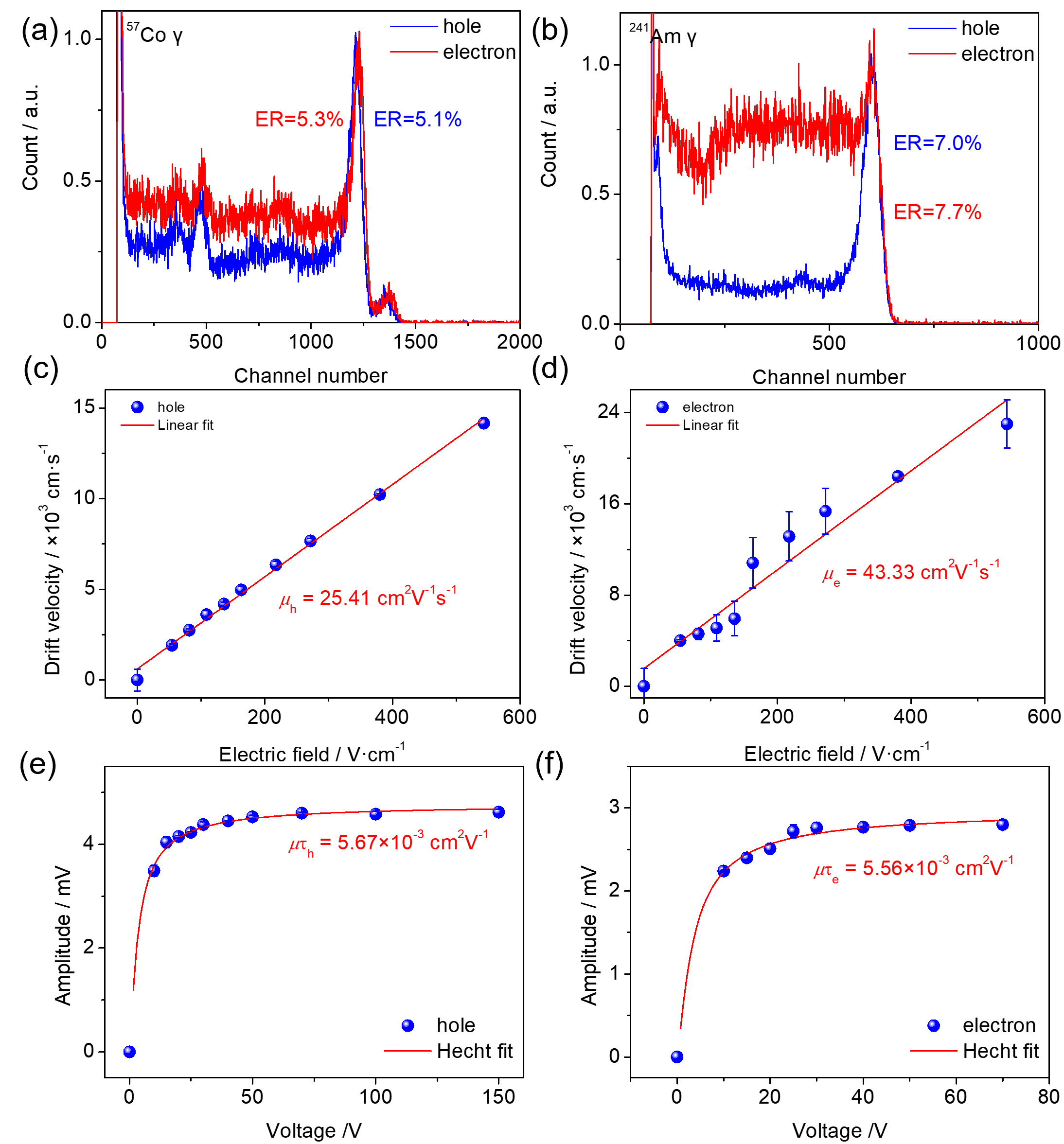


**Figure S24.** The detector performance of wafer 4-3-2: (a), (b) ^57^Co and ^241^Am γ-ray spectra obtained by irradiated from anode (hole collection) and cathode (electron collection), respectively. (c), (d) Hole and electron mobilities by linearly fitting the electric field-dependent drift velocity. (e), (f) Hole and electron mobility-lifetime product (*μτ*_h_) according to the Hecht equation.

**
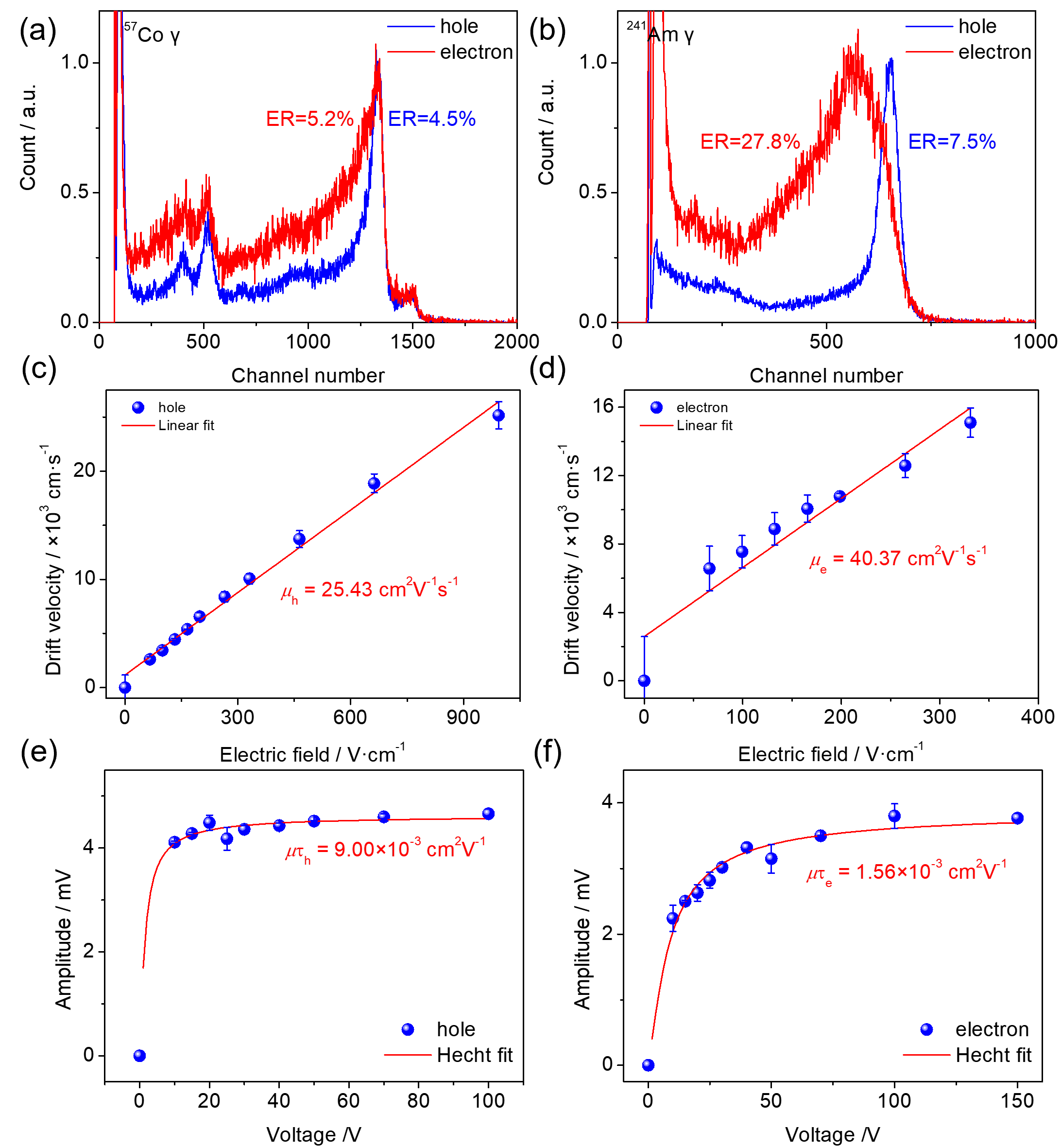
**

**Figure S25.** The detector performance of wafer 4-3-3: (a), (b) ^57^Co and ^241^Am γ-ray spectra obtained by irradiated from anode (hole collection) and cathode (electron collection), respectively. (c), (d) Hole and electron mobilities by linearly fitting the electric field-dependent drift velocity. (e), (f) Hole and electron mobility-lifetime product (*μτ*_h_) according to the Hecht equation.


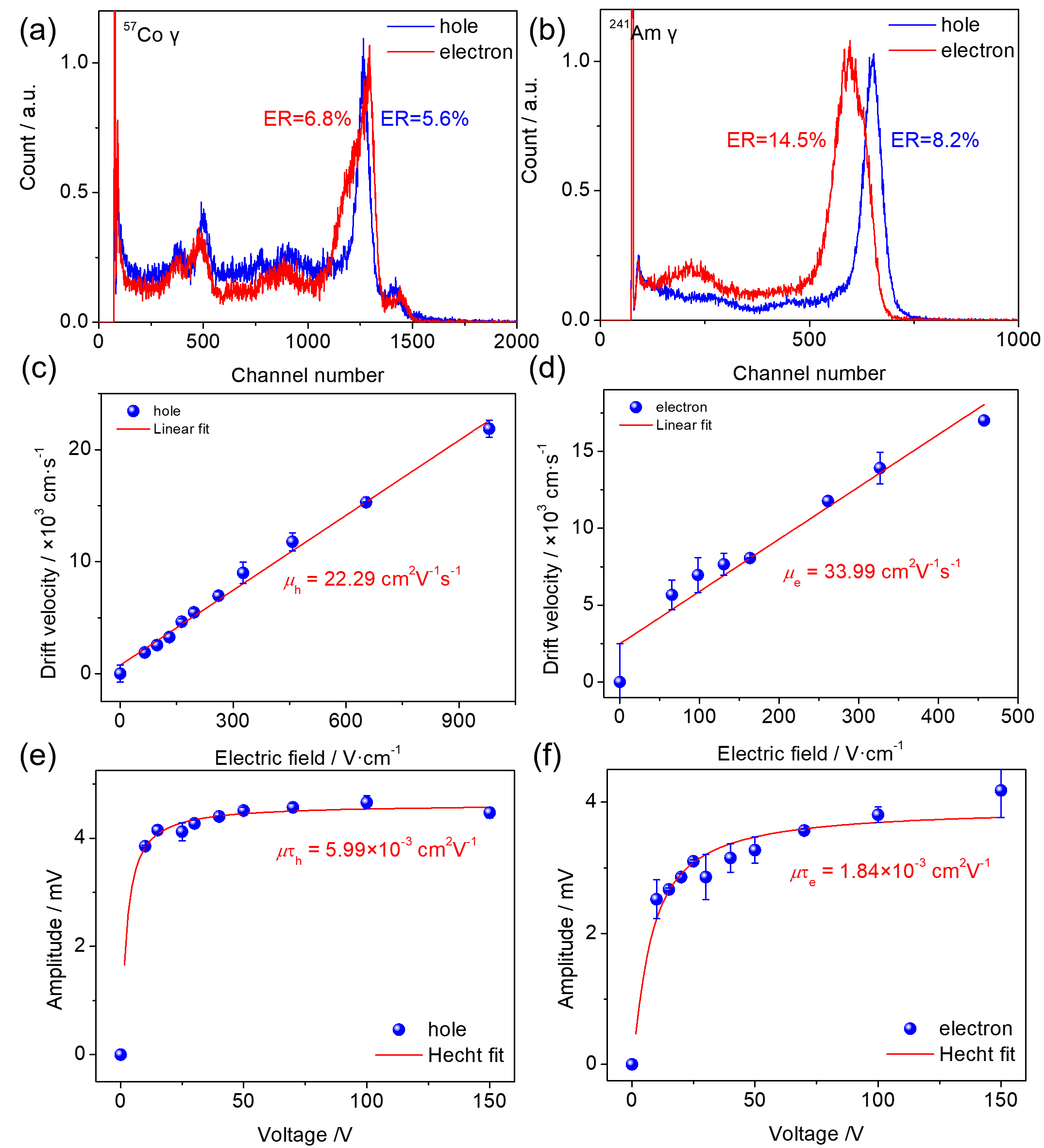


**Figure S26.** The detector performance of wafer 4-3-4: (a), (b) ^57^Co and ^241^Am γ-ray spectra obtained by irradiated from anode (hole collection) and cathode (electron collection), respectively. (c), (d) Hole and electron mobilities by linearly fitting the electric field-dependent drift velocity. (e), (f) Hole and electron mobility-lifetime product (*μτ*_h_) according to the Hecht equation.

**
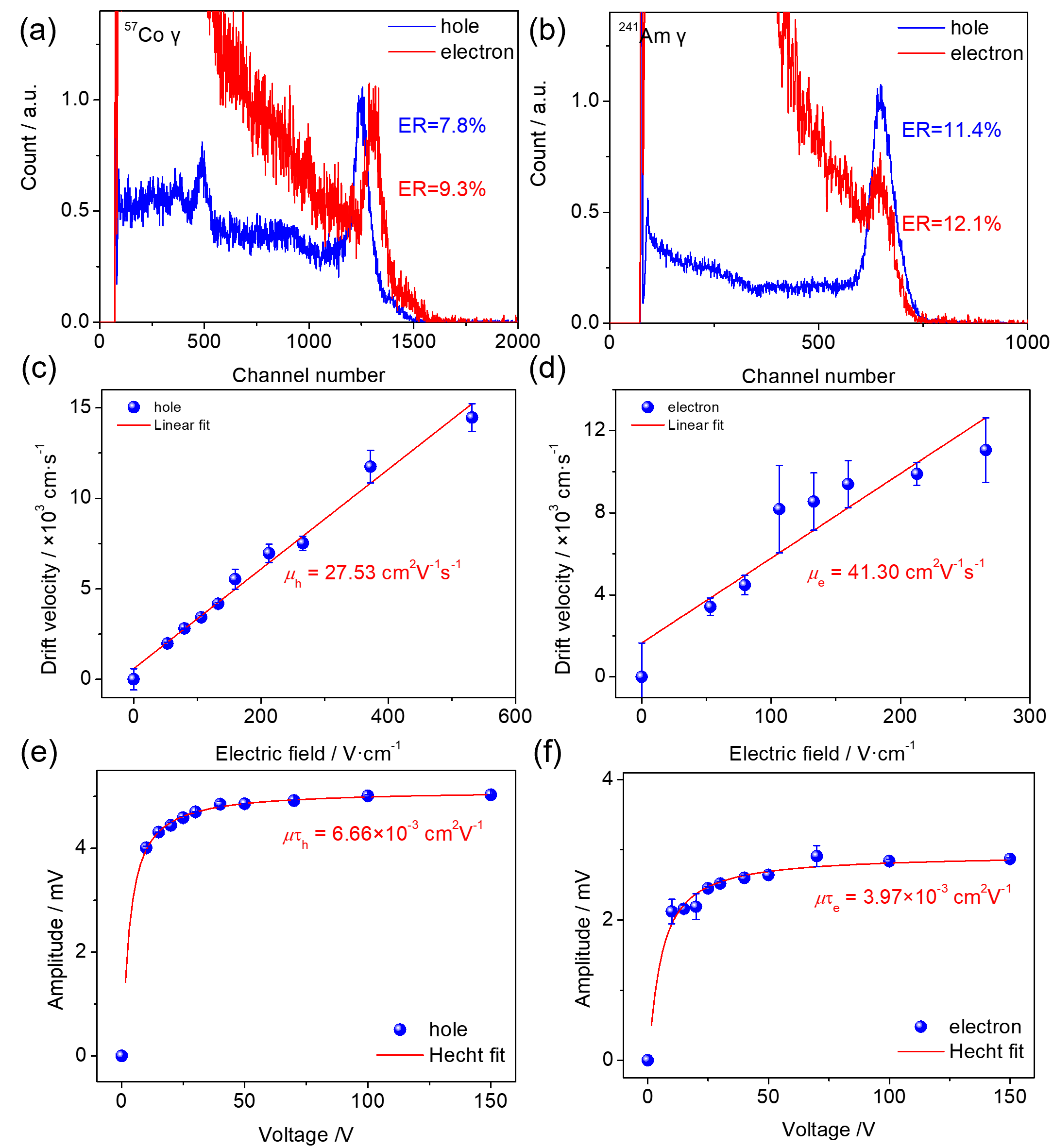
**

**Figure S27.** The detector performance of wafer 4-3-5: (a), (b) ^57^Co and ^241^Am γ-ray spectra obtained by irradiated from anode (hole collection) and cathode (electron collection), respectively. (c), (d) Hole and electron mobilities by linearly fitting the electric field-dependent drift velocity. (e), (f) Hole and electron mobility-lifetime product (*μτ*_h_) according to the Hecht equation.


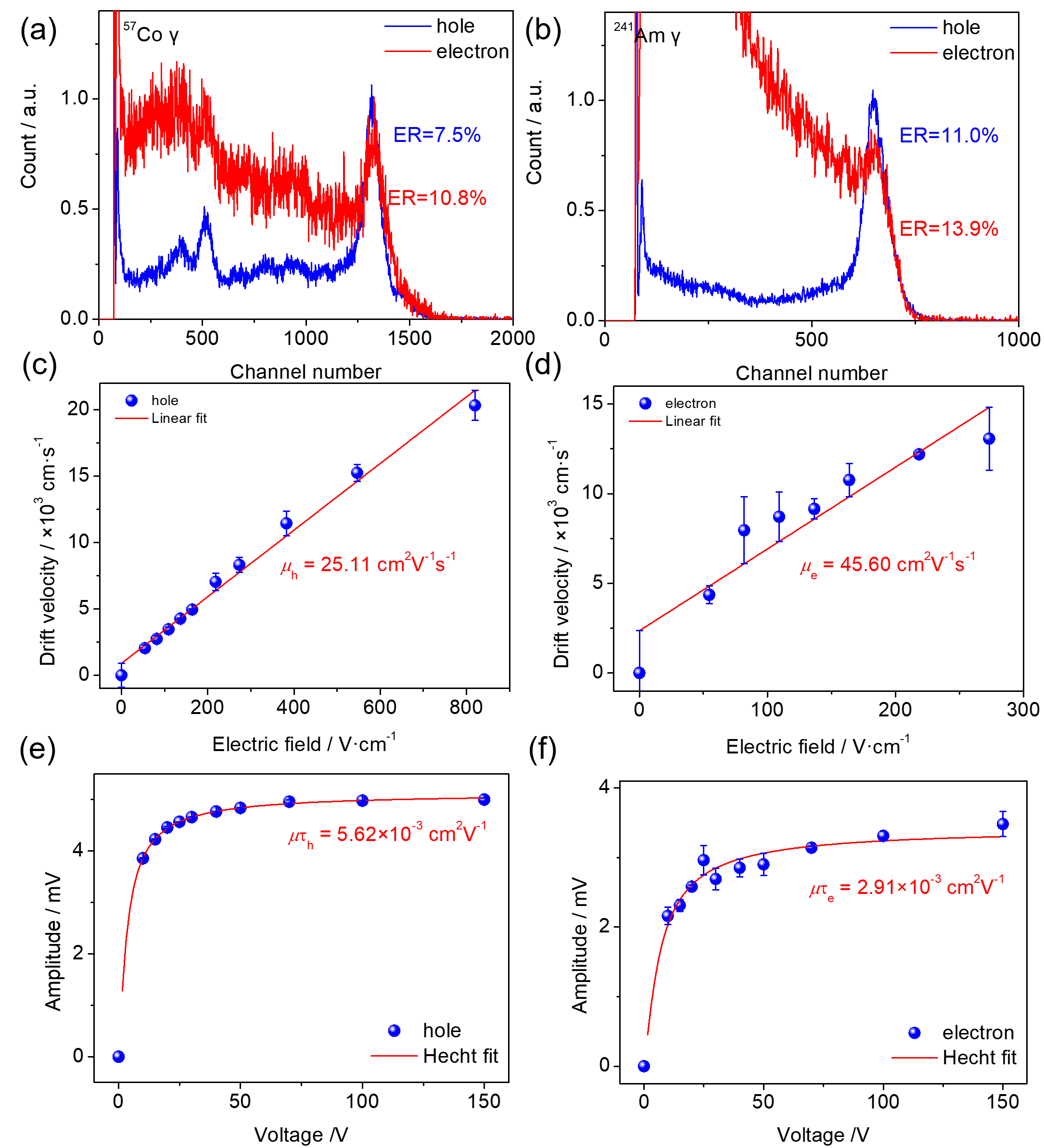


**Figure S28.** The detector performance of wafer 4-4-3: (a), (b) ^57^Co and ^241^Am γ-ray spectra obtained by irradiated from anode (hole collection) and cathode (electron collection), respectively. (c), (d) Hole and electron mobilities by linearly fitting the electric field-dependent drift velocity. (e), (f) Hole and electron mobility-lifetime product (*μτ*_h_) according to the Hecht equation.

**
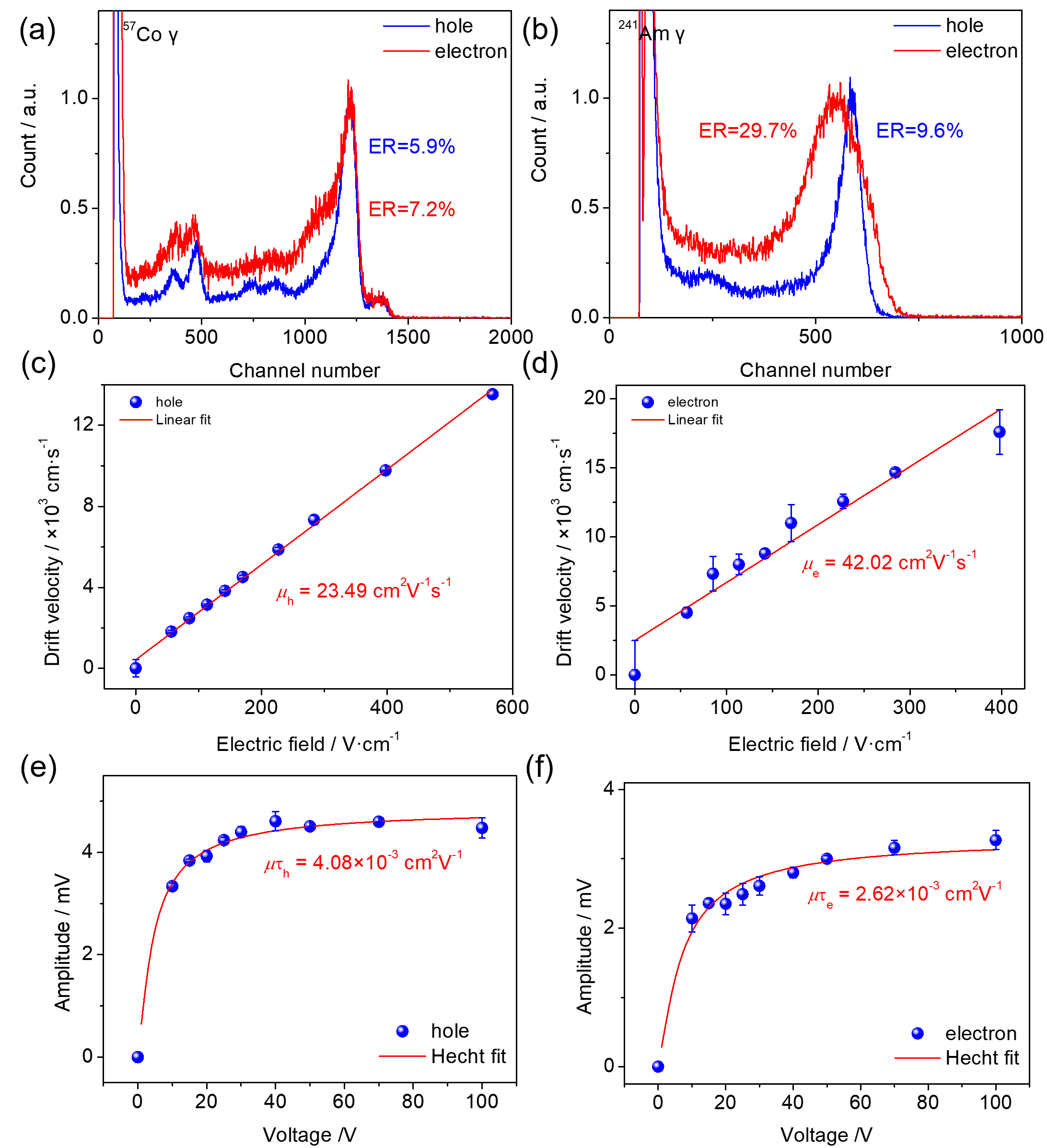
**

**Figure S29.** The detector performance of wafer 4-5-3: (a), (b) ^57^Co and ^241^Am γ-ray spectra obtained by irradiated from anode (hole collection) and cathode (electron collection), respectively. (c), (d) Hole and electron mobilities by linearly fitting the electric field-dependent drift velocity. (e), (f) Hole and electron mobility-lifetime product (*μτ*_h_) according to the Hecht equation.


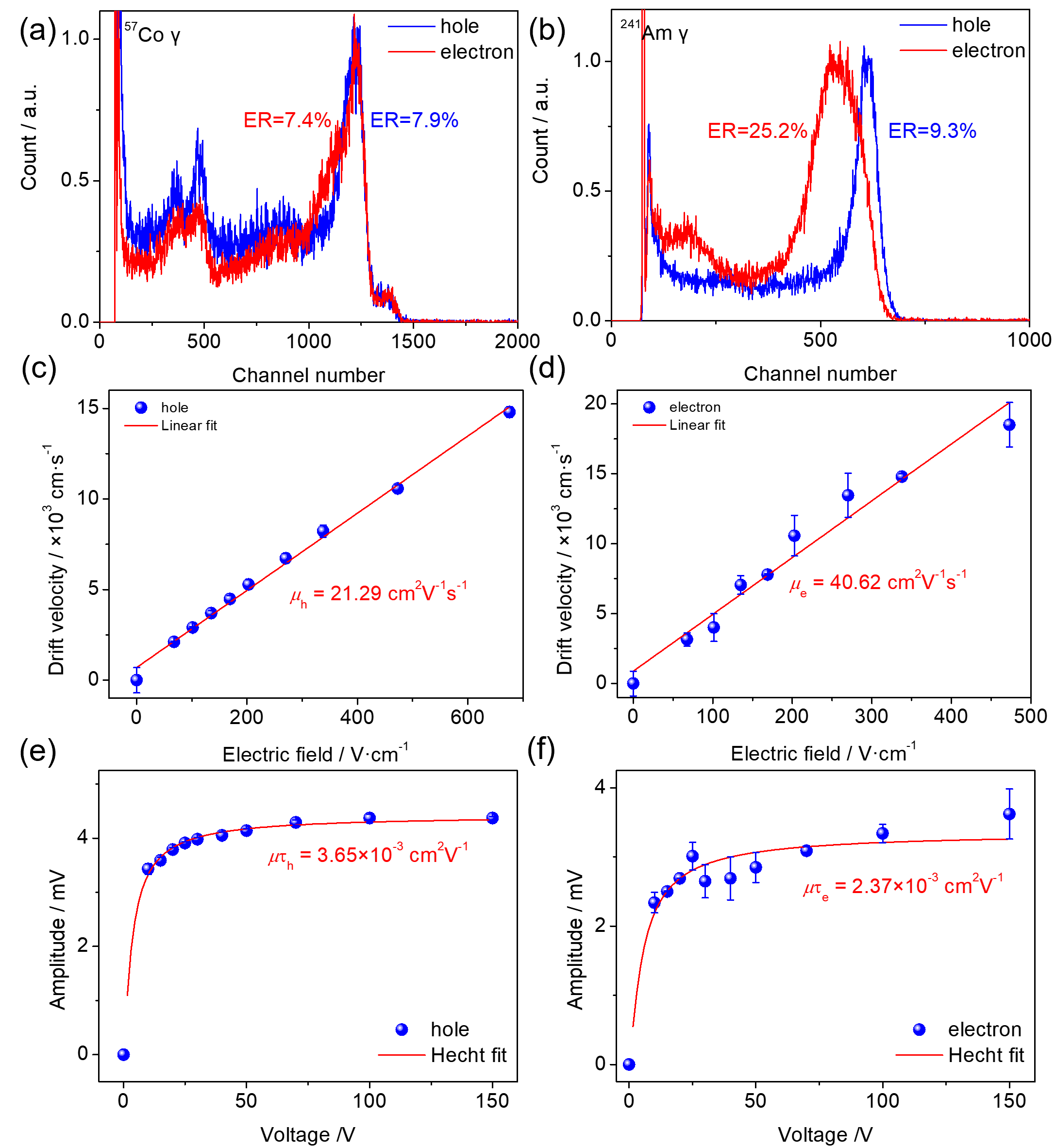


**Figure S30.** The detector performance of wafer 7-1-3: (a), (b) ^57^Co and ^241^Am γ-ray spectra obtained by irradiated from anode (hole collection) and cathode (electron collection), respectively. (c), (d) Hole and electron mobilities by linearly fitting the electric field-dependent drift velocity. (e), (f) Hole and electron mobility-lifetime product (*μτ*_h_) according to the Hecht equation.

**
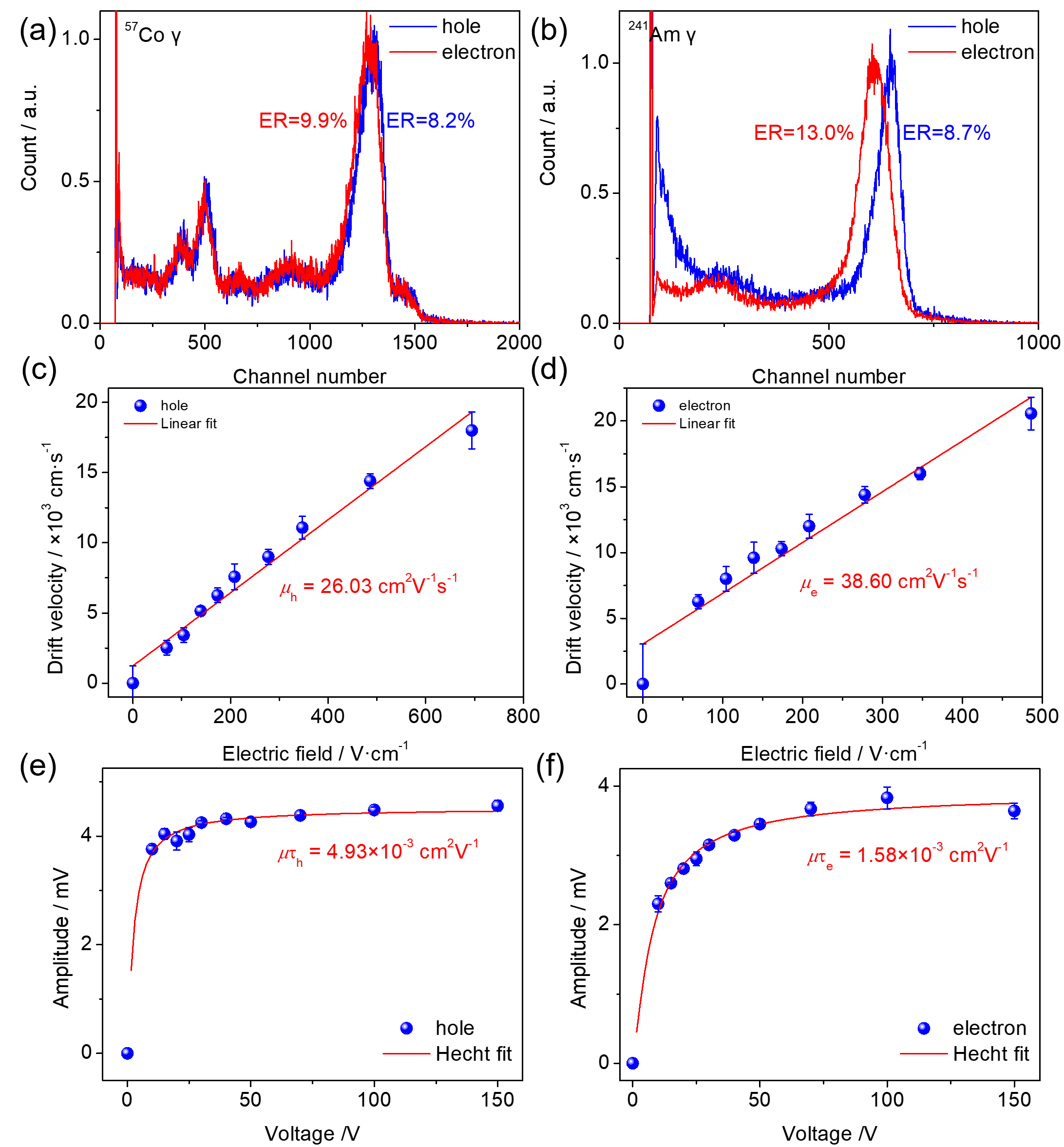
**

**Figure S31.** The detector performance of wafer 7-2-3: (a), (b) ^57^Co and ^241^Am γ-ray spectra obtained by irradiated from anode (hole collection) and cathode (electron collection), respectively. (c), (d) Hole and electron mobilities by linearly fitting the electric field-dependent drift velocity. (e), (f) Hole and electron mobility-lifetime product (*μτ*_h_) according to the Hecht equation.


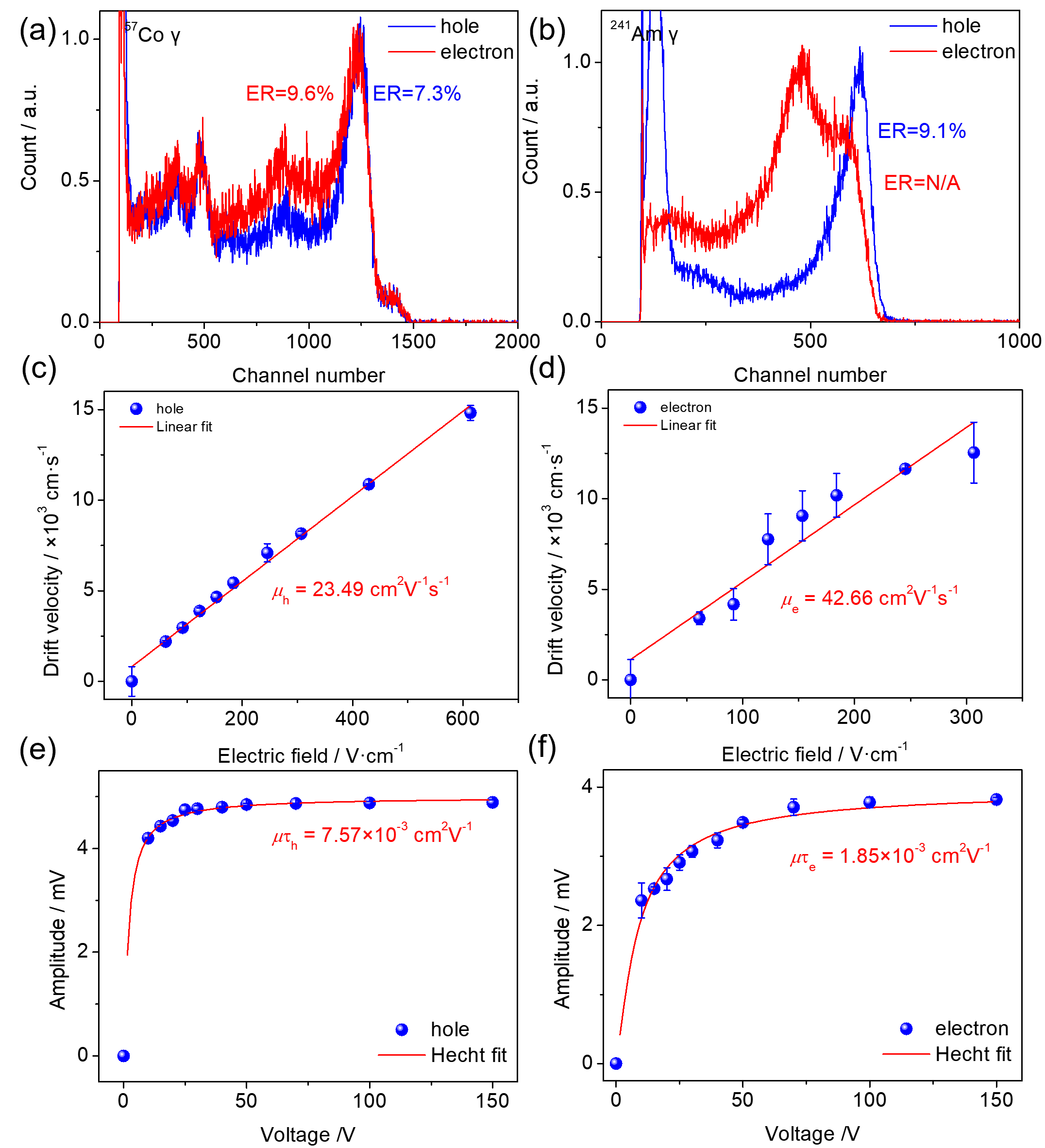


**Figure S32.** The detector performance of wafer 7-3-1: (a), (b) ^57^Co and ^241^Am γ-ray spectra obtained by irradiated from anode (hole collection) and cathode (electron collection), respectively. (c), (d) Hole and electron mobilities by linearly fitting the electric field-dependent drift velocity. (e), (f) Hole and electron mobility-lifetime product (*μτ*_h_) according to the Hecht equation.

**
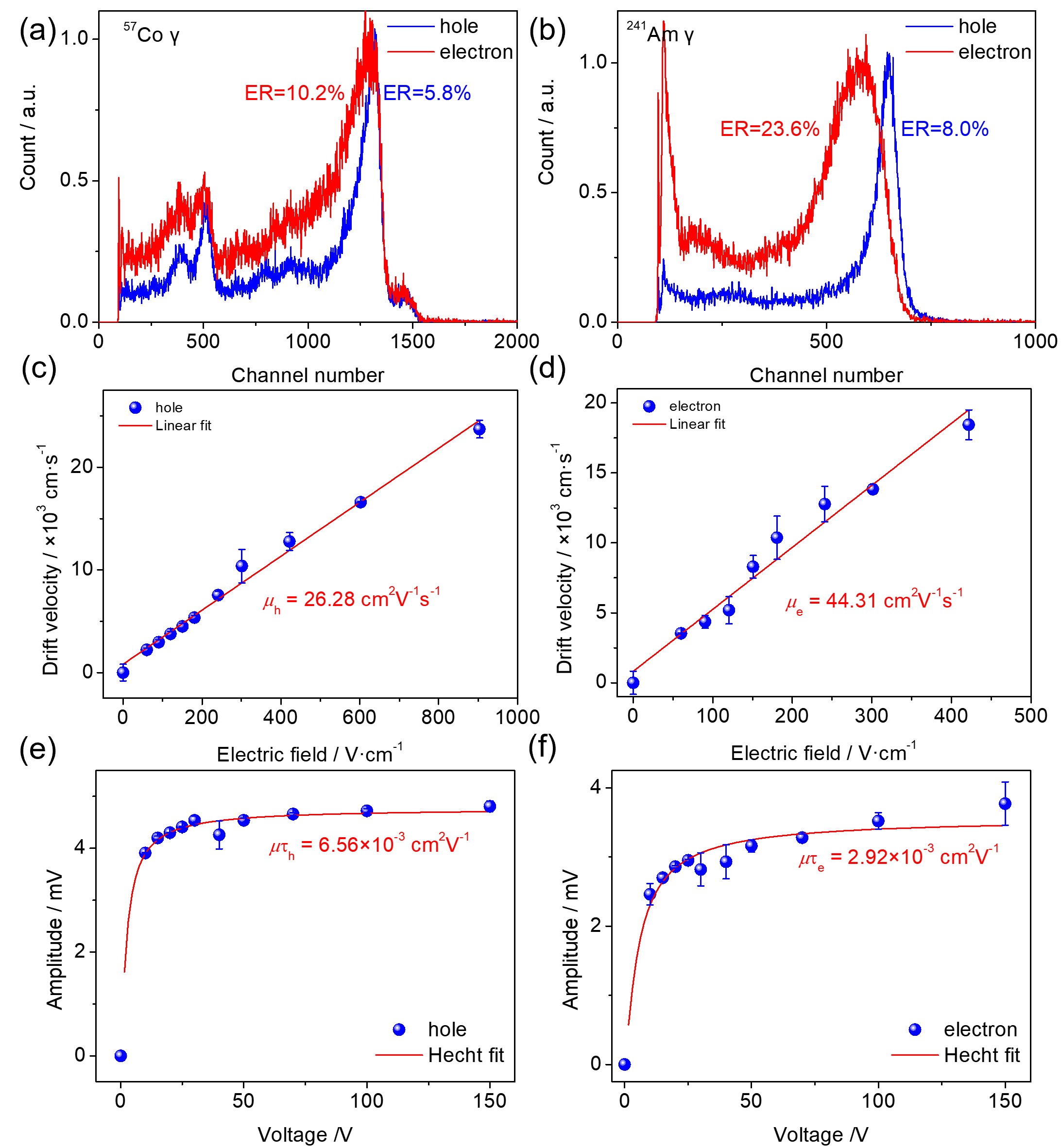
**

**Figure S33.** The detector performance of wafer 7-3-2: (a), (b) ^57^Co and ^241^Am γ-ray spectra obtained by irradiated from anode (hole collection) and cathode (electron collection), respectively. (c), (d) Hole and electron mobilities by linearly fitting the electric field-dependent drift velocity. (e), (f) Hole and electron mobility-lifetime product (*μτ*_h_) according to the Hecht equation.


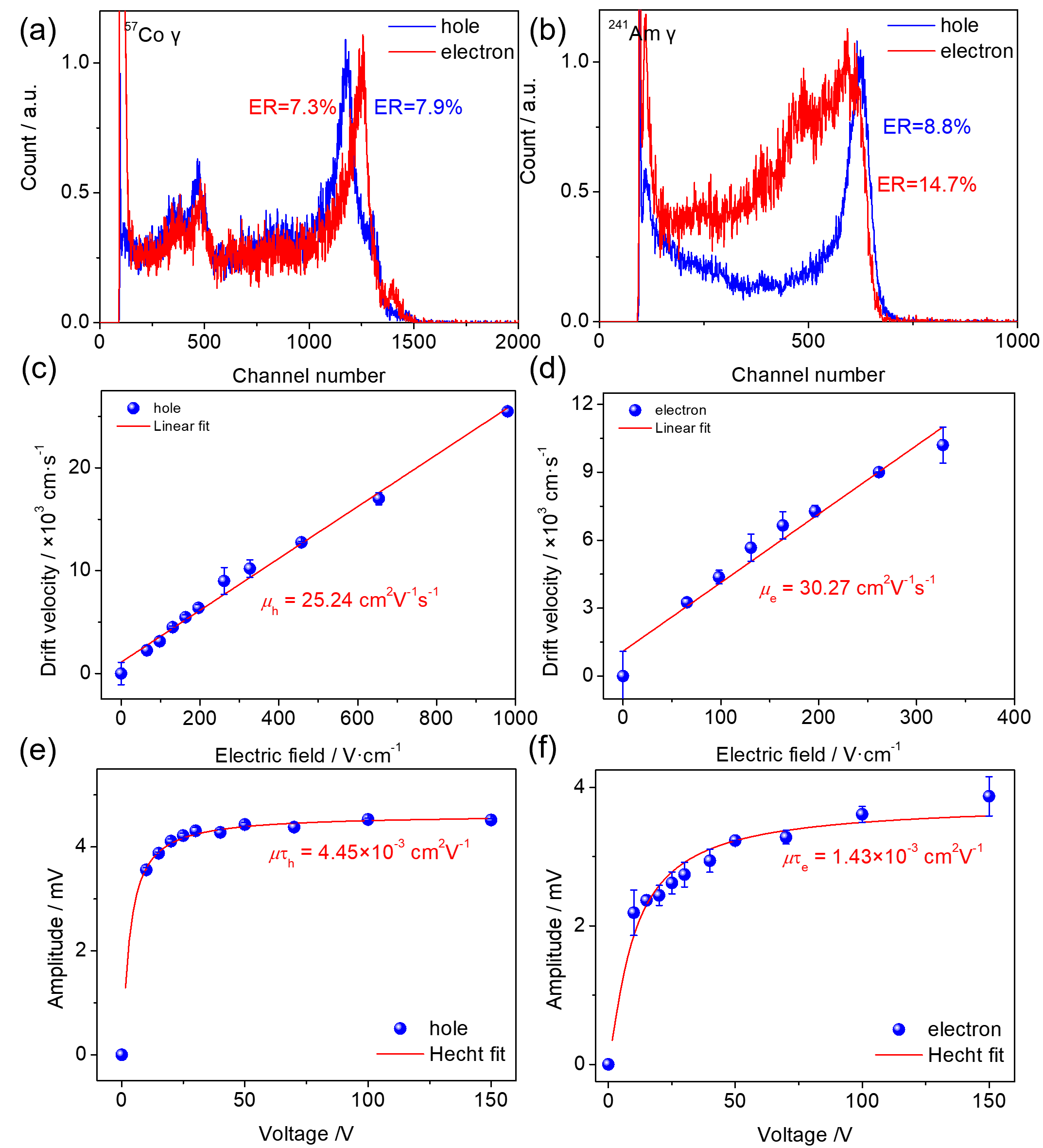


**Figure S34.** The detector performance of wafer 7-3-3: (a), (b) ^57^Co and ^241^Am γ-ray spectra obtained by irradiated from anode (hole collection) and cathode (electron collection), respectively. (c), (d) Hole and electron mobilities by linearly fitting the electric field-dependent drift velocity. (e), (f) Hole and electron mobility-lifetime product (*μτ*_h_) according to the Hecht equation.

**
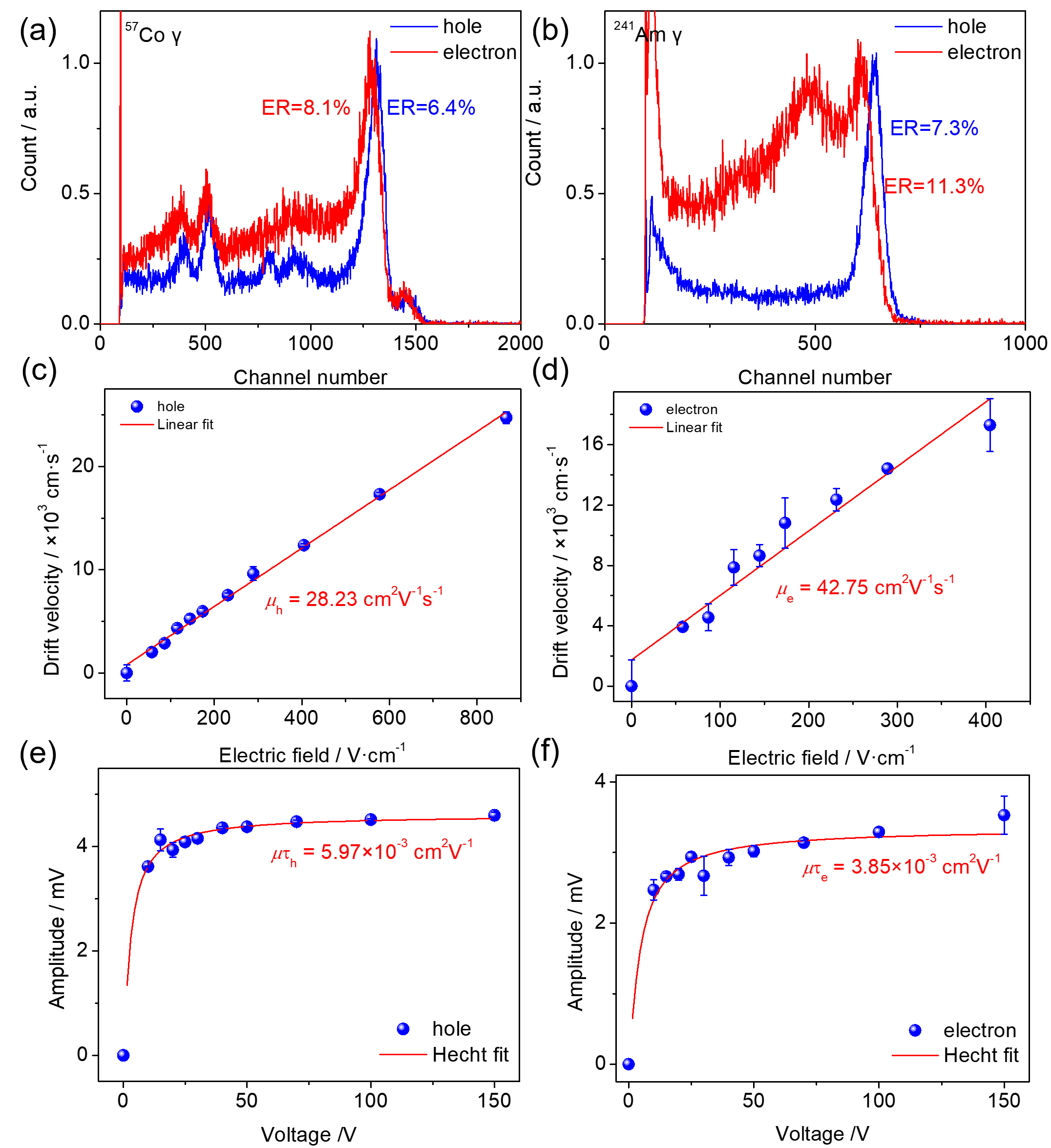
**

**Figure S35.** The detector performance of wafer 7-3-4: (a), (b) ^57^Co and ^241^Am γ-ray spectra obtained by irradiated from anode (hole collection) and cathode (electron collection), respectively. (c), (d) Hole and electron mobilities by linearly fitting the electric field-dependent drift velocity. (e), (f) Hole and electron mobility-lifetime product (*μτ*_h_) according to the Hecht equation.


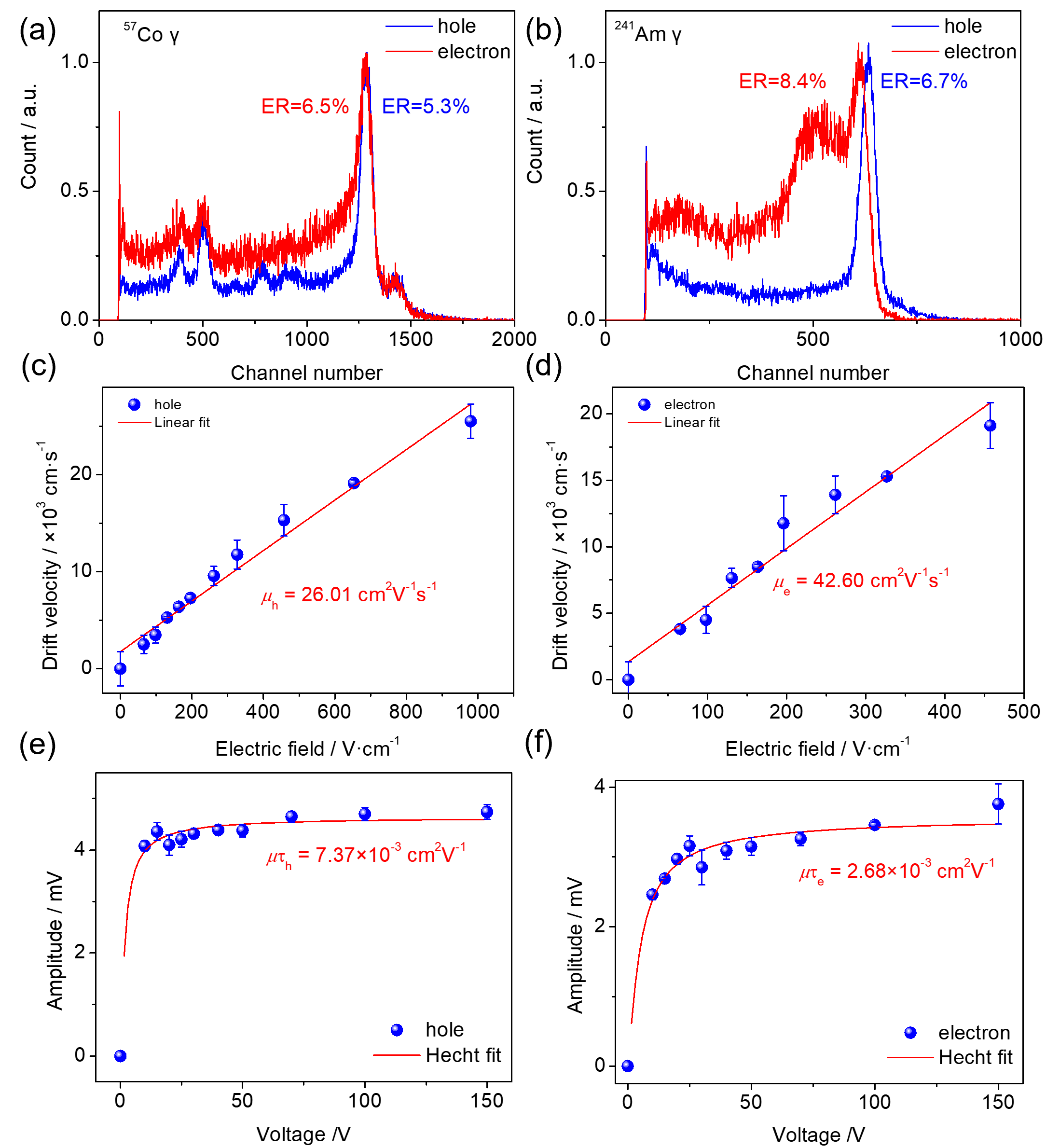


**Figure S36.** The detector performance of wafer 7-3-5: (a), (b) ^57^Co and ^241^Am γ-ray spectra obtained by irradiated from anode (hole collection) and cathode (electron collection), respectively. (c), (d) Hole and electron mobilities by linearly fitting the electric field-dependent drift velocity. (e), (f) Hole and electron mobility-lifetime product (*μτ*_h_) according to the Hecht equation.

**
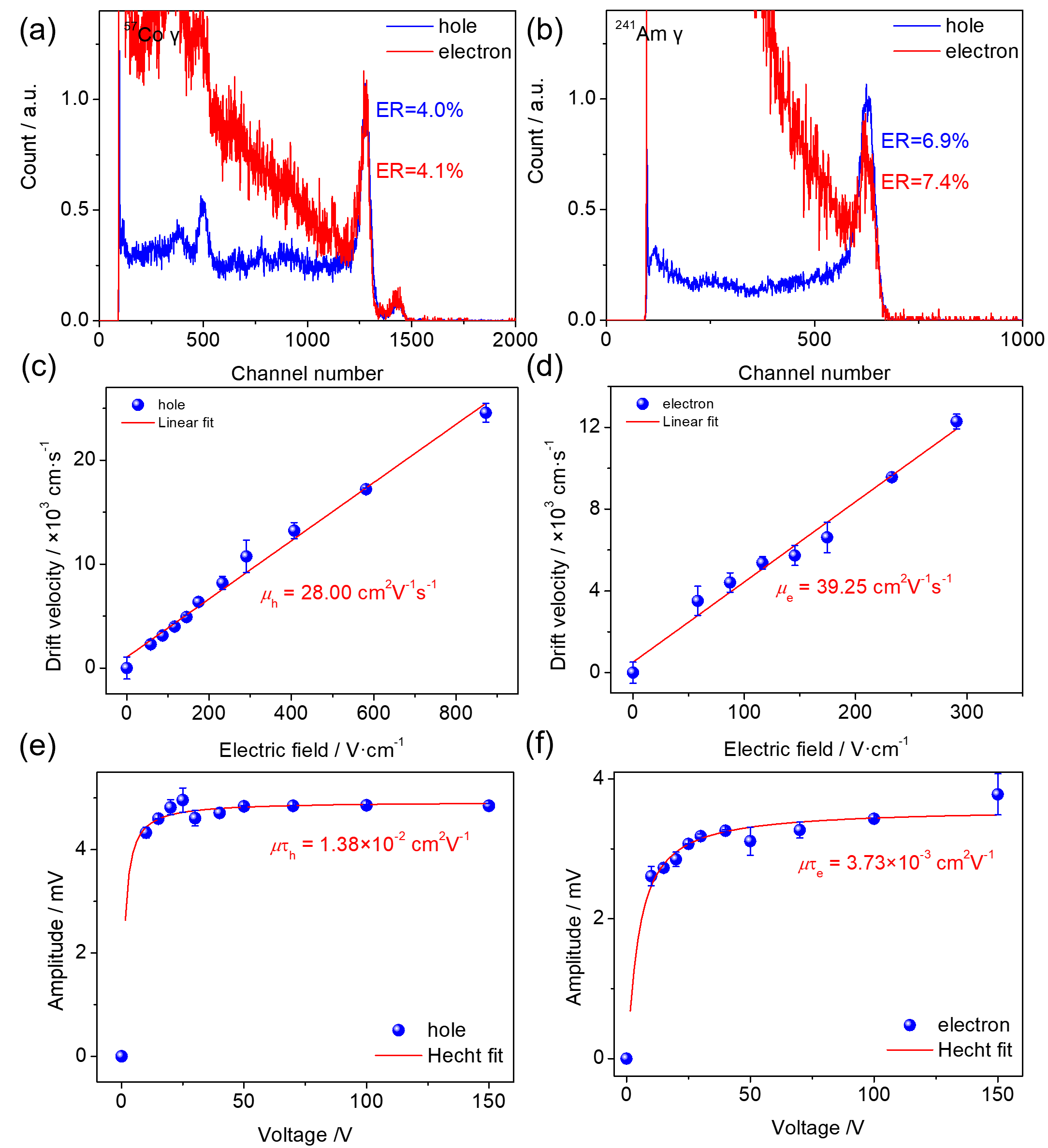
**

**Figure S37.** The detector performance of wafer 7-4-3: (a), (b) ^57^Co and ^241^Am γ-ray spectra obtained by irradiated from anode (hole collection) and cathode (electron collection), respectively. (c), (d) Hole and electron mobilities by linearly fitting the electric field-dependent drift velocity. (e), (f) Hole and electron mobility-lifetime product (*μτ*_h_) according to the Hecht equation.


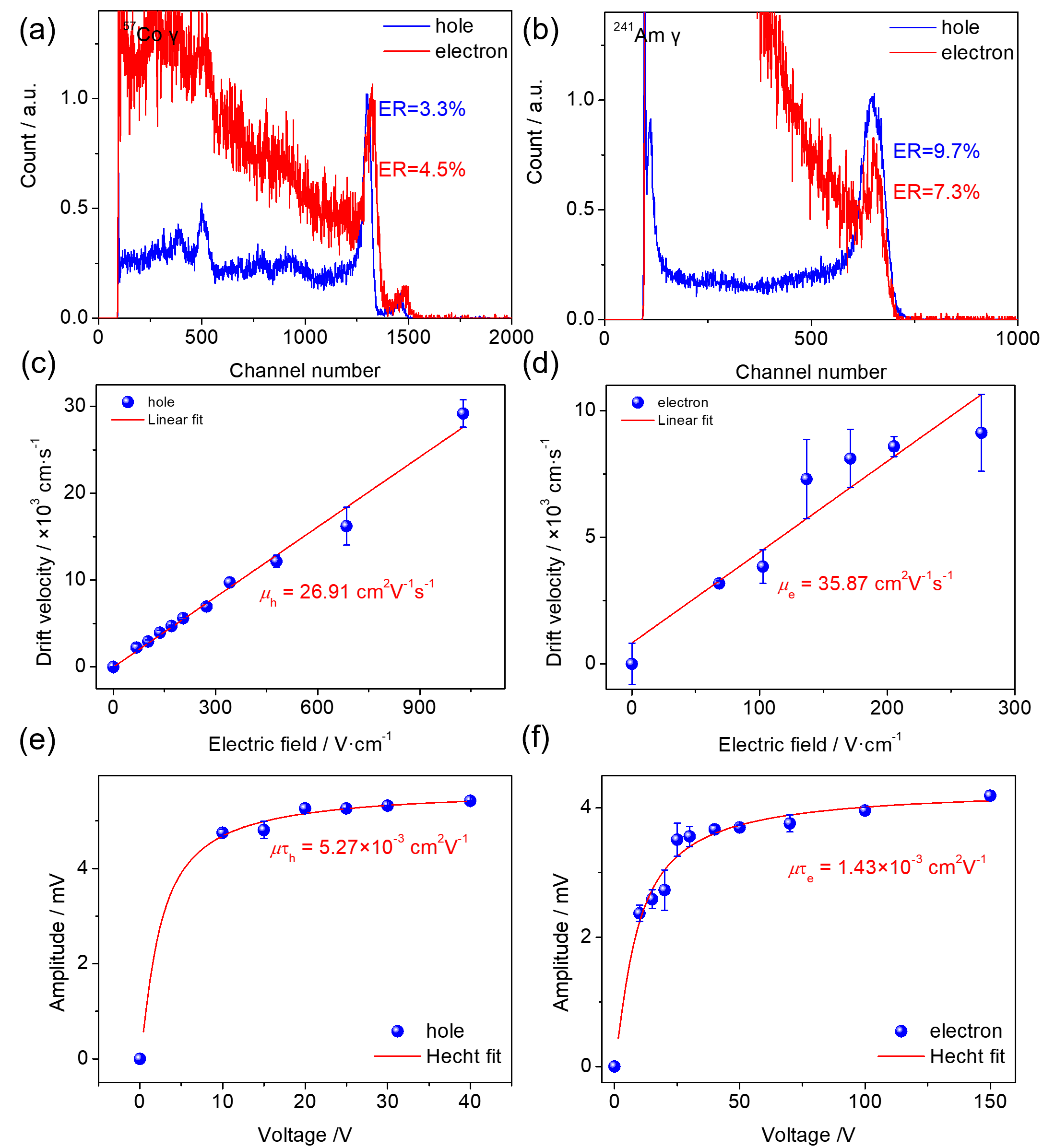


**Figure S38.** The detector performance of wafer 7-5-3: (a), (b) ^57^Co and ^241^Am γ-ray spectra obtained by irradiated from anode (hole collection) and cathode (electron collection), respectively. (c), (d) Hole and electron mobilities by linearly fitting the electric field-dependent drift velocity. (e), (f) Hole and electron mobility-lifetime product (*μτ*_h_) according to the Hecht equation.

**
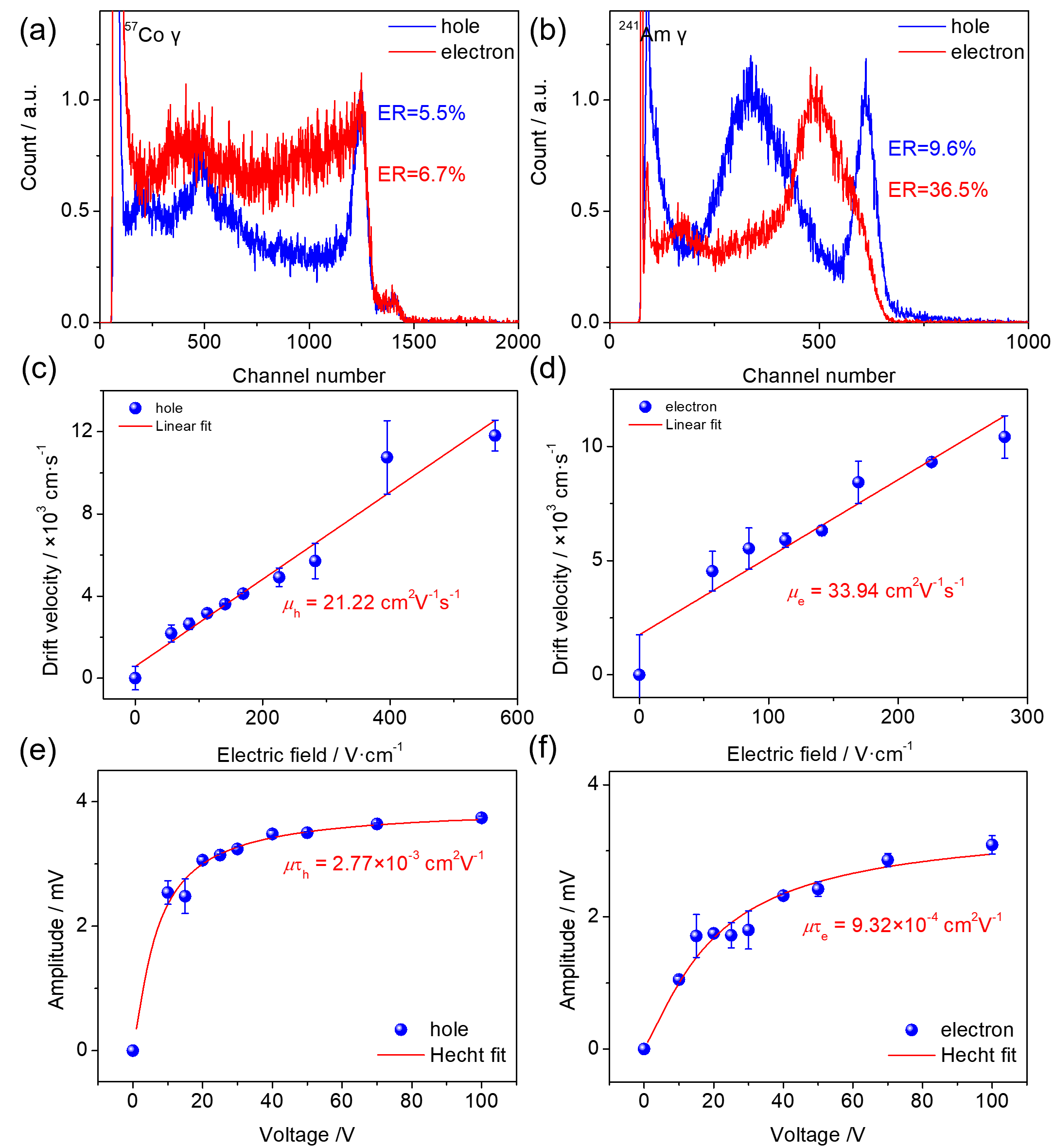
**

**Figure S39.** The detector performance of wafer 9-3-3: (a), (b) ^57^Co and ^241^Am γ-ray spectra obtained by irradiated from anode (hole collection) and cathode (electron collection), respectively. (c), (d) Hole and electron mobilities by linearly fitting the electric field-dependent drift velocity. (e), (f) Hole and electron mobility-lifetime product (*μτ*_h_) according to the Hecht equation.


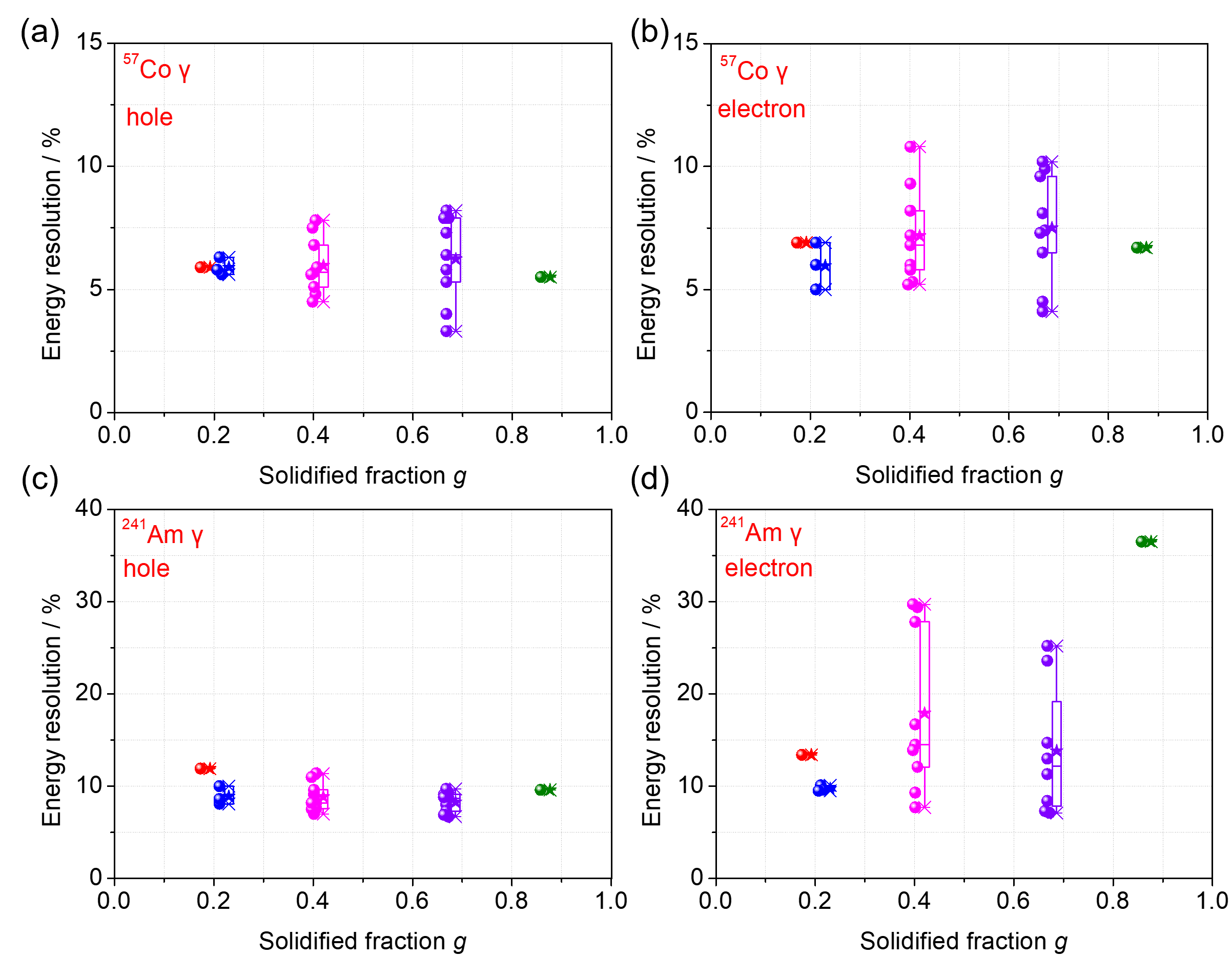


**Figure S40.** The statistical results of energy resolution of hole and electron collection under ^241^Am and ^57^Co γ-ray versus solidified fraction for different CsPbBr_3_ wafers.

**
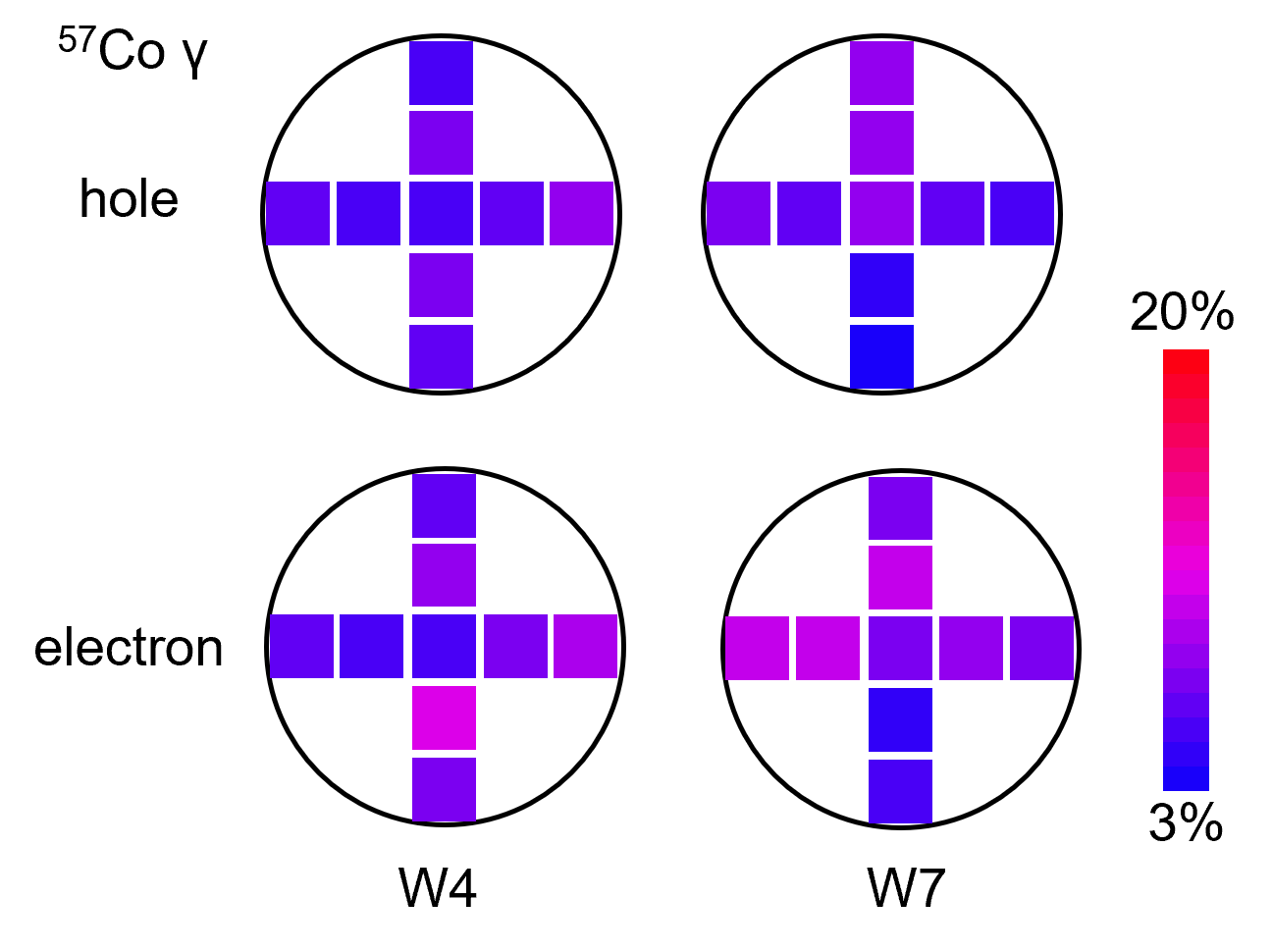
**

**Figure S41.** The hole and electron energy resolution of ^57^Co γ-ray for W4 and W7 wafers, respectively.


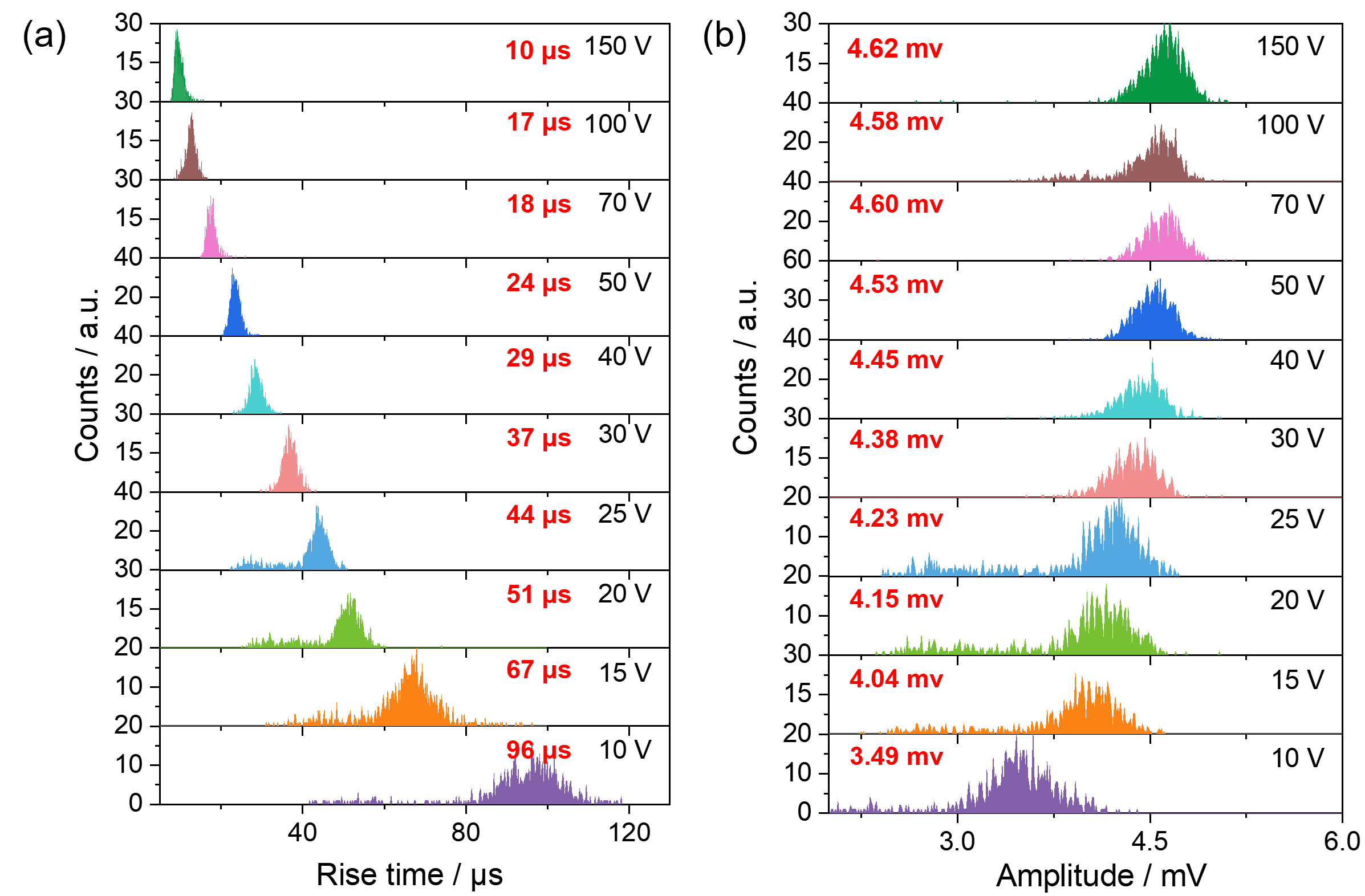


**Figure S42.** The typical distributions of rise time and amplitude of the wafer 4-3-2 under various voltage under hole collection mode by irradiated from anode using ^241^Am γ-ray sources, respectively.


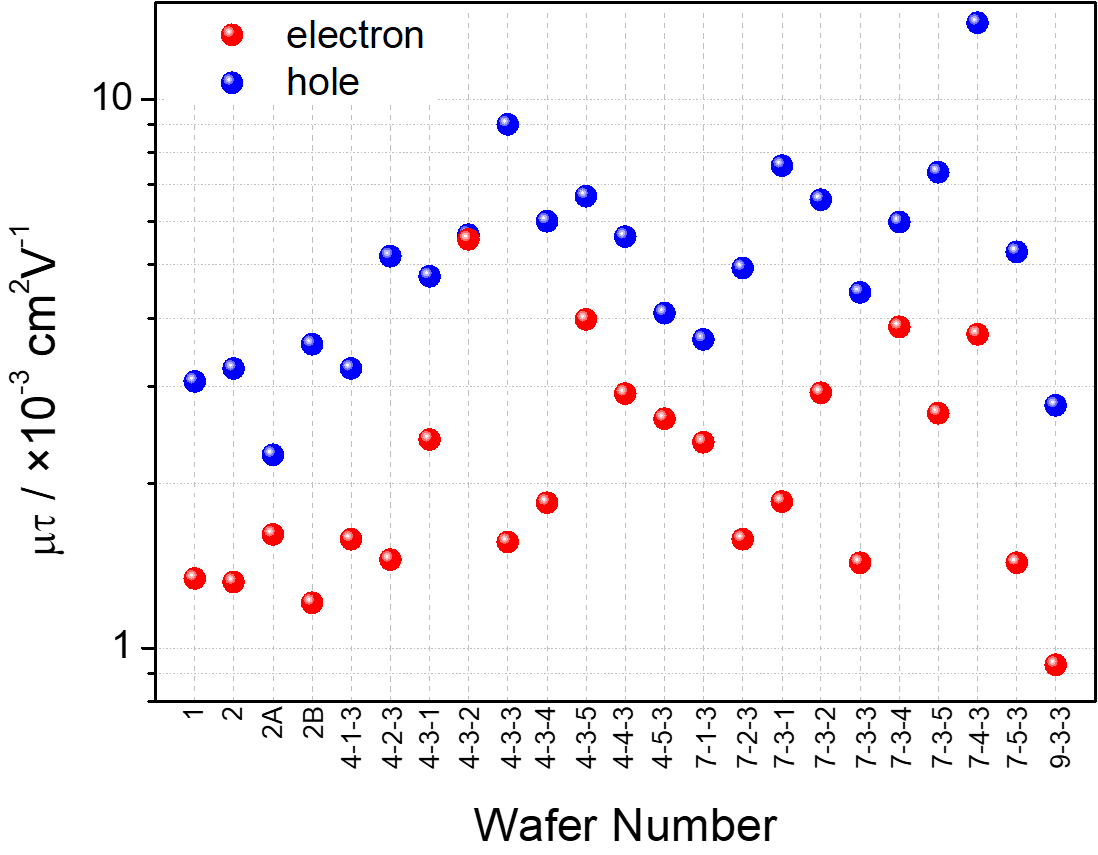


**Figure S43.** Hole and electron mobility-lifetime products for various CsPbBr_3_ wafers.


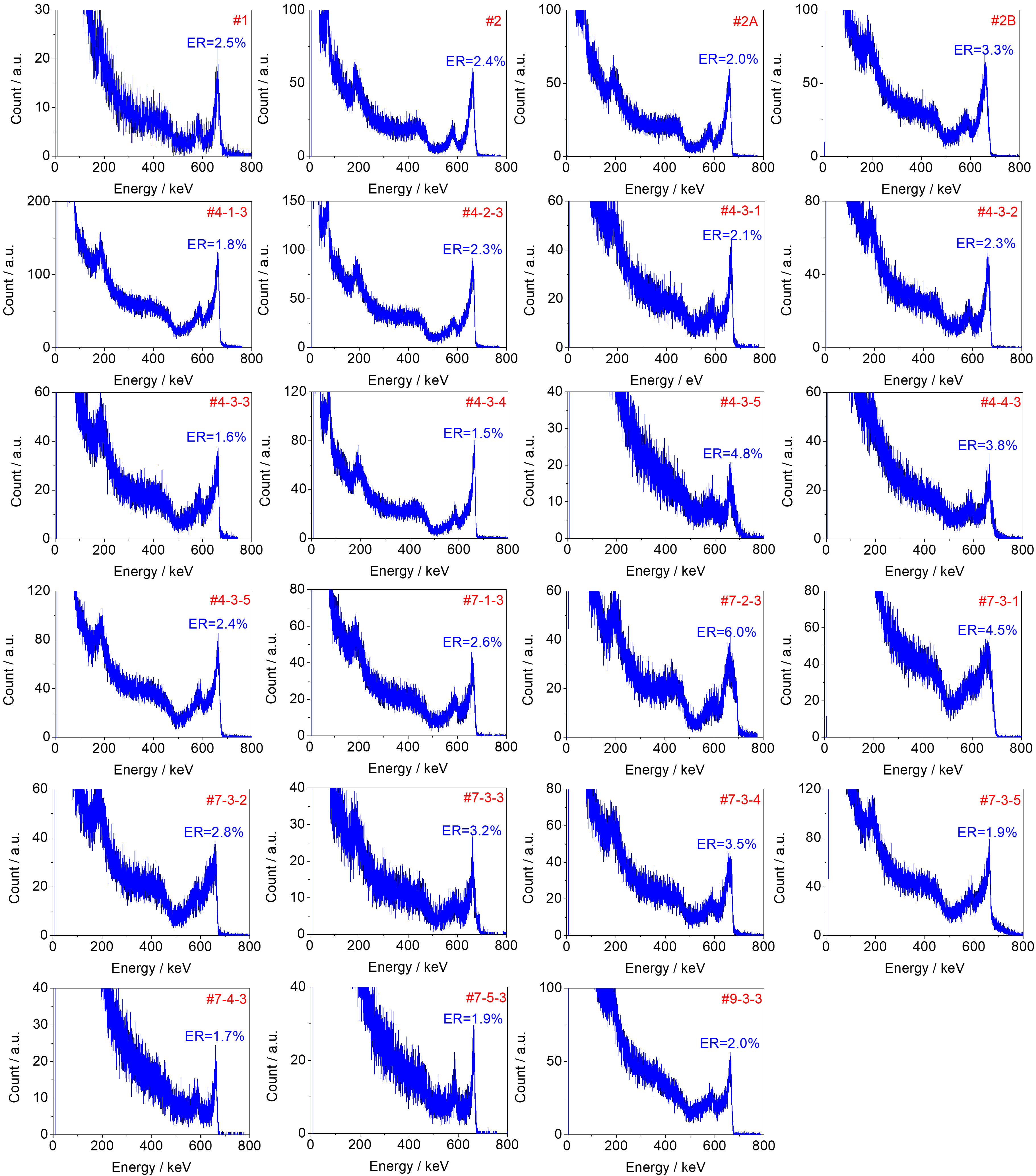


**Figure S44.** ^137^Cs γ-ray spectra of different wafers with the collecting time of 10 min.


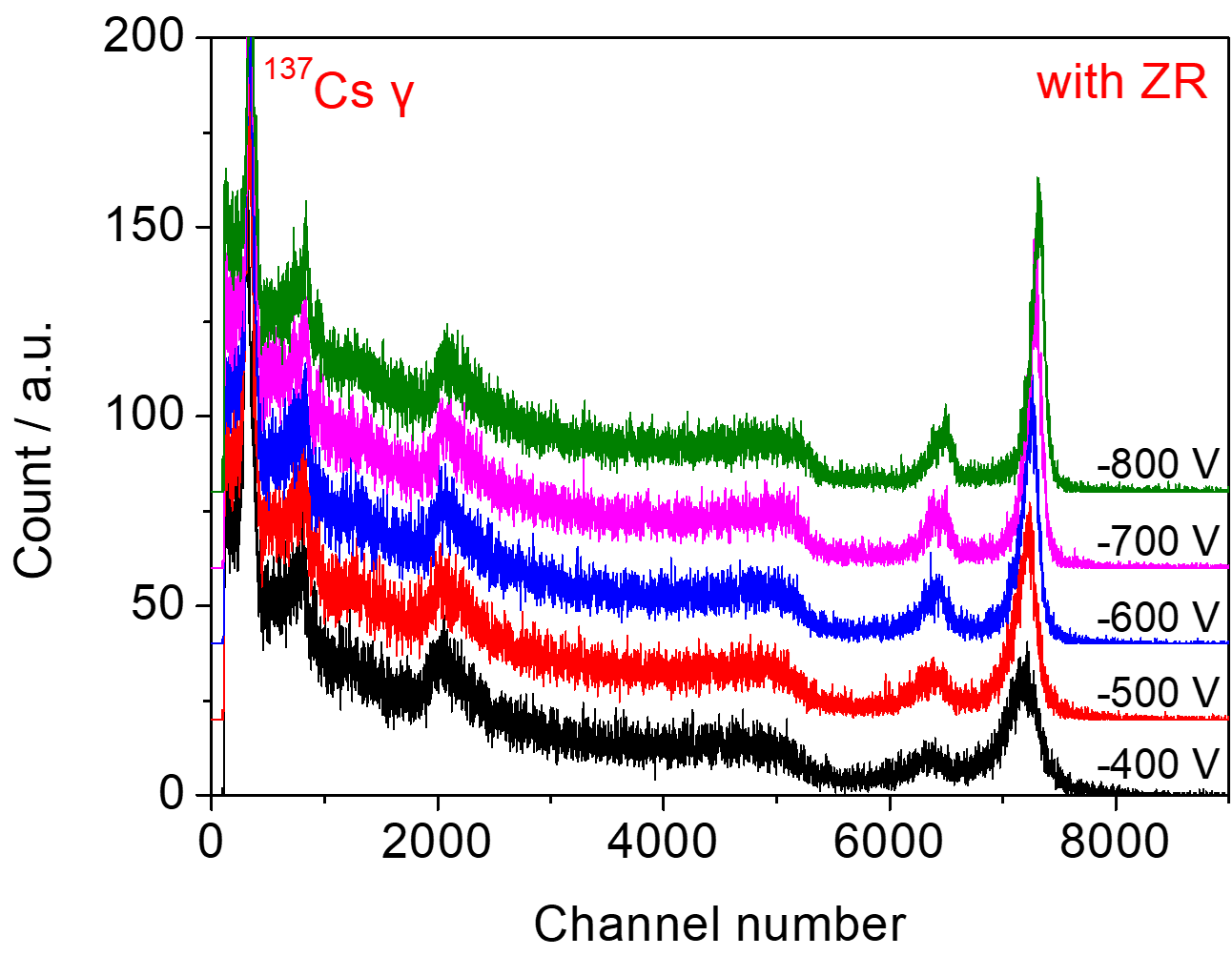


**Figure S45.** ^137^Cs γ-ray spectra of CsPbBr_3_ device with ZR under various voltages. The detector dimension is 5×5.3×1.74 mm^3^.

**
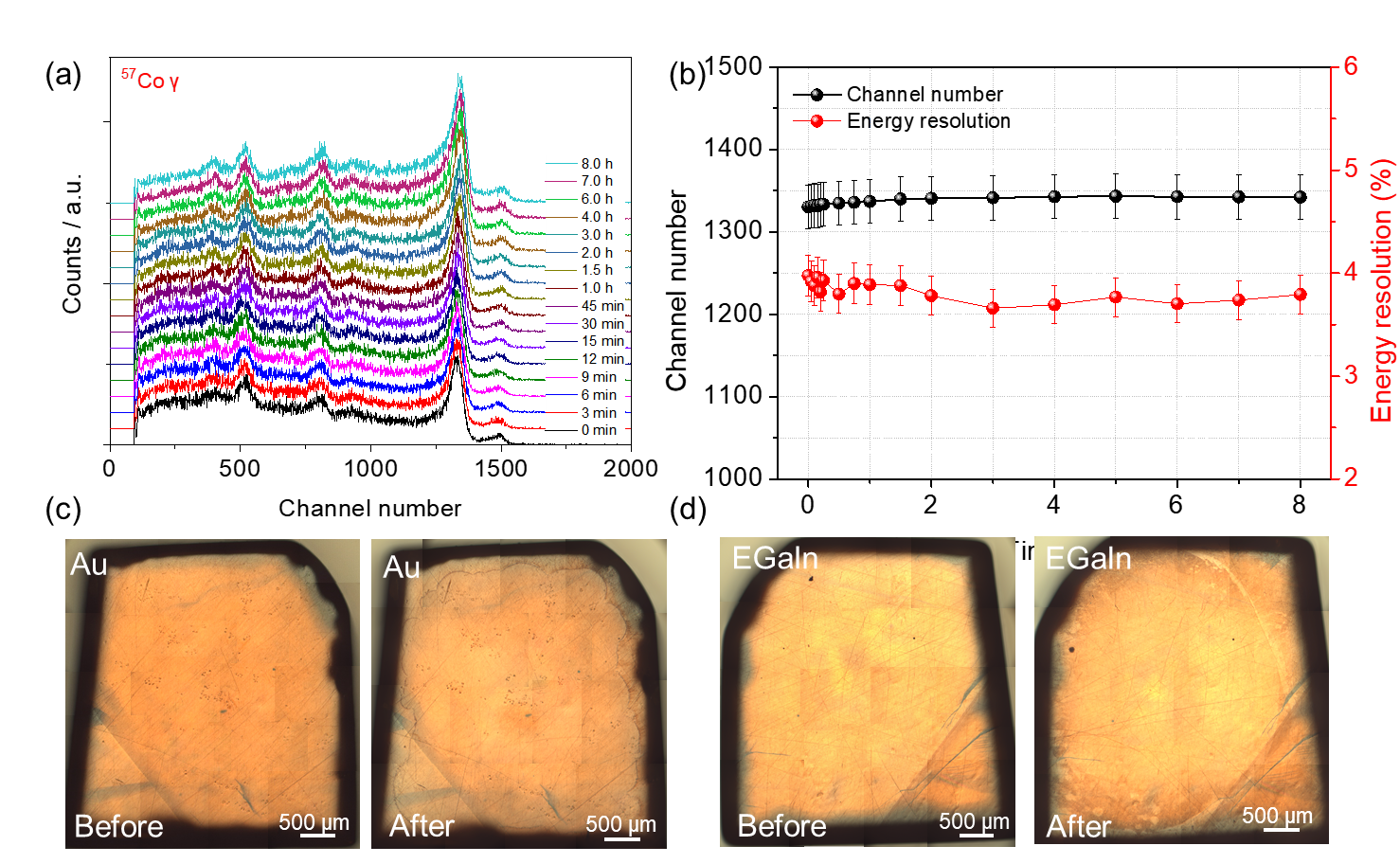
**

**Figure S46.** (a) ^57^Co γ-ray spectra of the CsPbBr_3_ detector (with ZR) with time continuously under the applied voltage of -500 V. (b) The corresponding peak channel number of 122 keV ^57^Co γ-ray and energy resolution over an 8 h period. The error bars for channel and energy resolution represent 2% and 5% errors, respectively. (c)-(d) The crystal surface of Au and EGaIn contacts before and after 8 h biasing.


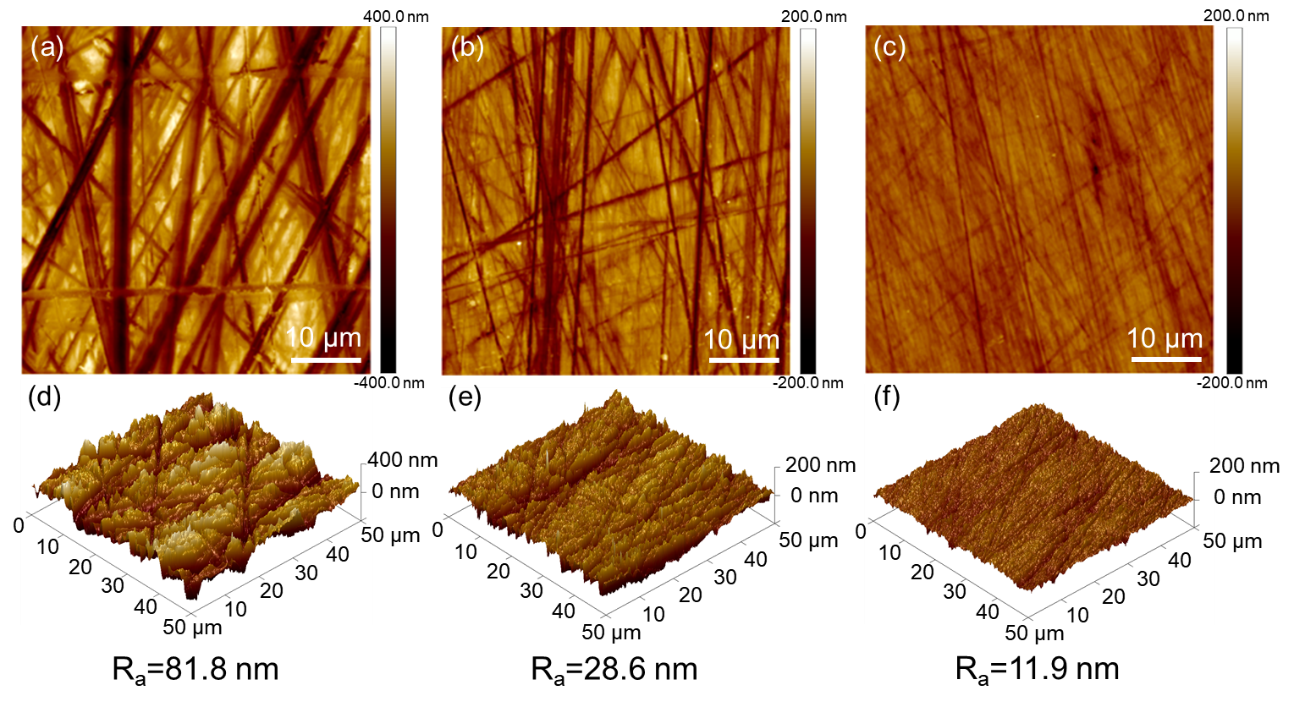


**Figure S47.** Surface roughness by AFM. (a)-(c) 2D AFM images of the crystal surface after polishing by 2000, 5000, and 12000 mesh SiC sandpapers with mineral oil, respectively. (d)-(f) The corresponding 3D AFM images.

**Table S1.** GDMS analysis of as-grown CsPbBr_3_ crystals without ZR. The total impurity level was determined to be 10.48 ppm excluding the elements below the detection limit.

| Elements | Concentration / ppm | Elements | Concentration  / ppm | Elements | Concentration  / ppm |
| --- | --- | --- | --- | --- | --- |
| Li | < 0.005 | Ag | <0.5 | As | <0.05 |
| Be | < 0.005 | Cd | <0.5 | Se | <0.1 |
| B | < 0.01 | In | Binder | Br | Matrix |
| F | <0.1 | Sn | <0.5 | Rb | 0.17 |
| Na | 0.06 | Sb | <0.5 | Sr | 0.21 |
| Mg | 0.04 | Te | <0.1 | Y | <0.01 |
| Al | 0.23 | I | 1.9 | Zr | <0.01 |
| Si | 0.05 | Cs | Matrix | Nb | <0.01 |
| P | <0.01 | Ba | <0.05 | Mo | <0.01 |
| S | 1.8 | La | <0.05 | Ru | <0.01 |
| Cl | 2.6 | Ce | <0.05 | Rh | <0.05 |
| K | <0.5 | Pr | <0.05 | Pd | <0.5 |
| Ca | 3.4 | Nd | <0.05 | W | <0.05 |
| Sc | <0.05 | Sm | <0.05 | Re | <0.05 |
| Ti | 0.02 | Eu | <0.05 | Os | <0.01 |
| V | <0.01 | Gd | <0.01 | Ir | <0.01 |
| Cr | <0.05 | Tb | <0.01 | Pt | <0.1 |
| Mn | <0.01 | Dy | <0.01 | Au | <0.1 |
| Fe | <0.05 | Ho | <0.01 | Hg | <0.1 |
| Co | <0.01 | Er | <0.01 | Tl | <0.1 |
| Ni | <0.01 | Tm | <0.01 | Pb | Matrix |
| Cu | <0.05 | Yb | <0.01 | Bi | <0.1 |
| Zn | <0.01 | Lu | <0.01 | Th | <0.005 |
| Ga | <0.01 | Hf | <0.01 | U | <0.005 |
| Ge | <0.05 | Ta | Source |  |  |

**Table S2.** GDMS analysis of as-grown CsPbBr_3_ crystals with ZR. The total impurity level was determined to be 3.42 ppm excluding the elements below the detection limit.

| Elements | Concentration / ppm | Elements | Concentration  / ppm | Elements | Concentration  / ppm |
| --- | --- | --- | --- | --- | --- |
| Li | 0.02 | Ag | <0.5 | As | <0.05 |
| Be | < 0.005 | Cd | <0.5 | Se | <0.1 |
| B | < 0.01 | In | Binder | Br | Matrix |
| F | <0.1 | Sn | <0.5 | Rb | <0.01 |
| Na | 0.14 | Sb | <0.5 | Sr | <0.01 |
| Mg | 0.03 | Te | <0.1 | Y | <0.01 |
| Al | 0.04 | I | <0.5 | Zr | <0.01 |
| Si | <0.01 | Cs | Matrix | Nb | <0.01 |
| P | <0.01 | Ba | <0.05 | Mo | <0.01 |
| S | 0.9 | La | <0.05 | Ru | <0.01 |
| Cl | 2 | Ce | <0.05 | Rh | <0.05 |
| K | <0.5 | Pr | <0.05 | Pd | <0.5 |
| Ca | 0.29 | Nd | <0.05 | W | <0.05 |
| Sc | <0.05 | Sm | <0.05 | Re | <0.05 |
| Ti | <0.01 | Eu | <0.05 | Os | <0.01 |
| V | <0.01 | Gd | <0.01 | Ir | <0.01 |
| Cr | <0.05 | Tb | <0.01 | Pt | <0.1 |
| Mn | <0.01 | Dy | <0.01 | Au | <0.1 |
| Fe | <0.05 | Ho | <0.01 | Hg | <0.1 |
| Co | <0.01 | Er | <0.01 | Tl | <0.1 |
| Ni | <0.01 | Tm | <0.01 | Pb | Matrix |
| Cu | <0.05 | Yb | <0.01 | Bi | <0.1 |
| Zn | <0.01 | Lu | <0.01 | Th | <0.005 |
| Ga | <0.01 | Hf | <0.01 | U | <0.005 |
| Ge | <0.05 | Ta | Source |  |  |

**Table S3.** The EDS results of elements at different points of CsPbBr_3_ without ZR.

| w/o ZR | Point 1 | Point 2 | Point 3 | Point 4 | Point 5 | Average |
| --- | --- | --- | --- | --- | --- | --- |
| Cs | 19.9 | 19.9 | 20.0 | 19.9 | 19.9 | 19.92 |
| Pb | 20.2 | 20.5 | 20.3 | 20.4 | 20.4 | 20.36 |
| Br | 59.8 | 59.7 | 59.6 | 59.7 | 59.7 | 59.70 |
| Cs:Pb:Br | 1:1.015:3.005 | 1:1.030:3 | 1:1.015:2.980 | 1:1.025:3 | 1:1.025:3 | 1:1.022:2.997 |

**Table S4.** The EDS results of elements at different points of CsPbBr_3_ with ZR.

| with ZR | Point 1 | Point 2 | Point 3 | Point 4 | Point 5 | Average |
| --- | --- | --- | --- | --- | --- | --- |
| Cs | 19.9 | 20.0 | 19.7 | 19.9 | 19.8 | 19.86 |
| Pb | 20.2 | 20.1 | 20.4 | 20.2 | 20.2 | 20.22 |
| Br | 59.9 | 59.8 | 59.9 | 59.9 | 59.9 | 59.88 |
| Cs:Pb:Br | 1:1.015:3.010 | 1:1.005:2.99 | 1:1.036:3.041 | 1:1.015:3.010 | 1:1.020:3.025 | 1:1.018:3.015 |

**Table S5.** Summary of crystal properties and γ-ray performance without and with ZR in planar configuration.

| Crystal | Detector | *d*  (mm) | *S*  (mm^2^) | *ρ*  (Ω cm) | *μ*_h_  (cm^2^∙V^-1^∙s^-1^) | *μτ*_h_  (cm^2^∙V^-1^) | Energy resolution for γ-rays | | | |
| --- | --- | --- | --- | --- | --- | --- | --- | --- | --- | --- |
|  |  |  |  |  |  |  | ^241^Am | ^57^Co | ^22^Na |  |
| w/o ZR | I | 0.80 | 4.00 | 4.86×10^9^ | 12.55 | 9.22×10^-4^ | 11.7% | 8.0% | 8.3% |  |
|  | II | 1.20 | 4.80 | / | / | / | 15.3% | 8.5% | / |  |
|  | III | 1.10 | 8.50 | / | / | / | 23.7% | 17.6% | / |  |
|  | IV | 1.10 | 12.87 | / | / | / | 28.1% | 13.8% | / |  |
| with ZR | I | 1.27 | 14.00 | 8.31×10^9^ | 26.60 | 8.36×10^-3^ | 6.4% | 3.1% | 2.5% |  |
|  | II | 1.32 | 17.63 | / | / | / | 8.5% | 4.9% | 2.7% |  |
|  | III | 2.20 | 23.46 | / | / | / | 9.9% | 5.1% | 2.6% |  |
|  | IV | 1.05 | 5.40 | / | / | / | 6.9% | 4.0% | / |  |

**Table S6.** The distance of cutting wafers to the tip of CsPbBr_3_ ingot with ZR.

| Wafer | Distance to ingot tip (*x*) | Solidified fraction (*g*=*x*/*L*) |
| --- | --- | --- |
| W1 | 12.5 mm | 0.170 |
| W2 | 15.1 mm | 0.205 |
| W4 | 29.1 mm | 0.396 |
| W7 | 49.0 mm | 0.666 |
| W9 | 62.6 mm | 0.852 |

*L* is the length of the ingot with the value of 74 mm.

**Table S7.** Summary of detector performance for different wafers of CsPbBr_3_ ingot with ZR.

| Wafer | *d*  (mm) | ER for ^57^Co γ | | ER for ^241^Am γ | | ER for ^137^Cs γ | *μ*_h_  (cm^2^V^-1^s^-1^) | *μ*_e_  (cm^2^V^-1^s^-1^) | *μτ*_h_  (cm^2^V^-1^) | *μτ*_e_  (cm^2^V^-1^) |
| --- | --- | --- | --- | --- | --- | --- | --- | --- | --- | --- |
|  |  | hole | electron | hole | electron |  |  |  |  |  |
| 1 | 1.28 | 5.9% | 6.9% | 11.9% | 13.4% | 2.5% | 24.26 | 42.10 | 3.06×10^-3^ | 1.34×10^-3^ |
| 2 | 1.33 | 5.6% | 6.9% | 10.0% | 10.1% | 2.4% | 21.48 | 35.73 | 3.23×10^-3^ | 1.32×10^-3^ |
| 2A | 1.31 | 5.8% | 5.0% | 8.1% | 9.6% | 2.0% | 20.96 | 39.34 | 2.25×10^-3^ | 1.61×10^-3^ |
| 2B | 1.72 | 6.3% | 6.0% | 8.6% | 9.5% | 3.3% | 28.53 | 43.44 | 3.58×10^-3^ | 1.21×10^-3^ |
| 4-1-3 | 1.86 | 4.8% | 6.0% | 8.2% | 29.4% | 1.8% | 26.20 | 43.01 | 3.23×10^-3^ | 1.58×10^-3^ |
| 4-2-3 | 1.82 | 6.8% | 8.2% | 7.6% | 16.7% | 2.3% | 24.68 | 39.89 | 5.18×10^-3^ | 1.45×10^-3^ |
| 4-3-1 | 1.71 | 5.7% | 5.8% | 9.1% | 9.3% | 2.1% | 22.29 | 41.75 | 4.76×10^-3^ | 2.40×10^-3^ |
| 4-3-2 | 1.84 | 5.1% | 5.3% | 7.0% | 7.7% | 2.3% | 25.41 | 43.33 | 5.67×10^-3^ | 5.56×10^-3^ |
| 4-3-3 | 1.51 | 4.5% | 5.2% | 7.5% | 27.8% | 1.6% | 25.43 | 40.37 | 9.00×10^-3^ | 1.56×10^-3^ |
| 4-3-4 | 1.53 | 5.6% | 6.8% | 8.2% | 14.5% | 1.5% | 22.29 | 33.99 | 5.99×10^-3^ | 1.84×10^-3^ |
| 4-3-5 | 1.88 | 7.8% | 9.3% | 11.4% | 12.1% | 4.8% | 27.53 | 41.30 | 6.66×10^-3^ | 3.97×10^-3^ |
| 4-4-3 | 1.83 | 7.5% | 10.8% | 11% | 13.9% | 3.8% | 25.11 | 45.60 | 5.62×10^-3^ | 2.91×10^-3^ |
| 4-5-3 | 1.76 | 5.9% | 7.2% | 9.6% | 29.7% | 2.4% | 23.49 | 42.02 | 4.08×10^-3^ | 2.62×10^-3^ |
| 7-1-3 | 1.48 | 7.9% | 7.4% | 9.3% | 25.2% | 2.6% | 21.29 | 40.62 | 3.65×10^-3^ | 2.37×10^-3^ |
| 7-2-3 | 1.44 | 8.2% | 9.9% | 8.7% | 13.0% | 6.0% | 26.03 | 38.60 | 4.93×10^-3^ | 1.58×10^-3^ |
| 7-3-1 | 1.63 | 7.3% | 9.6% | 9.1% | / | 4.5% | 23.49 | 42.66 | 7.57×10^-3^ | 1.85×10^-3^ |
| 7-3-2 | 1.66 | 5.8% | 10.2% | 8.0% | 23.6% | 2.8% | 26.28 | 44.31 | 6.56×10^-3^ | 2.92×10^-3^ |
| 7-3-3 | 1.53 | 7.9% | 7.3% | 8.8% | 14.7% | 3.2% | 25.24 | 30.27 | 4.45×10^-3^ | 1.43×10^-3^ |
| 7-3-4 | 1.73 | 6.4% | 8.1% | 7.3% | 11.3% | 3.5% | 28.23 | 42.75 | 5.97×10^-3^ | 3.85×10^-3^ |
| 7-3-5 | 1.53 | 5.3% | 6.5% | 6.7% | 8.4% | 1.9% | 26.01 | 42.60 | 7.37×10^-3^ | 2.68×10^-3^ |
| 7-4-3 | 1.72 | 4.0% | 4.1% | 6.9% | 7.1% | 1.7% | 28.00 | 39.25 | 13.8×10^-3^ | 3.73×10^-3^ |
| 7-5-3 | 1.46 | 3.3% | 4.5% | 9.7% | 7.3% | 1.9% | 26.91 | 35.87 | 5.27×10^-3^ | 1.43×10^-3^ |
| 9-3-3 | 1.77 | 5.5% | 6.7% | 9.6% | 36.5% | 2.0% | 21.22 | 33.94 | 2.77×10^-3^ | 0.93×10^-3^ |

**Table S8.** Comparison of perovskite detector performances in resolving ^137^Cs 662 keV γ-ray.

| Materials | Device architecture | Electric field (Vcm^-1^) | ER for ^137^Cs γ | Reference |
| --- | --- | --- | --- | --- |
| CsPbBr_3_ | Planar | 4597.7 | 1.3% | this work |
|  |  | 1100~2100 | 1.5%~6% |  |
|  |  | 7258.1 | 3.8% | ^[1]^ |
|  |  | 2800 | 5.5% | ^[2]^ |
|  |  | 3759.4 | 1.4% | ^[3]^ |
|  |  | 583.3 | 2.0% | ^[4]^ |
|  |  | 1666.7 | 12.2% | ^[5]^ |
|  |  | 1521.7 | 11% | ^[6]^ |
|  |  | 1976.3 | 5.5% | ^[7]^ |
|  |  | 4000 | 12.85% | ^[8]^ |
|  |  | 3000 | 7.2% | ^[9]^ |
|  |  | 2666.7 | 13.3% | ^[10]^ |
|  | Quasi-hemispherical | 5298 | 1.8% | ^[3]^ |
|  |  | 500 | 9.91% | ^[11]^ |
|  |  | 1250 | 11.47% | ^[12]^ |
|  | Pixelated | 2892.6 | 7.31% | ^[13]^ |
|  |  | 1077.6 | 1.4% | ^[3]^ |
| MAPbBr_3_ | Planar | 400 | 3.9% | ^[14]^ |
| MAPbBr_2.94_Cl_0.06_ | Planar | 18 | 6.5% | ^[15]^ |
| FA_0.9_Cs_0.1_PbBr_3_ | Planar | 5000 | 2.9% | ^[16]^ |
| FAPbBr_3_ | Planar | 4000 | 1.7% | ^[17]^ |

**References**

[1] Y. He, L. Matei, H. J. Jung, K. M. McCall, M. Chen, C. C. Stoumpos, Z. Liu, J. A. Peters, D. Y. Chung, B. W. Wessels, M. R. Wasielewski, V. P. Dravid, A. Burger, M. G. Kanatzidis, *Nat. Commun.* **2018**, 9, 1609.

[2] Y. Feng, L. Pan, H. Wei, Y. Liu, Z. Ni, J. Zhao, P. N. Rudd, L. R. Cao, J. Huang, *J. Mater. Chem. C* **2020**, 8, 11360.

[3] Y. He, M. Petryk, Z. Liu, D. G. Chica, I. Hadar, C. Leak, W. Ke, I. Spanopoulos, W. Lin, D. Y. Chung, B. W. Wessels, Z. He, M. G. Kanatzidis, *Nat. Photonics* **2021**, 15, 36.

[4] R. Toufanian, S. Swain, P. Becla, S. Motakef, A. Datta, *J. Mater. Chem. C* **2022**, 10, 12708.

[5] Y. Hao, F. Li, R. Bai, X. Zhang, Q. Sun, W. Jie, Y. Xu, *IEEE Trans. Electron Devices* **2022**, 69, 6837.

[6] L. Pan, Y. Feng, J. Huang, L. R. Cao, *IEEE Trans. Nucl. Sci.* **2020**, 67, 2255.

[7] L. Pan, Y. Feng, P. Kandlakunta, J. Huang, L. R. Cao, *IEEE Trans. Nucl. Sci.* **2020**, 67, 443.

[8] M. Zhang, C. Huang, G. Xia, J. Liu, F. Tian, J. Zou, B. Tang, *Acta Crystallogr., Sect. B: Struct. Sci., Cryst. Eng. Mater.* **2024**, B80, 64.

[9] R. Bai, B. Ge, X. Liu, X. Peng, X. Zhang, S. Liu, M. Zhu, C. Zhou, A. Dubois, W. Jie, Y. Xu, *J. Mater. Chem. A* **2024**, 12, 13925.

[10] Y. Hao, R. Bai, X. Zhang, T. Wang, W. Liu, A. Dubois, W. Jie, Y. Xu, *Appl. Phys. Lett.* **2024**, 125, 102106.

[11] Q. Sun, B. Ge, B. Xiao, F. Li, L. Ji, Z. Yin, J. Guo, J. Tang, C. Zhou, W. Jie, M. Zhu, Y. Xu, *Adv. Sci.* **2023**, 10, 2302236.

[12] X. Zhang, F. Li, R. Bai, Q. Sun, Y. Hao, S. Xi, M. Zhu, S. Jiang, W. Jie, Y. Xu, *J. Mater. Chem. C* **2022**, 10, 6107.

[13] X. Zhang, F. Li, Y. Hao, R. Bai, Y. Xin, Q. Sun, X. Ouyang, W. Jie, Y. Xu, *IEEE Trans. Electron Devices* **2023**, 70, 5190.

[14] Z. Ni, L. Zhao, Z. Shi, A. Singh, J. Wiktor, M. O. Liedke, A. Wagner, Y. Dong, M. C. Beard, D. J. Keeble, J. Huang, *Adv. Mater.* **2024**, 36, 2406193.

[15] H. T. Wei, D. DeSantis, W. Wei, Y. H. Deng, D. Y. Guo, T. J. Savenije, L. Cao, J. S. Huang, *Nat. Mater.* **2017**, 16, 826.

[16] L. Zhao, Y. Zhou, Z. Shi, Z. Ni, M. Wang, Y. Liu, J. Huang, *Nat. Photonics* **2023**, 17, 315.

[17] L. Zhao, Z. Shi, Y. Zhou, X. Wang, Y. Xian, Y. Dong, O. Reid, Z. Ni, M. C. Beard, Y. Yan, J. Huang, *Nat. Photonics* **2024**, 18, 250.
